# Moth‐Eye‐Engineered Flexible Films for X‐Ray Shielding and Persistent Radiation Warning

**DOI:** 10.1002/advs.202514035

**Published:** 2025-10-13

**Authors:** Yuansheng Jiang, Wen‐Guang Li, Xiuji Yi, Meifang Yang, Xinyi Lin, Yaxun Hu, Yicheng Yuan, Qiang Ma, Yuping Li, Fengyun Wang, Qin Xu, Wenjing Zhang, Yu‐Xin Chen, Tian Tian, Huan Pang

**Affiliations:** ^1^ School of Chemistry and Materials Yangzhou University Yangzhou Jiangsu 225002 P. R. China; ^2^ Department of Neurosurgery Northern Jiangsu People's Hospital Affiliated to Yangzhou University Yangzhou China; ^3^ College of Physics Qingdao University Qingdao 266071 China; ^4^ Department of Environmental and Resource Engineering Technical University of Denmark Kongens Lyngby 2800 Denmark; ^5^ GBRCE for Functional Molecular Engineering Instrumental Analysis & Research Center School of Chemical Engineering and Technology Sun Yat‐sen University Guangzhou 510275 P. R. China

**Keywords:** electrospinning, flexible scintillators, large‐area film, moth‐eye morphology, radiation shielding, rare‐earth

## Abstract

Developing flexible radiation detectors that maintain high performance under harsh environmental conditions remains a significant materials challenge. Conventional flexible scintillators often sacrifice either performance or stability. The study designed bioinspired SrAl_2_O_4_:Eu^2+^, Dy^3+^@SiO_2_ (SOD@SiO_2_) composites, where strong Al─O─Si covalent bonds created a unique moth‐eye morphology. SOD@SiO_2_ films produced through a scalable electrospinning process demonstrate excellent resistance to water, acids, and alkalis, ensuring stable performance in harsh environments. Their flexibility further enhances applicability in complex 3D structures. Comprehensive testing confirms that these films combine multiple advanced functions, including high X‐ray shielding efficiency (99.80% attenuation, equivalent to 0.35 mm Pb), ultrasensitive detection of low‐dose rate X‐rays (0.43 µGy s^−1^), high‐resolution X‐ray imaging (9.6 lp mm^−1^), and prolonged radiation‐induced visual warning lasting up to 20 h. This multifunctionality—unachievable with conventional SOD or lead‐based protective materials—provides a promising platform for developing next‐generation lightweight materials for radiation shielding, detection, and early warning.

## Graphical abstract

A moth‐eye‐engineered film SrAl_2_O_4_:Eu^2+^, Dy^3+^@SiO_2_ (SOD@SiO_2_) that integrates X‐ray shielding, persistent luminescence, and high‐resolution imaging. It bridges nighttime maritime rescue, radiation protection, and real‐time X‐ray hazard alerts, overcoming traditional trade‐offs between performance and environmental resilience.

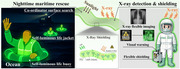

## 1 Introduction

X‐ray, as a pivotal tool in modern science and technology, has found widespread application in medical diagnostics,^[^ ^1^ ^]^ industrial non‐destructive testing,^[^ ^2^ ^]^ and public safety.^[^ ^3^ ^]^ However, as a form of high‐energy radiation that is imperceptible to the human eye, X‐ray poses potential health risks in the event of leakage. This underscores the importance of both radiation shielding and real‐time monitoring during their use.^[^ ^4^ ^]^ Although a variety of commercial radiation detectors are currently available, they are often hindered by drawbacks such as high cost, reliance on external power sources, limited portability, and poor responsiveness to low‐dose exposure.^[^ ^5^, ^6^, ^7^, ^8^ ^]^ In contrast, direct visualization of X‐ray exposure through the radioluminescence (RL) of scintillators under low‐dose excitation offers a more intuitive and effective alternative for radiation warning. Complementary to radiation leakage monitoring, effective radiation shielding remains equally critical. Conventional X‐ray protective garments are typically composed of lead‐based materials, which, although capable of providing shielding efficiencies greater than 99%, are often inflexible and uncomfortable. The use of lead also raises concerns about health and the environment. Recent advances in lead‐free flexible shielding materials, particularly organic–inorganic hybrid systems, have garnered significant attention.^[^ ^9^, ^10^ ^]^ These materials offer a combination of excellent shielding properties, mechanical flexibility, and additional benefits such as lightweight design, transparency, and functional integration.^[^ ^11^, ^12^ ^]^ Despite progress in flexible radiation protection, challenges remain in integrating radiation signal detection and warning response with shielding performance. Specifically, they offer no capability for real‐time X‐ray leakage alerts. Therefore, there is an urgent need in both medical and industrial fields for the development of flexible protective materials that combine effective radiation shielding with real‐time warning capabilities.

To address this interdisciplinary challenge, energy‐storage visualization technologies based on rare‐earth persistent luminescent materials have shown considerable potential.^[^ ^13^ ^]^ These materials can store energy upon low‐dose X‐ray irradiation and gradually release visible light over extended periods, making them ideal candidates for the development of integrated systems that combine prolonged visual alerts with radiation shielding.^[^ ^1^, ^14^, ^15^ ^]^ From an imaging perspective, their long afterglow lifetimes not only mitigate radiation‐induced damage to biological subjects by allowing delayed readouts,^[^ ^16^ ^]^ but also extend the detection window and significantly enhance imaging efficiency.^[^ ^17^ ^]^ For instance, Zhang et al. reported an X‐ray‐activated rare‐earth nanocrystal system (NaYF:Er@NaYF) capable of tunable near‐infrared afterglow emission, thereby improving signal‐to‐noise ratio and image contrast under X‐ray excitation.^[^ ^18^ ^]^ Among various candidates, SrAl_2_O_4_:Eu^2+^, Dy^3+^ (SOD) has attracted attention due to its high brightness and long persistent luminescence (LPL), making it a promising material for low‐dose X‐ray detection applications.^[^ ^19^, ^20^, ^21^ ^]^ However, SOD suffers from limited X‐ray sensitivity and is prone to degradation in the presence of moisture, oxygen, or prolonged light exposure.^[^ ^22^ ^]^ Its inherent rigidity and mechanical brittleness further hinder its integration into flexible films.^[^ ^23^, ^24^ ^]^ Additionally, when incorporated into polymer matrices, the refractive index (RI) mismatch between SOD and the matrix often leads to significant transparency loss,^[^ ^25^, ^26^ ^]^ impeding the development of transparent scintillating fabrics.^[^ ^27^, ^28^, ^29^, ^30^ ^]^ Thus, a critical challenge remains in the design of rare‐earth‐based scintillating films that are simultaneously transparent, flexible, and scalable through optimized material‐structure engineering.^[^ ^21^, ^31^ ^]^ To address interface challenges, researchers have applied strategies such as multilayer dielectric coatings and scattering nanostructures to reduce refractive index mismatch.^[^ ^32^, ^33^ ^]^ Nonetheless, these methods remain constrained by limited compatibility with flexible substrates, angular dependence, and difficulties in scalable fabrication.^[^ ^34^ ^]^ Recently, biomimetic moth‐eye structures have shown significant promise in flexible optoelectronics, as their subwavelength features enable broadband and wide‐angle refractive index gradients.^[^ ^35^, ^36^ ^]^ This approach provides an effective route for fabricating scalable, high‐performance rare‐earth‐based luminescent films.

In this study, we employed biomimetic design and interfacial engineering to coat SOD powder with SiO_2_ nanospheres, creating a moth‐eye‐engineered SOD@SiO_2_ powder. This biomimetic strategy creates a graded RI interface on the SOD surface, overcoming the transparency challenges caused by RI mismatch between SOD and the polymer. The formation of Al─O─Si bonds between SiO_2_ and SOD induces more oxygen vacancies (OVs), significantly enhancing light capture and resulting in a fourfold increase in photoluminescence (PL) intensity. More importantly, SOD@SiO_2_ exhibits a high light yield (LY) of up to 50 752 photons MeV^−1^ with a low limit of detection (LoD) of 0.36 µGy s^−1^, representing a 90.66% improvement in LY compared to bare SOD. By integrating SOD@SiO_2_ powder uniformly into a composite matrix of poly(methyl methacrylate) (PMMA), thermoplastic polyurethane (TPU), and the trifunctional nitrogenous crosslinker (TTMAP) via electrospinning, we successfully fabricated a flexible, transparent, and large‐area film exhibiting 94.55% visible light transmittance and LPL lasting 20 h. Benefiting from the protective encapsulation achieved through electrospinning, the SOD@SiO_2_ film demonstrates excellent water resistance along with strong tolerance to both acid (pH 0.7) and alkali (pH 12.3) environments, ensuring reliable performance under extreme conditions. Furthermore, the SOD@SiO_2_ film serves as a flexible scintillator screen capable of delivering high‐resolution X‐ray imaging with spatial resolution up to 9.6 lp mm^−1^, while maintaining excellent conformability to curved and complex‐shaped objects. Remarkably, the film combines X‐ray‐induced LPL lasting up to 20 h with 99.80% X‐ray shielding efficiency. This “radiation‐visual alert‐protection” integrated fabric overcomes the functional limitations of conventional shielding clothing, representing a technological paradigm shift from passive shielding to active X‐ray detection with real‐time altering. By integrating our self‐developed 3D image modeling software with a robotic system equipped with SOD@SiO_2_ film, we propose an X‐ray‐based 3D reconstruction and visualization system incorporating wireless remote data transmission. This system would achieve high‐precision object recognition and material classification while supporting autonomous non‐contact operation, effectively addressing challenges in inspecting concealed internal structures while mitigating radiation‐exposure risks. Our work provides a versatile platform for wearable rare‐earth luminescent fabrics and propels radiation protection technology toward prolonged visualization, lightweight architectures, and functional integration. It extends applications beyond medical diagnostics to specialized fields, including underground exploration and nuclear emergency response.

## 2 Results and Discussion

**Figure 1:**
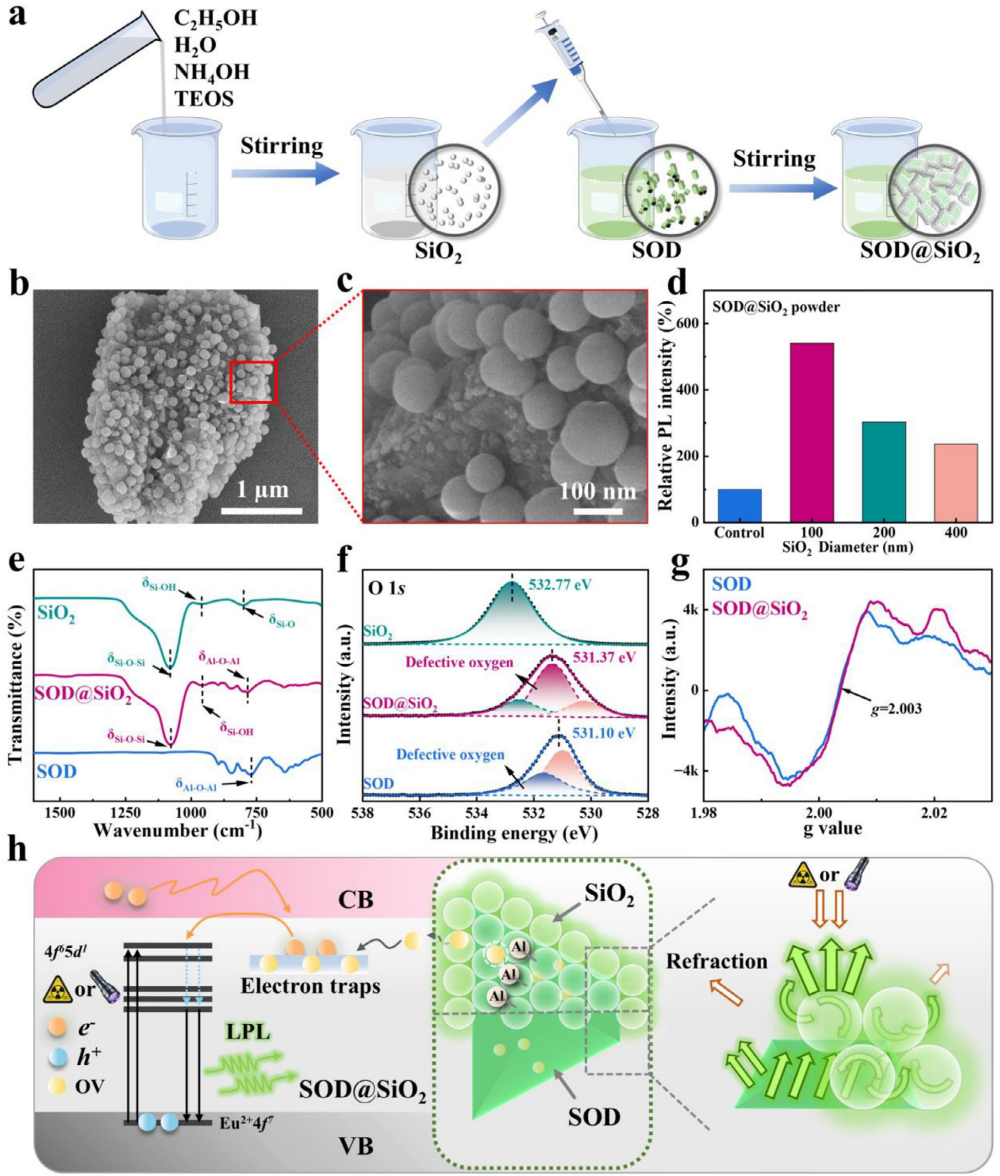
a) Synthesis route for SOD@SiO_2_. b) SEM image of a moth‐eye morphology on SOD@SiO_2_ powder. c) Enlarged view of the moth‐eye‐engineered biomimetic morphology. d) PL spectra of SOD with various SiO_2_ coating (Control refers to SOD powder without SiO_2_ coating). e) FTIR spectra of SOD, SOD@SiO_2_, and SiO_2_. f) Binding energy of O 1*s*. g) EPR signal of OVs. h) Schematic demonstration of luminescence mechanism for SOD and SOD@SiO_2_.

By optimizing the Stöber method,^[^ ^37^ ^]^ we successfully synthesized uniform amorphous SiO_2_ nanospheres (**Figure** **1**a) with tunable diameters ranging from 100 to 800 nm (Figures S1–S5, Supporting Information). Their X‐ray diffraction (XRD) patterns show no distinct diffraction peaks, except for a broad band near 2*θ* at 23.58°, indicative of their amorphous nature (Figure S6, Supporting Information).^[^ ^38^, ^39^ ^]^ During synthesis, surface Si─OH groups act as Brønsted acid sites, which can react with Lewis acid sites on other materials to facilitate the formation of isolated interfacial sites, as observed in systems like SiO_2_‐Al_2_O_3_.^[^ ^40^ ^]^ Taking advantage of Al^3+^ in SOD as a reactive Lewis acid center, the synthesized SiO_2_ nanospheres were chemically coated onto SOD powder in ethanol, yielding SOD@SiO_2_ powder (see Figure 1b,c and the synthesis details see Supporting Information). Compared with pristine SOD powders, SOD@SiO_2_ powder exhibits a markedly enhanced luminescence intensity, with the enhancement becoming more pronounced as the SiO_2_ particle size decreases (Figure 1d). Reducing the SiO_2_ nanosphere diameter to 100 nm enhances luminescence intensity by 402% (Figure 1d; Figure S7a, Supporting Information) and enhances photoluminescence quantum yield (PLQY) from 30.27% (bare SOD) to 35.57% (SOD@100nm‐SiO_2_) (Table S1, Supporting Information) without altering the emission color (Figure S7b, Supporting Information). The XRD patterns of SOD@SiO_2_ and SOD are nearly identical (Figure S8, Supporting Information), indicating that the SiO_2_ coating does not alter the original crystal phase of SOD. Scanning electron microscopy (SEM) and transmission electron microscopy (TEM) images revealed that the SiO_2_ nanospheres were uniformly and regularly coated on the surface of SOD powder (Figure 1b,c; Figure S9, Supporting Information), forming a moth‐eye‐engineered biomimetic shell. Energy‐dispersive X‐ray spectroscopy (EDS) directly confirmed the elemental distributions, verifying SiO_2_ composition in surface nanospheres (Figure S10, Supporting Information). Significantly, SOD@SiO_2_ exhibits reduced particle size versus bare SOD (Figure S11, Supporting Information) a consequence of moth‐eye‐structured SiO_2_ coatings formed during deposition that suppress aggregation through surface‐protective effects.

To elucidate SOD‐SiO_2_ interfacial interactions and luminescence enhancement mechanisms, we conducted Fourier‐transform infrared spectroscopy (FTIR), X‐ray photoelectron spectroscopy (XPS), and electron paramagnetic resonance (EPR) analysis. The FTIR spectra (Figure 1e) reveal a 16 cm^−1^ blueshift (769 → 785 cm^−1^) in Al─O─Al vibrations of SOD@SiO_2_ versus bare SOD.^[^ ^41^ ^]^ Additionally, SOD@SiO_2_ shows distinct peaks at 1076 cm^−1^ (Si─O─Si) and 956 cm^−1^ (Si─OH),^[^ ^42^ ^]^ both redshifted by 4 cm^−1^ compared to pristine SiO_2_. Critically, the Si─O stretching vibration shifts from 800 cm^−1^ (pristine SiO_2_) to 785 cm^−1^ in SOD@SiO_2_, coinciding with the Al─O─Al vibration peak. This spectral overlap provides definitive evidence for SiO_2_‐SOD integration through Al─O─Si bond formation.^[^ ^43^, ^44^ ^]^ Complementary XPS analysis confirms interfacial charge redistribution, with Al 2*p* binding energy increasing by 0.29 eV (Figure S12, Supporting Information) while Si 2*p* decreases by 0.38 eV (Figure S13, Supporting Information). These changes suggest an increase in the electron density around Si and a corresponding decrease around Al in SOD@SiO_2_, likely due to the formation of Al─O─Si bonds. Furthermore, O 1*s* binding energy increases by 0.27 eV versus pristine SOD yet remains 1.40 eV lower than pure SiO_2_ (Figure 1f), indicating an oxygen electronic environment characteristic of asymmetric Al─O─Si bonding configurations. Given the higher electronegativity of Si compared to Al, the bonding oxygen atom tends to shift its electron density toward Si.^[^ ^40^, ^44^, ^45^ ^]^ This leads to a lower electron density around O than in symmetric Al─O─Al bonds in pristine SOD. In contrast, the highly symmetric Si─O─Si network in amorphous SiO_2_ further reduces the electron density around oxygen atoms, accounting for the intermediate O 1*s* binding energy observed in SOD@SiO_2_.^[^ ^46^ ^]^ These findings collectively confirm the chemical bonding between SOD and SiO_2,_ which endows the moth‐eye‐engineered SiO_2_ coating with better robustness than conventional physical dispersions. Non‐linear least squares fitting of the O 1*s* spectra reveals two peaks at 530.94 and 531.66 eV in SOD, attributed to lattice oxygen and defect oxygen, respectively. In SOD@SiO_2_, these peaks shift to 530.23 and 531.35 eV. Notably, the proportion of defect oxygen increases from 39.38% in SOD to 59.17% in SOD@SiO_2_, suggesting that the formation of Al─O─Si bonds promotes the generation of oxygen vacancies (OVs), as reflected by the enhanced defect oxygen signal in the XPS spectra. OVs can trap unpaired electrons, resulting in an EPR signal centered at *g* = 2.003 for both SOD and SOD@SiO_2_ (Figure 1g). Notably, the signal intensity is stronger in SOD@SiO_2_, further confirming the generation of more OVs upon coating.^[^ ^47^, ^48^, ^49^ ^]^

The LPL mechanism of SOD primarily involves the radiative transition of Eu^2+^ from the excited 4*f* ^6^5*d* ^1^ state to the ground 4*f* ^7^ state,^[^ ^50^ ^]^ with Dy^3+^ dopants serving as electron traps for LPL.^[^ ^51^ ^]^ In SOD@SiO_2_, increased OV concentration provides supplementary trap sites, enhancing charge carrier recombination efficiency to extend afterglow duration.^[^ ^48^ ^]^ Concurrently, the ordered assembly of SiO_2_ nanospheres on the SOD surface creates a moth‐eye‐engineered structure that generates a graded RI^[^ ^52^ ^]^ (Figure 1h). The RI of SiO_2_ (*n* ≈ 1.460^[^ ^53^ ^]^) lies between that of air (*n* ≈ 1.000^[^ ^54^ ^]^) and SOD (*n* ≈ 1.685^[^ ^55^ ^]^). As light enters the material from air, the RI gradually transitions from air to the SOD substrate, effectively suppressing interfacial reflection.^[^ ^56^ ^]^ Consequently, SOD@SiO_2_ exhibits lower reflectance in the UV–vis region compared to pristine SOD (Figure S14, Supporting Information), indicating reduced optical loss and enhanced light coupling. This RI gradient may also slow down incident light, increasing its residence time within the material and enhancing absorption.^[^ ^57^ ^]^ Furthermore, Mie scattering within the SiO_2_ nanospheres traps emitted photons, enabling multiple internal reflections and partial re‐excitation of SOD, thus prolonging the LPL^[^ ^58^ ^]^ (Figure S15, Supporting Information). The synergistic outcome is amplified light utilization efficiency and extended carrier residence time. Collectively, the enhanced performance stems from: i) OV‐mediated charge dynamics via Al─O─Si bonding. ii) Photonic engineering through optimized RI progression.

**Figure 2:**
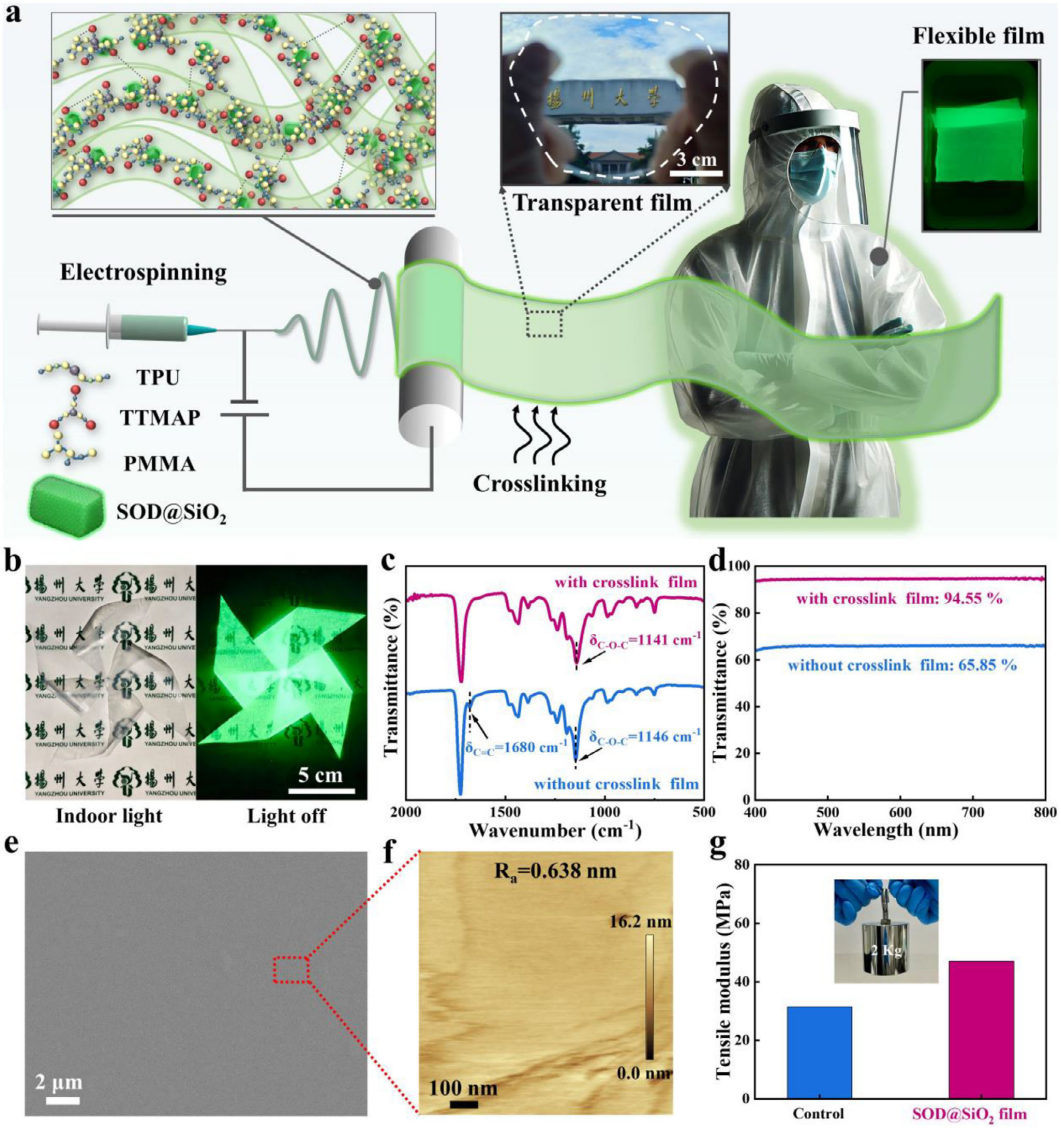
a) Schematic illustration of the fabrication process. b) Performance demonstration of transparent flexible light‐emitting films. c) FTIR of SOD@SiO_2_ films. d) Transmittance evaluation of SOD@SiO_2_ films. e) SEM image of transparent SOD@SiO_2_ films. f) AFM image of SOD@SiO_2_ films. g) Evaluation of tensile modulus and weight‐bearing performance of the SOD@SiO_2_ film.

Following the successful synthesis of small‐sized SOD@SiO_2_ with exceptional LPL, we leveraged electrospinning to integrate these rare‐earth materials into polymer matrices, producing flexible, large‐area luminescent textiles. We systematically evaluate the performance of SOD@SiO_2_ films fabricated with five representative resins. Among them, thermoplastic polyurethane (TPU) and poly(methyl methacrylate) (PMMA) displayed superior emission intensities, as evidenced by the PL spectrum (Figure S16, Supporting Information). While PMMA offers excellent transparency and rigidity, it lacks flexibility; conversely, TPU provides flexibility but exhibits inferior optical properties. Co‐electrospinning these complementary resins effectively integrates their advantages, simultaneously enhancing mechanical flexibility and SOD@SiO_2_ emission intensity. Subsequent thermal treatment induces a glassy‐to‐viscoelastic transition, enabling plastic flow and structural reorganization that yields dense, uniform semi‐transparent films (Figure S17, Supporting Information). Interestingly, incorporating a trifunctional aziridine crosslinker (TTMAP) promotes inter‐fiber crosslinking, further enhancing the optical transparency of the resulting film (Figure S18, Supporting Information). Hence, we prepare the electrospinning ink by dispersing SOD@SiO_2_, resin, and crosslinker in a binary solvent of N, N’‐dimethylformamide (DMF). Following electrospinning and subsequent thermal crosslinking, we fabricated large‐area (13 × 17 cm^2^) transparent flexible LPL films within 1 h (**Figure** **2**a; Figure S19, Supporting Information) (Electrospinning conditions: applied voltage 16 kV, collector speed 300 rpm, tip‐to‐collector distance 10 cm, solution flow rate 4 mL h^−1^. The details are in Materials synthesis of Supporting Information). The film combines tunable rigidity‐flexibility balance, enabling complex shape processing while maintaining mechanical integrity and luminescent performance (Figure 2b). We systematically quantified the luminescence and structural properties of electrospun films across varied SOD@SiO_2_ loadings (3–25 wt.%, Figure S20, Supporting Information). Emission intensity peaked at 15 wt.%, beyond which phase separation, fiber disruption, and filler leakage occurred (Figure S21, Supporting Information). This confirms that excessive additive content compromises fiber integrity while inducing material loss. We thus established 15 wt.% as the optimal loading for balanced performance and processing stability.

We conducted the FTIR spectra characterization, and we found that after co‐electrospinning with TTMAP, the C═C peak (1680 cm^−1^) from PMMA disappeared in the SOD@SiO_2_ films, indicating a radical addition reaction between TTMAP and PMMA monomers (Figure 2c). Concurrently, C─O─C stretching vibrations shift from 1146 to 1141 cm^−1^ in PMMA and from 1137 to 1142 cm^−1^ in TPU (Figure S22, Supporting Information), confirming TTMAP‐mediated crosslinking between polymer matrices. In the uncrosslinked state, the SOD@SiO_2_ film contains numerous inter‐fiber voids, causing multiple scattering and reflection of light at the interfaces, which increases the reflectance and reduces transmittance. TTMAP acts as a molecular bridge within the composite, effectively linking the PMMA and TPU polymer chains. This molecular bridging accelerates inter‐fiber pore filling,^[^ ^59^ ^]^ enhancing film transparency^[^ ^60^, ^61^ ^]^ to achieve 94.55% average visible transmittance (400–800 nm) – a 43.5% increase over non‐crosslinked films (65.85%) (Figure 2d). The addition of TTMAP also contributed to a more uniform size distribution and dispersion of SOD@SiO_2_ powder (Figure S23, Supporting Information), likely due to improved resin flow during the viscoelastic state, which helped suppress particle aggregation. Comparison of the morphological evolution reveals that the pristine SOD@SiO_2_ electrospun film consists of an interconnected network of fibers ≈2 µm in diameter (Figure S24, Supporting Information). After the heat treatment for crosslinking, the fibrous network transforms into a smooth surface (Figure S25, Supporting Information), whereas the addition of TTMAP promotes a more complete crosslinking reaction, resulting in more thorough filling of the inter‐fiber voids (Figure 2e; Figure S26, Supporting Information). This improvement is attributed to improved homogeneity in resin flow enabled by TTMAP, which mitigates defects arising from non‐uniform polymer dynamics or localized overheating during thermal treatment, thereby promoting the uniformity of the film. Atom force microscope (AFM) analysis confirms exceptional surface smoothness (R_a_ = 0.638 nm, Figure 2f), which directly enables high optical transparency by minimizing light‐scattering losses. This aligns with the measured 94.55% visible transmittance (Figure 2d). Critically, transparency in composite films critically depends on minimizing RI mismatch and particle‐induced scattering.^[^ ^26^ ^]^ Our SiO_2_ nanosphere incorporation engineered SOD@SiO_2_’s RI to 1.505 – within 0.47% of TPU (*n* ≈ 1.498^[^ ^62^ ^]^) and 0.87% of PMMA (*n* ≈ 1.492^[^ ^63^ ^]^)–effectively eliminating interface scattering through precision optical matching.^[^ ^64^ ^]^ Concurrently, mechanical optimization addresses the essential flexibility‐robustness balance for wearable systems.^[^ ^65^ ^]^ The fatigue resistance of the SOD@SiO_2_ film was assessed through 1000 cyclic stretching–releasing tests at 30% strain (Figure S27a, Supporting Information). During the first cycle, the film exhibited a strength of 4.1 MPa and a dissipated energy of 60.1 kJ·m^−3^ (Figure S27b, Supporting Information). Both parameters decreased with continued cycling and stabilized after ≈100 cycles. The initially high stress and energy dissipation can be attributed to irreversible microstructural rearrangements and minor damage during the early loading stage, after which the mechanical response entered a quasi‐steady regime.^[^ ^66^ ^]^ This cycling‐induced stabilization is advantageous for wearable devices, as it demonstrates that the films maintain stable mechanical performance and energy dissipation under long‐term repeated bending and stretching, thereby meeting the durability and reliability requirements of flexible applications.^[^ ^67^ ^]^ The SOD@SiO_2_ films achieve a 47.03 MPa elastic modulus (49.02% increase vs polymer control) while withstanding up to 2 kg of loads (Figure 2g). The high modulus and strength are primarily attributed to the incorporation of SOD and its synergistic effect with SiO_2_ (Figure S28, Supporting Information). And the XRD patterns confirm that the diffraction peaks of SOD@SiO_2_ films match those of the standard reference (Figure S29, Supporting Information), indicating that the crystalline phase of SOD remains unchanged after electrospinning. This dual optical‐mechanical engineering enables scalable production at $0.0033 per cm^2^ (Table S2, Supporting Information), establishing an industrially viable platform where high‐performance sustainability aligns with practical industrial deployment.

**Figure 3:**
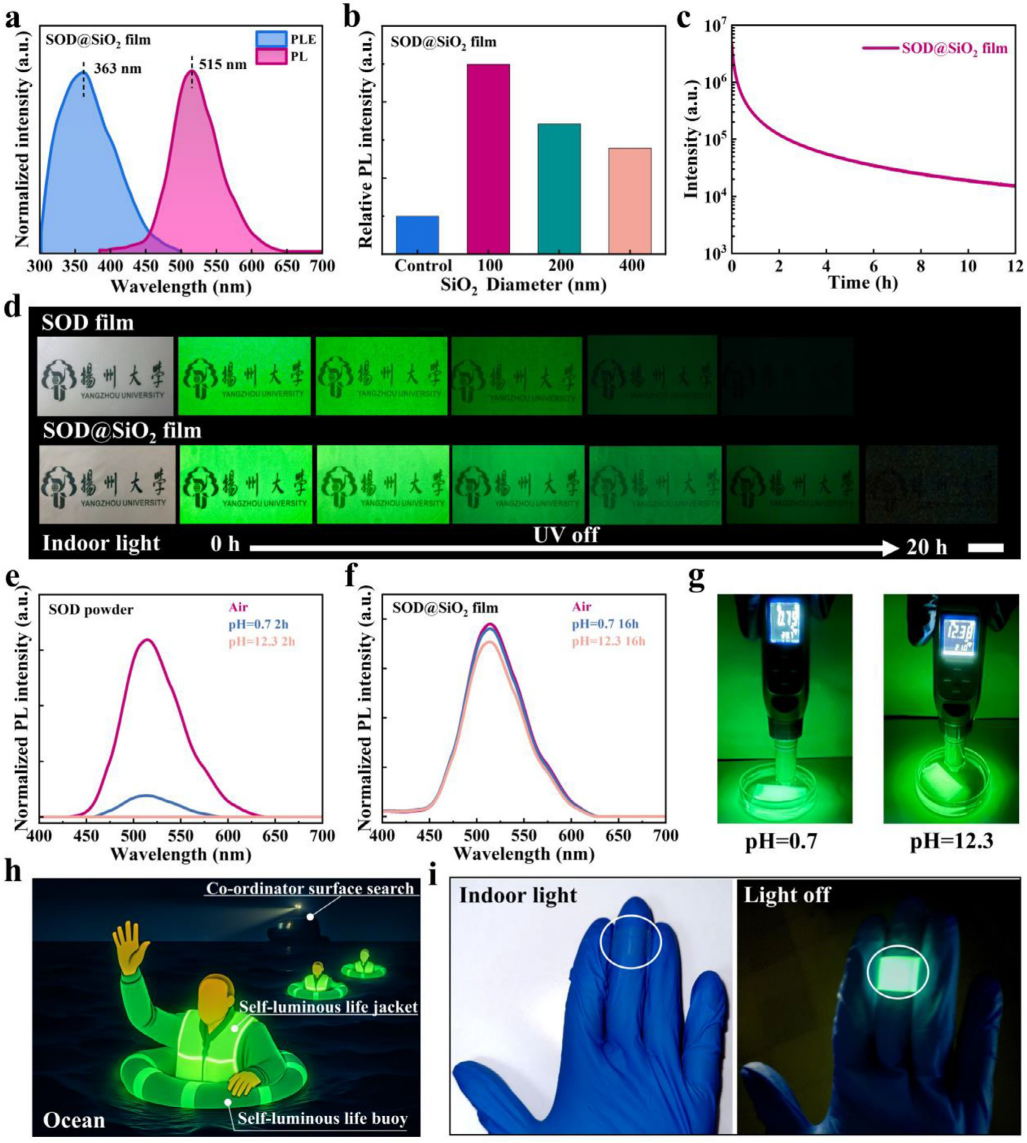
a) PL and PLE spectra of SOD@SiO_2_ film. b) Relative PL intensity for SOD@SiO_2_ films with varying particle sizes of SiO_2_. c) PL kinetic spectrum of SOD@SiO_2_ films after 365 nm UV light excitation for 10 s. d) Photographs of the LPL performances for the films after 10 s excitation of 365 nm UV light. e) PL spectra of SOD powder deposited in neutral/acid/alkaline solutions. f) PL spectra of SOD@SiO_2_ films deposited in acid/neutral/alkaline solutions. g) Images of SOD@SiO_2_ films immersion in acid or alkaline solutions for 16 h. h) Schematic illustration of self‐luminous SOD@SiO_2_ films sewn on a life rescue in the ocean night‐time. i) A wearable transparent SOD@SiO_2_ ring for LPL‐based visual signaling.

The luminescent performance of SOD@SiO_2_ film was systematically investigated through photophysical characterization. Controlled experiments with SiO_2_‐only films show no detectable PL, ruling out the possibility that the PL enhancement is partially attributed to the polymer‐SiO_2_ interface (Figure S30, Supporting Information). SOD@SiO_2_ films maintain chromaticity coordinates identical to pristine SOD (Figure S31, Supporting Information), exhibiting an emission band centered at 515 nm (**Figure** **3**a). Their photoluminescent excitation (PLE) peak at 363 nm yielded g a large Stokes shift (152 nm), while solid‐state UV–vis spectra confirm exclusive absorption below 456 nm with negligible self‐absorption (Figure S32, Supporting Information), which is a critical feature enabling LPL display. Remarkably, it demonstrates a 80% quantum yield enhancement versus bare SOD (PLQY: 54.50% vs 30.27%, Table S1, Supporting Information), with optimized ≈100 nm SiO_2_ nanospheres yielding quadrupled PL intensity (Figure 3b). This amplification stems from minimized scattering losses, as evidenced by reduced diffuse reflectance following crosslinking and SiO_2_ coating (Figures S33–S35, Supporting Information), thereby enhancing photon harvesting efficiency. Furthermore, SiO_2_ confinement dramatically enhances the LPL performance of SOD@SiO_2_ film (Figure S36, Supporting Information), extending the TRPL lifetime with significantly retarded decay kinetics (Figure 3c). This prolonged emission enables clear visualization of the “Yangzhou University” logo through the transparent film for 20 h post‐excitation (Figure 3d), demonstrating exceptional excitation storage capacity.

The environmental stability of SOD@SiO_2_ films was systematically evaluated across multiple stress conditions, demonstrating exceptional robustness through three key stability metrics. Under ambient storage, these films retained 84.4% of their initial PL intensity after 720 h. Based on fitting calculations, PL_T__50_ was estimated to be 2216 h (Figure S37a,b, Supporting Information). Accelerated UV aging tests (365 nm, 84 h) revealed superior photostability with merely 9.2% intensity loss (Figure S38, Supporting Information). Remarkably, the SOD@SiO_2_ film exhibited unprecedented luminescence stability in extreme aqueous environments. As shown in Figure 3e, the pristine SOD powder suffered complete luminescence loss (100%) in pH 12.3 and 88.4% degradation in pH 0.7 within 2 h (Figure 3e). But the SOD@SiO_2_ film maintained > 91% intensity after 16‐h immersion (Figure 3f,g), with persistent luminescence detectable for 18 h (Figure S39, Supporting Information). This exceptional stability originates from resin encapsulation that transforms the hydrophilic SOD@SiO_2_ powder (10° contact angle) into hydrophobic films (135°), creating an effective barrier against environmental degradation (Figure S40a,b, Supporting Information). The exceptional stability and luminescent performance of SOD@SiO_2_ films translate directly into practical applications, as demonstrated through multiple proof‐of‐concept implementations.

Their unique combination of long‐term ambient stability, UV resistance, and 18‐h persistent luminescence in aquatic environments establishes them as a breakthrough material for emergency systems, exemplified by prototype life‐saving rings and life jackets that maintain bright visibility under harsh conditions (Figure 3h,i; Figure S41, Supporting Information). Beyond safety applications, the SOD@SiO_2_ films enable novel optical technologies – laser‐engraved luminescent QR codes retain high‐resolution information storage with > 10‐h autonomous visibility, creating opportunities for low‐energy smart displays. (Figure S42, Supporting Information). The material's solar‐activated persistent emission, environmental robustness, and large‐area processability outperform conventional phosphorescent materials across three key metrics: operational lifetime (2216 h T_50_), extreme condition stability (< 10% degradation in pH 0.7–12.3), and flexible substrate compatibility, positioning SOD@SiO_2_ films as a versatile platform for next‐generation luminescent devices.

**Figure 4:**
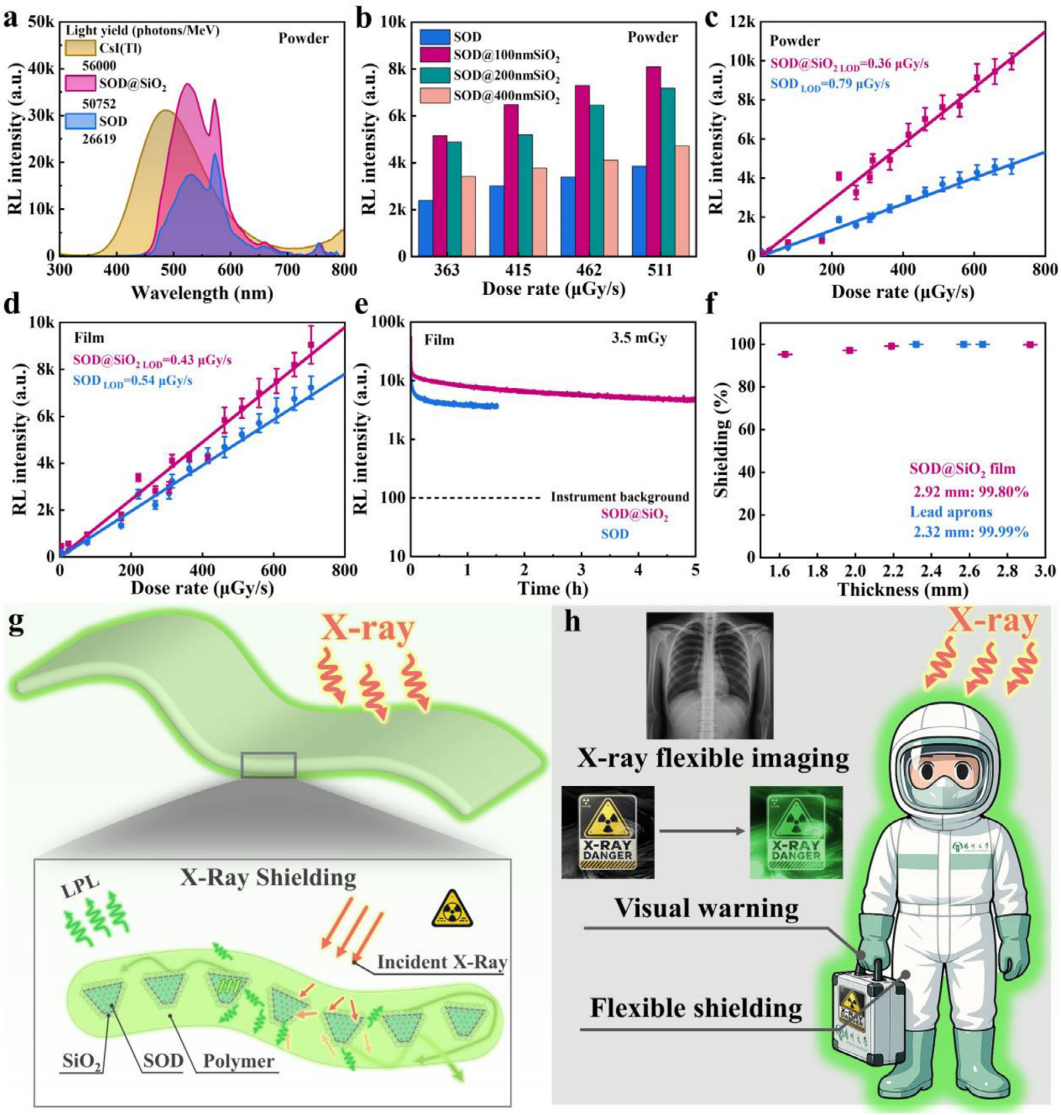
a) RL spectra and LY of SOD and SOD@SiO_2_ powder. Sample thickness: 1 mm (same as CsI(Tl) reference). b) RL intensity of SOD powder with/without SiO_2_ coating under various X‐ray doses. c) Linear X‐ray dose response fitting of SOD and SOD@SiO_2_ powder. d) Linear X‐ray dose response fitting of SOD and SOD@SiO_2_ films. e) LPL decay profiles for SOD and SOD@SiO_2_ films after 10 s X‐ray exposure (10 kV, 15 µA). f) X‐ray shielding efficiency of SOD@SiO_2_ films in comparison to commercial lead aprons. g) Schematic diagram of SOD@SiO_2_ film X‐ray shielding mechanism. h) Schematic illustration of SOD@SiO_2_ films’ applications in X‐ray shielding, warning, and radiation visualization.

Encouragingly, the moth‐eye‐engineered SOD@SiO_2_ powder exhibit significantly enhanced RL intensity compared to pristine SOD powders (**Figure** **4**a), achieving a light yield (LY) of 50 752 photons MeV^−1^. This value represents a 90.66% enhancement compared to uncoated SOD, and is comparable to that of commercial CsI(Tl) scintillators. Controlled experiments confirmed that the RL spectra of SiO_2_‐only films exhibited no detectable luminescence. (Figure S43, Supporting Information). In addition, the RL spectral analysis reveals a distinct emission peak at 572 nm, which can be attributed to the 4*f*‐4*f* transition of Dy^3+^ ions in the host lattice, activated under high‐energy X‐ray excitation. This activation mechanism is unique to the RL process, as the high‐energy carriers can induce *f*‐*f* transitions that are typically forbidden under lower‐energy photoluminescence excitation.^[^ ^68^ ^]^ However, the dominant RL mechanism, analogous to PL, is primarily attributed to the allowed 5d‐4f transition of Eu^2^⁺ (Figure 1g).^[^ ^50^, ^68^ ^]^ The dual strategy of SiO_2_ coating and electrospinning synergistically enhances RL intensity (Figure S44, Supporting Information), as evidenced by the consistent RL enhancement across varying X‐ray dose rates (Figure 4b; Figure S45, Supporting Information). Notably, both SOD and SOD@SiO_2_ demonstrate linear RL response to X‐ray dose rates (Figure 4c,d), with SiO_2_ coating substantially improving detection sensitivity. The LODs of X‐ray dose rates decrease from 0.79 µGy s^−1^ (SOD powder) to 0.36 µGy s^−1^ (SOD@SiO_2_ powder) and from 0.54 µGy s^−1^ (SOD film) to 0.43 µGy s^−1^ (SOD@SiO_2_ film), all surpassing the clinical diagnostic threshold (5.5 µGy s^−1^, Table S3, Supporting Information).^[^ ^69^ ^]^ Analogous to the UV‐excited LPL behavior, SOD@SiO_2_ film exhibited bright LPL following 10 s of X‐ray irradiation, with a longer LPL duration than that of the uncoated SOD films (Figure 4e).

Beyond detection, owing to its flexibility, transparency, and scalability, the SOD@SiO_2_ film offers promising potential as a curved, low‐dose X‐ray delayed imaging screen. Considering the high atomic number elements in SOD (such as Sr, Dy, and Eu), the X‐ray shielding capability of the flexible film was also evaluated. Remarkably, a 2.92mm‐thick SOD@SiO_2_ film achieved a shielding efficiency of 99.80%, comparable to commercial lead aprons (2.32 mm, 0.35 mm Pb equivalence, Figure 4f). Such X‐ray shielding performances can not be achieved with SiO_2_‐only film (Figure S46, Supporting Information). This dual functionality arises from efficient X‐ray absorption by uniformly distributed SOD@SiO_2_ powder, which simultaneously blocks radiation while converting absorbed energy into green LPL for visual exposure monitoring (Figure 4g,h).

Unlike conventional lead‐based shielding materials that are rigid, heavy, and toxic, these films offer lightweight flexibility (< 0.5 mm bend radius) and environmental safety while maintaining 0.43 µGy s^−1^ leakage detection sensitivity. The multifunctional SOD@SiO_2_ film, which integrates effective radiation shielding, luminescent warning capabilities, and X‐ray visualization, positions this material system as a transformative platform for next‐generation smart protective gear that overcomes the fundamental limitations of conventional radiation protection technologies.

**Figure 5:**
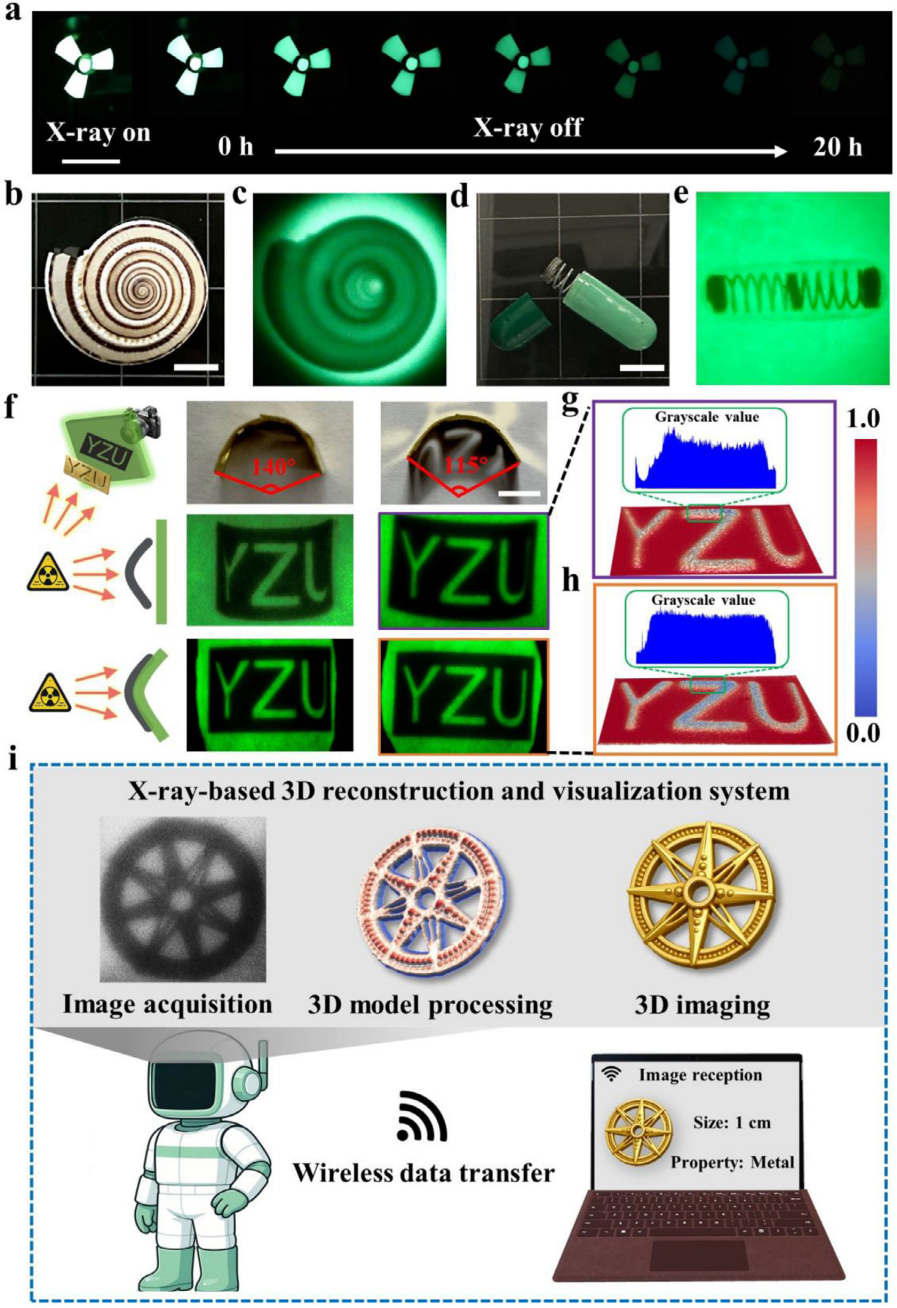
a) LPL visual alert triggered SOD@SiO_2_ films after X‐ray exposure for 10 s (50 kV, 200 µA). b)‐c) Images of a shell under b) natural light and c) X‐ray (50 kV, 200 µA, scale bar: 0.5 mm, film thickness: 0.1 mm). d)‐e) Images of an encapsulated metal spring under (d) natural light and (e) X‐ray (50 kV, 200 µA, scale bar: 0.5 mm, film thickness: 0.1 mm). f) Demonstration of a flexible scintillator screen and the corresponding X‐ray images (scale bar: 1 cm, film thickness: 0.1 mm). g) The grayscale image of the planar X‐ray imaging. h) The grayscale image of the curved surface X‐ray imaging. i) X‐ray‐based 3D reconstruction and visualization system.

In radiation‐prone environments, persistent visual warnings are critical for safety monitoring, a challenge addressed by the SOD@SiO_2_ film's exceptional long‐term radioluminescence. Upon X‐ray exposure, the film emits vivid green afterglow lasting 20 h (**Figure** **5**a), providing reliable hazard alerts without power requirements, particularly vital in emergency scenarios. Beyond warnings, the film's high‐intensity RL luminescence (Figure S47, Supporting Information) and flexibility enable low‐dose, high‐resolution imaging of curved objects, successfully resolving internal structures in complex specimens like seashells and spring‐loaded capsules (Figure 5b–e). When evaluated using a standard X‐ray resolution phantom, the film achieves an imaging resolution of up to 9.6 lp mm^−1^ (Figure S48, Supporting Information), surpassing the resolution requirement for portable X‐ray chest imaging systems (3.0 lp mm^−1^).^[^ ^70^ ^]^ Due to its exceptionally long‐lasting RL property, the film functions effectively as an X‐ray imaging screen with delayed imaging capability. Remarkably, these films preserve high‐resolution radiographic images for up to 4 h after X‐ray source removal (Figure S49, Supporting Information), a critical advancement for time‐sensitive diagnostic applications. This capability addresses a fundamental limitation of conventional rigid scintillator screens, which suffer from geometric distortion and vignetting when imaging curved surfaces due to uneven X‐ray absorption.^[^ ^71^, ^72^ ^]^ As a flexible scintillation screen, the SOD@SiO_2_ film adapts precisely to complex contours, eliminating the progressive blurring and structural incompleteness observed in planar imaging (e.g., distorted letters “Z” and “U” in Figure 5f). Subsequently, both conventional planar X‐ray imaging and surface‐conformal imaging at a 115° bend were subjected to grayscale processing by our self‐developed 3D image modeling software (Figure 5g,h, the software details described in Supporting Information). In the planar projection method, the grayscale distributions of the letters “Z” and “U” are non‐uniform. In contrast, surface‐conformal X‐ray imaging, enabled by the flexibility of the SOD@SiO_2_ film, allows the letter “U” to be rendered completely and uniformly.

Moreover, the letter “Z” maintains a consistent grayscale distribution despite the curvature, demonstrating that the flexible SOD@SiO_2_ film effectively mitigates vignetting effects in non‐planar X‐ray imaging. When combined with the film's high‐resolution delayed imaging capability and excellent flexibility, this approach offers promising prospects for next‐generation wearable radiation imaging systems that transcend the limitations of rigid scintillators.

Current X‐ray imaging systems predominantly require on‐site operator involvement, exposing personnel to potential radiation hazards. To address this critical limitation, we propose an X‐ray‐based 3D image reconstruction and visualization system (Figure 5i). This innovative system combines three critical technological advancements: high‐sensitivity SOD@SiO_2_ film detectors enabling low‐dose imaging, 3D reconstruction algorithms for voxel‐based modeling with material‐specific gradient preservation, and secure wireless data transmission. When deployed with an X‐ray imaging robot, this system autonomously acquires structural data from concealed objects and reconstructs their internal geometries through calibrated grayscale‐intensity correlations, achieving simultaneous boundary delineation and material characterization. The architecture's closed‐loop operation, featuring radiation‐zone data acquisition with remote processing, suggests great potential for automated nondestructive testing and diagnosis in hazardous environments.

## 3 Conclusion

In this work, we demonstrate that SOD@SiO_2_ luminescent composites with carefully engineered moth‐eye morphology can overcome critical limitations in radiation detection materials. By forming Al─O─Si covalent bonds between SiO_2_ nanospheres and the SOD powder, followed by scalable electrospinning, we achieved large‐area flexible films that combine exceptional optical performance, environmental resistance, and multifunctionality. The optimized films exhibit remarkable properties: 99.80% X‐ray shielding (equivalent to 0.35 mm lead), persistent radiation alerts lasting 20 h, high‐resolution (9.6 lp mm^−1^) imaging on curved surfaces, and sensitive detection at 0.43 µGy s^−1^. These capabilities open new possibilities for practical applications ranging from self‐powered emergency lighting to smart protective textiles and automated 3D X‐ray imaging systems. Our approach successfully balances the traditionally competing demands of performance, flexibility, and stability through three key innovations: bioinspired photonic design, scalable membrane fabrication, and practical engineering solutions. This work provides a foundation for developing next‐generation protective technologies across medical, industrial, and defense sectors.

## Conflict of Interest

The authors declare no conflict of interest.

## Supporting information



Supporting Information

## Data Availability

The data that support the findings of this study are available in the supplementary material of this article.
