# Supplementary material for: Moth‐Eye‐Engineered Flexible Films for X‐Ray Shielding and Persistent Radiation Warning
Source: Adv Sci (Weinh). 2025 Oct 13;12(48):e14035. doi: 10.1002/advs.202514035 (PMC12752565; doi:10.1002/advs.202514035)
Supplement: Supplementary file 1 — Supporting Information [file ADVS-12-e14035-s001.docx]

Supplementary Information

**Moth-Eye-Engineered Flexible Films for X-Ray Shielding and Persistent** **Radiation Warning**

Yuansheng Jiang^#^, Wen-Guang Li^#^, Xiuji Yi, Meifang Yang, Xinyi Lin, Yaxun Hu, Yicheng Yuan, Qiang Ma, Yuping Li, Fengyun Wang, Qin Xu, Wenjing Zhang, Yu-Xin Chen*, Tian Tian*, Huan Pang*

**Content**

[**1. Experimental section** 5](#_Toc210761098)

[**1.1 Chemical reagent** 5](#_Toc210761099)

[**1.2 Instrumental** 5](#_Toc210761100)

[**1.3 Synthesis** 6](#_Toc210761101)

[**1.4 Characterization of X-ray Excited Luminescence** 7](#_Toc210761102)

[**1.5 Afterglow photo shooting parameters and 3D imaging software** 9](#_Toc210761103)

[**Figure S1.** Control of SiO_2_ particle size by tuning water content during synthesis. 10](#_Toc210761104)

[**Figure S2.** Control of SiO_2_ particle size by tuning the content of ammounium hydroxide during synthesis. 11](#_Toc210761105)

[**Figure S3.** Control of SiO_2_ particle size by tuning the reaction time. 12](#_Toc210761106)

[**Figure S4.** Control of SiO_2_ particle size by tuning TEOS content during synthesis. 13](#_Toc210761107)

[**Figure S5.** Control of SiO_2_ particle size by tuning the reaction temperature. 14](#_Toc210761108)

[**Figure S6.** XRD patterns of SiO_2_ nanospheres with different particle sizes. 15](#_Toc210761109)

[**Figure S7.** a) PL spectra and b) CIE coordinates of SOD@SiO_2_ powder prepared with different SiO_2_ particle sizes. 16](#_Toc210761110)

[**Table S1.** The table of PLQY parameters of SOD film and SOD@SiO_2_ film. 17](#_Toc210761111)

[**Figure S8.** XRD patterns of SOD and SOD@SiO_2_ powder coating different sizes SiO_2_ particle. 18](#_Toc210761112)

[**Figure S9.** The TEM image of SOD@SiO_2_ powder. 19](#_Toc210761113)

[**Figure S10.** The EDS mapping of a) SOD and b) SOD@SiO_2_ powder. 20](#_Toc210761114)

[**Figure S11.** The SEM images of a) SOD and b) SOD@SiO_2_ powder. 21](#_Toc210761115)

[**Figure S12.** The Al 2*p* XPS spectra of SOD with or without SiO_2_ coating. 22](#_Toc210761116)

[**Figure S13.** The Si 2*p* XPS spectra of SOD with or without SiO_2_ coating. 23](#_Toc210761117)

[**Figure S14.** Diffuse reflectance spectra of SOD and SOD@SiO_2_ powder. 24](#_Toc210761118)

[**Figure S15.** The afterglow images of SOD and SOD@SiO_2_. 25](#_Toc210761119)

[**Figure S16.** PL spectra of SOD@SiO_2_ in various matrices. 26](#_Toc210761120)

[**Figure S17.** The temperature-dependent transmittance of films without crosslinking agent TTMAP. (a) 298 K, (b) 373 K, (c) 393 K, (d) 433 K. (e) Transmittance variation as a function of treatment temperature. 27](#_Toc210761121)

[**Figure S18.** Temperature-dependent optical transmittance of TTMAP-crosslinked polymer films. (a) 298 K, (b) 373 K, (c) 393 K, (d) 433 K. (e) Transmittance vs. temperature profile demonstrating enhanced thermal stability up to 433 K. 28](#_Toc210761122)

[**Figure S19.** A large-area transparent SOD@SiO_2_ film. 29](#_Toc210761123)

[**Figure S20.** SEM images of films with different SOD@SiO_2_ mass contents. 30](#_Toc210761124)

[**Figure S21.** PL spectra of films with different SOD@SiO_2_ additive amounts. 31](#_Toc210761125)

[**Figure S22.** The FTIR spectra of TPU-TTMAP films and TPU films. 32](#_Toc210761126)

[**Figure S23.** Fluorescence images of SOD@SiO_2_ films with and without TTMAP. 33](#_Toc210761127)

[**Figure S24.** SEM image of SOD@SiO_2_ film before heat treatment. 34](#_Toc210761128)

[**Figure S25.** SEM images of non-crosslinked films under thermal treatment (without TTMAP). 35](#_Toc210761129)

[**Figure S26.** SEM images of crosslinked films under thermal treatment (with TTMAP). 36](#_Toc210761130)

[**Figure S27**. Mechanical properties of the SOD@SiO_2_ film. a) Fatigue resistance of SOD@SiO_2_ film with 1000 successive loading–unloading cycles under a constant strain of 30%. b) Summarized maximum stress and dissipated energy of SOD@SiO_2_ film during the cyclic stretching–releasing process. 37](#_Toc210761131)

[**Figure S28.** The stress-strain curves of SiO_2_, SOD@SiO_2_ and blank films. (The blank film is composed exclusively of resin.) 38](#_Toc210761132)

[**Figure S29.** XRD patterns of SOD and SOD@SiO_2_ films. 39](#_Toc210761133)

[**Table S2.** Cost evaluation of SOD@SiO_2_ film. 40](#_Toc210761134)

[**Figure S30.** The PL spectrum of SiO_2_ film. *λ_ex_ = 365 nm* 41](#_Toc210761135)

[**Figure S31.** The PL spectra and CIE coordinates of SOD and SOD@SiO_2_ films. 42](#_Toc210761136)

[**Figure S32.** The UV-Vis absorbance spectra of SOD and SOD@SiO_2_ films. 43](#_Toc210761137)

[**Figure S33.** Diffuse reflectance spectroscopy of the corresponding film. 44](#_Toc210761138)

[**Figure S34.** Diffuse reflectance spectroscopy of SOD@SiO_2_ powders and SOD@SiO_2_ film. 45](#_Toc210761139)

[**Figure S35.** PL spectra of SOD@SiO_2_ films with varying optical transmittance. *λ_ex_ = 365 nm* 46](#_Toc210761140)

[**Figure S36.** The afterglow spectrum of the SOD and SOD@SiO_2_ film. *λ_ex_ = 365 nm* 47](#_Toc210761141)

[**Figure S37.** PL intensity tracking of SOD@SiO_2_ films under ambient storage. a) The PL spectra of SOD@SiO_2_ films at different aging times b) *T*_PL50_ was calculated by fitting the SOD@SiO_2_ film stored at room temperature. 48](#_Toc210761142)

[**Figure S38.** Evaluation of the as-prepared SOD@SiO_2_ films under continuous 365nm irradiation. 49](#_Toc210761143)

[**Figure S39.** The afterglow performance of SOD@SiO_2_ films in strong acidic solution (pH=0.79) and strong alkaline solution(pH=12.32). 50](#_Toc210761144)

[**Figure S40.** Contact angle analysis. a) SOD@SiO_2_ powder and b) SOD@SiO_2_ film. 51](#_Toc210761145)

[**Figure S41.** Demonstration of transparent and large-area SOD@SiO_2_ film as a component of protective clothing with long afterglow. 52](#_Toc210761146)

[**Figure S42.** Demonstration of SOD@SiO_2_ film application. A QR code printed on a SOD@SiO_2_ film for solar-energy storage and LPL releasing. 53](#_Toc210761147)

[**Figure S43.** The RL spectra of SiO_2_ film and SOD@SiO_2_ film. 54](#_Toc210761148)

[**Figure S44.** RL spectra of SOD powder, SOD film and SOD@SiO_2_ film. 55](#_Toc210761149)

[**Figure S45.** RL spectra of SOD and SOD@SiO_2_ powder under various X-ray dose rate. a) SOD; b) SOD@100nmSiO_2_; c) SOD@200nmSiO_2_; d) SOD@400nmSiO_2_. 56](#_Toc210761150)

[**Table S3.** Summary of detection limits. 57](#_Toc210761151)

[**Figure S46.** X-ray shielding efficiency of SiO_2_ film. 58](#_Toc210761152)

[**Figure S47.** RL spectra of SOD@SiO_2_ film. Sample thickness: 1 mm (same as CsI(Tl) reference). 59](#_Toc210761153)

[**Figure S48.** Images of a resolution phantom under a) natural light and b) X-ray (50 kV, 200 μA, scale bar: 4 mm). c) Modulation transfer function (MTF) of the imaging panel as a function of spatial frequency. 60](#_Toc210761154)

[**Figure S49.** Demonstrate the time-lapse imaging function of the transparent and large-area SOD@SiO_2_ film, excited by 30 W, 60 kV X-ray light. 61](#_Toc210761155)

**1. Experimental section**

**1.1 Chemical reagent**

The commercial SrAl_2_O_4_:Eu^2+^, Dy^3+^ (SOD) green-emitted powder was purchased from Shenzhen looking long technology co., Ltd. Thermoplastic urethane (TPU) was purchased from Qingdao Nuokang Environmental Protection Technology Co., Ltd. Polymethyl methacrylate (PMMA) was purchased from Dongguan Shunjie Plastic Technology Co., Ltd. The N, N-dimethylformamide (DMF), ethyl alcohol (EtOH), ammonia water (NH_4_OH), tetraethylorthosilicate (TEOS) and acetone was purchased from Sinopharm Shanghai Chemical Reagent Company. Trimethylolpropane tris (2-methyl-1-aziridinepropionate) (TTMAP) was purchased from Aladdin Reagent (China). All reagents were employed as obtained without additional purification.

**1.2 Instrumental**

The Fourier transform infrared spectra were characterized by using Agilent Cary 610/670 micro-infrared spectrometer. A Bruker A300-10/12 paramagnetic resonance spectroscopy instrument (EPR) was employed to characterize the oxygen vacancies. The Agilent Cary 5000 ultraviolet-visible near-infrared absorption spectrometer was used to characterize the absorption, reflection and transmission properties of the film and powder samples. The elastic modulus of the film was characterized by using the MTS WCMT6103 electronic universal testing machine. The morphological characteristics of all samples were characterized by using a Zeiss Supra 55 field emission scanning electron microscope (SEM). The surface roughness of the film was characterized by using the Shimadzu SPM-9700HT atomic force microscope (AFM). The microscopic coating structure of the sample was observed by using a Hitachi HT7800 transmission electron microscope (TEM). The dark-field fluorescence images of the thin films were characterized by an OLYMPUS DO-BX53 fluorescence microscope (DO-BX53). The steady-state photoluminescence (PL) and time-resolved photoluminescence lifetime (TRPL) measurements of the samples were conducted using an Edinburgh FLS1000 fluorescence spectrometer. Photoluminescence quantum yield (PLQY) was measured using an integrating sphere on the FLS1000 fluorescence spectrometer, with strict consistency maintained between samples. A Bruker D8 Advance X-ray diffractometer was used to measure the XRD spectra of the samples. The 2*θ* range was 5° to 80°, with a step rate of 20 s and a step size of 0.02°. X-ray photoelectron spectroscopy (XPS) was used to investigate the chemical identity of the samples by a Thermo Fisher ESCALAB 250Xi spectrometer equipped with a standard monochromatic Al-Kα source.

**1.3 Synthesis**

**Synthesis of SiO_2_ of Different Sizes**

Firstly, 0.5 mL of H_2_O and 0.25 mL of ammonia solution were added into 5 mL of ethanol solution and stirred for 10 minutes to prepare a mixed solution. Subsequently, 1.5 mL of TEOS was added to the above mixed solution and stirred continuously at 25 ℃ for 4 hours to ensure complete reaction. The obtained SiO_2_ nanospheres have a particle diameter of 100 nm. By controlling the variables and changing the proportions of H_2_O, ammonia solution, and TEOS in the reaction conditions, as well as the reaction temperature and time, different-sized SiO_2_ nanospheres could be obtained.

**Synthesis of SOD@SiO_2_**

Firstly, a dispersion was prepared by stirring 0.187 g SOD in 10 mL of ethanol for 20 minutes at room temperature. Then, 0.02 mL of SiO_2_ nanosphere dispersion was added to the above SrAl_2_O_4_:Eu^2+^,Dy^3+^ dispersion and stirred at 25 ℃ for 2 hours to ensure complete reaction. Subsequently, filtration and centrifugation were performed to collect SOD@SiO_2_ (centrifugation parameters: 11000 rpm, 10 minutes). After centrifugation, the sample was placed in a 60 ℃ oven to dry completely.

**Fabrication of large-area flexible transparent films (13×17cm)**

First, 0.66 g of TPU was dissolved in a mixed solvent of 1.37 mL DMF with 1.32 mL acetone to prepare a 22 wt% solution. A 30 wt% PMMA solution was obtained by dissolving 0.3g PMMA in 0.74mL of DMF. After mixing the above solutions, 0.1 g TTMAP was added. The resulted solution was stirred for 30 minutes to ensure complete mixing. Subsequently, 0.187 g SOD@SiO_2_ powder was added to the above solution and stirred thoroughly to prepare the electrospinning ink. The components can be proportionally enlarged to expand production. Pour the ink into a 10 mL syringe equipped with a metal needle (inner diameter 0.86 mm). The syringe was placed horizontally on the injection pump, connected to the positive voltage source via the electrode clamp (attached to the metal needle tip), and the grounding electrode clamp was fixed to the aluminum foil-covered collector. A white SOD@SiO_2_ films could be obtained within 60 min. The electrospinning parameters were as follows: apply a voltage of 16 kV, collector rotation speed of 300 rpm, needle tip to collector distance of 10 cm, and solution flow rate of 4 mL/h. The electrospinning device is placed in an acrylic box and operated at 25-30 °C and 40-60 % RH. To prepare a transparent SOD@SiO_2_ film, the as-prepared white SOD@SiO_2_ film was thermally cross-linked at 160 °C in an oven. The fibers in the film rapidly underwent cross-linking reactions, yielding a transparent SOD@SiO_2_ film after 10 minutes.

**1.4 Characterization of X-ray Excited Luminescence**

The total X-ray absorption coefficient varying with photon energy was obtained from the XCOM database provided by the National Institute of Standards and Technology (NIST). Further, the attenuation efficiency (AE, %) was calculated using formula (1):

$AE=\left( 1-e^{-t\rho d} \right)\times100\%$ (1)

where *ρ* (g/cm^3^) is the density of test sample and *d* (cm) is the thickness of test sample.

The X-ray light yield (LY) and limit of detection (LoD) were measured using a Tungsten X-ray tube (MOXTEK) equipped on a fluorescence spectrometer (Edinburgh Instruments FLS1000). LY was determined using a reference method with the commercially available CsI(Tl) crystal as the standard scintillator. To minimize thickness influences, all samples were prepared with a thickness of ≥ 1 mm. Both the sample and the standard scintillators were irradiated using an X-ray tube operating at a tube voltage of 50 kV and current of 20 μA. The emitted photons (𝑃_measured_) were quantified and were normalized to 100% X-ray attenuation efficiency (*AE*) according to equation (2):

$P_{normalized}=\frac{P_{measured}}{AE(d)}$ (2)

where *AE*(*d*) is the attenuation efficiency at the actual thickness of the photon energy of 10 keV (the peak energy of the W tube). The attenuation efficiencies of CsI(Tl), SOD, SOD@SiO_2_ were calculated as 100%, 100% and 100%, respectively.

Light yields (LY) of samples can be calculated from equation (3):

${LY}_{sample}=\frac{P_{normalized}(sample)}{P_{normalized}(CsI(Tl))}\times{LY}_{CsI(Tl)}$ (3)

where LY_CsI (Tl)_ is 56000 photons/MeV.

LoD was analyzed at an X-Ray tube voltage of 6-10 kV and current of 7-15 μA. The emitted photons (𝑃_measured_) were quantified by FLS1000. LoD can be calculated using the 3*σ*/slope method, as expressed by equation (4):

$LoD= \frac{3\times SD}{slope}$ (4)

where SD represents the standard deviation derived from the signal intensities at the emission wavelength obtained from at least 11 consecutive background noise measurements. Slope refers to the gradient of the linear regression curve of the RL intensity of SOD and SOD@SiO_2_ as a function of varying X-ray dose rate.

In this work, the SOD@SiO_2_ film thickness for imaging was set to 0.1 mm, ensuring sufficient light output under conventional X-ray exposure while avoiding signal loss in thinner films and resolution degradation in thicker films.

For X-ray imaging, a SOD@SiO_2_ film was used as the imaging screen (dimensions 3×3 cm^2^, thickness 0.1 mm). X-ray imaging was performed using custom-built equipment with a tungsten X-ray tube (MOXTEK) operating at 50 kV and 200 μA as the excitation source, and a Canon M50 digital camera for photographing. The exposure time for photographs was 1 s, ISO3200. The spatial resolution of the image is quantified by the modulation transfer function (MTF), which is derived from Equation (5) through the acquisition and analysis of three plots containing *I_max_* and *I_min_* values using ImageJ software.

$MTF=\frac{I_{max}-I_{min}}{I_{max}+I_{min}}$ (5)

**1.5 Afterglow photo shooting parameters and 3D imaging software**

The device used to take the afterglow photos for this article was an iPhone 14 Pro Max. Shooting parameters: 24 mm *f* 1.78, ISO 5000. The grayscale data is processed using optimized NumPy vectorized operations, thereby achieving an efficient processing algorithm with a time complexity of O(n). The extraction of sample points employs a dynamic sampling algorithm:

| When the number of points exceeds the threshold “MAX_POINTS”： indices = np.linspace(0, total_points - 1, max_points, dtype=int) sampled_points = points[indices] |
| --- |

The algorithm maintains the characteristics of the data distribution while confining the processing time within a linear range. Tests reveal that processing a region of 1 million pixels requires merely 0.48 seconds (±0.05 seconds). **
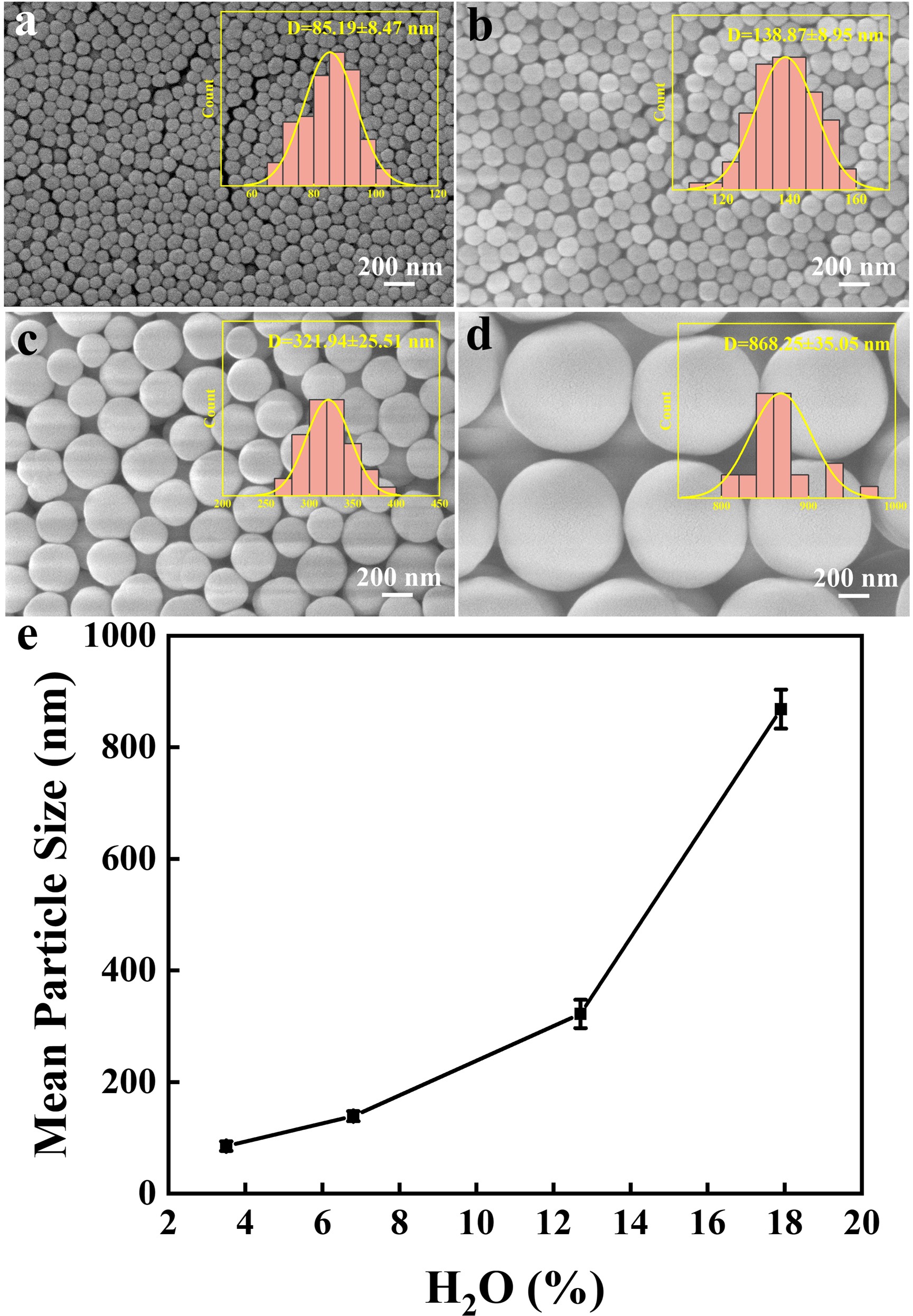
**

**Figure S1.** Control of SiO_2_ particle size by tuning water content during synthesis.
 a) 3.5%; b) 6.8%; c) 12.7%; d) 17.9%; e) Line graph of SiO_2_ particle size versus H_2_O concentration.**
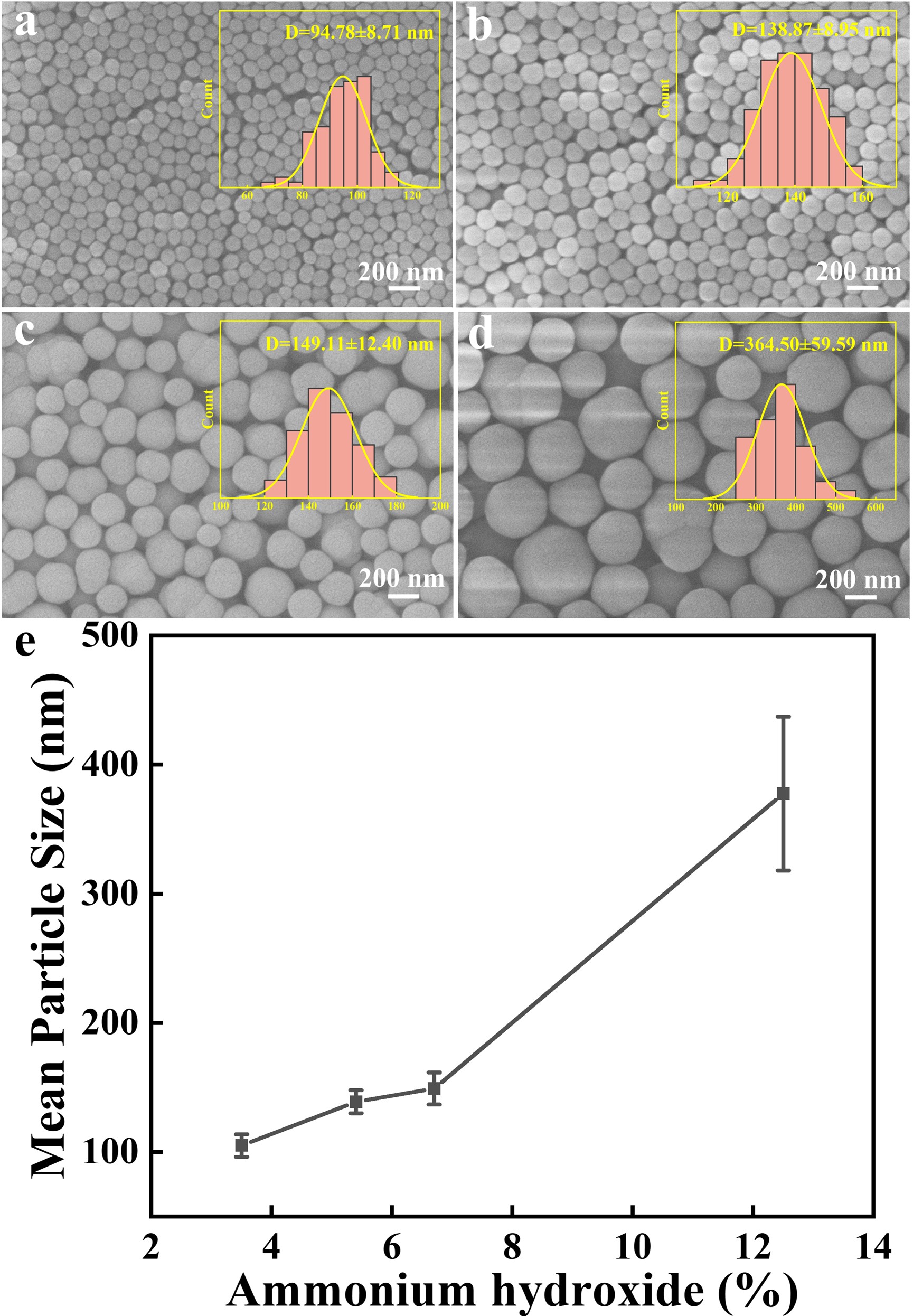
**

**Figure S2.** Control of SiO_2_ particle size by tuning the content of ammounium hydroxide during synthesis. a) 3.5%; b) 5.4%; c) 6.7%; d) 12.5%; e) Line graph of SiO_2_ particle size versus ammonium hydroxide concentration.


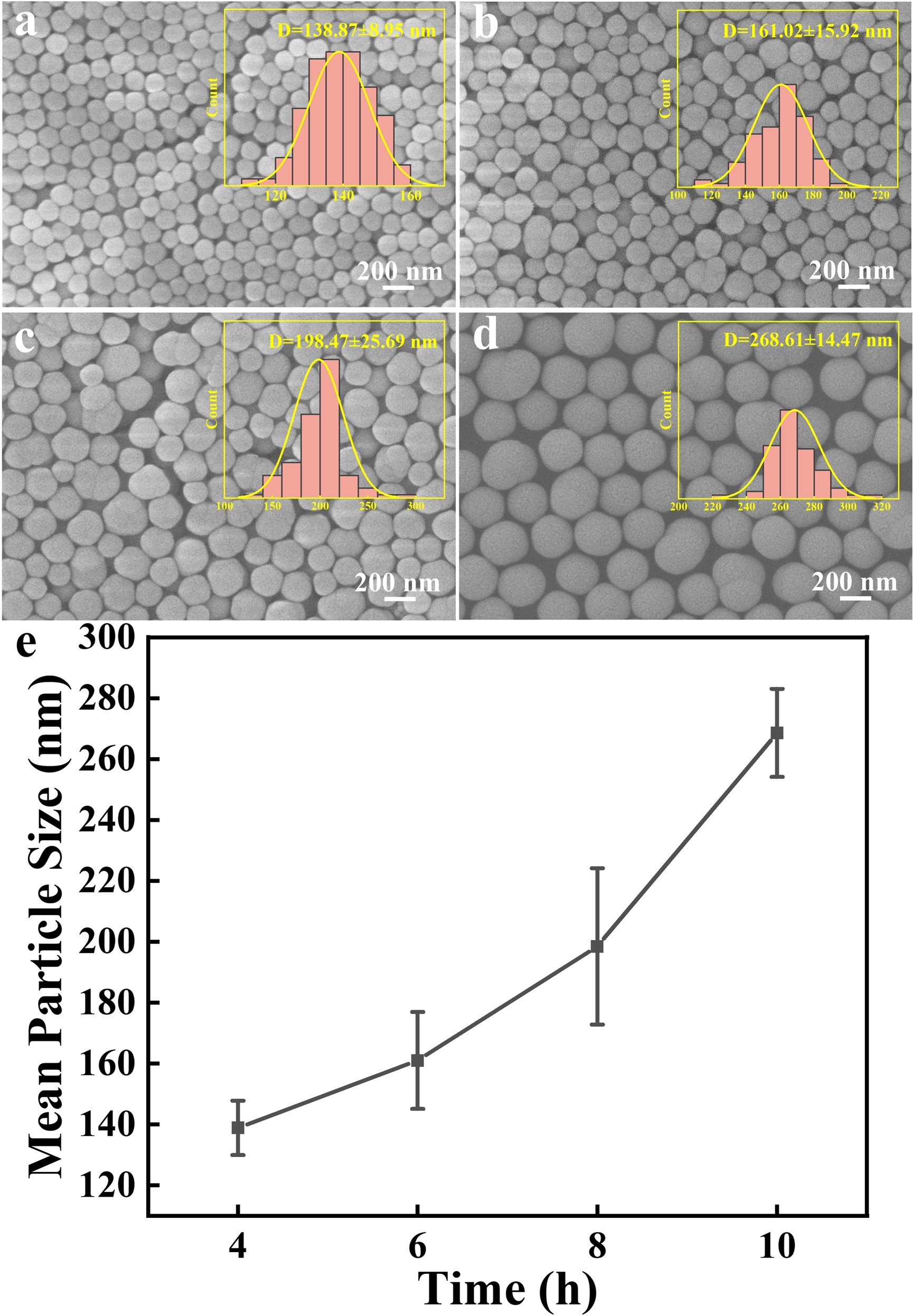


**Figure S3.** Control of SiO_2_ particle size by tuning the reaction time. a) 4 h; b) 6 h; c) 8 h; d) 10 h; e) Line graph of SiO_2_ particle size versus treatment time


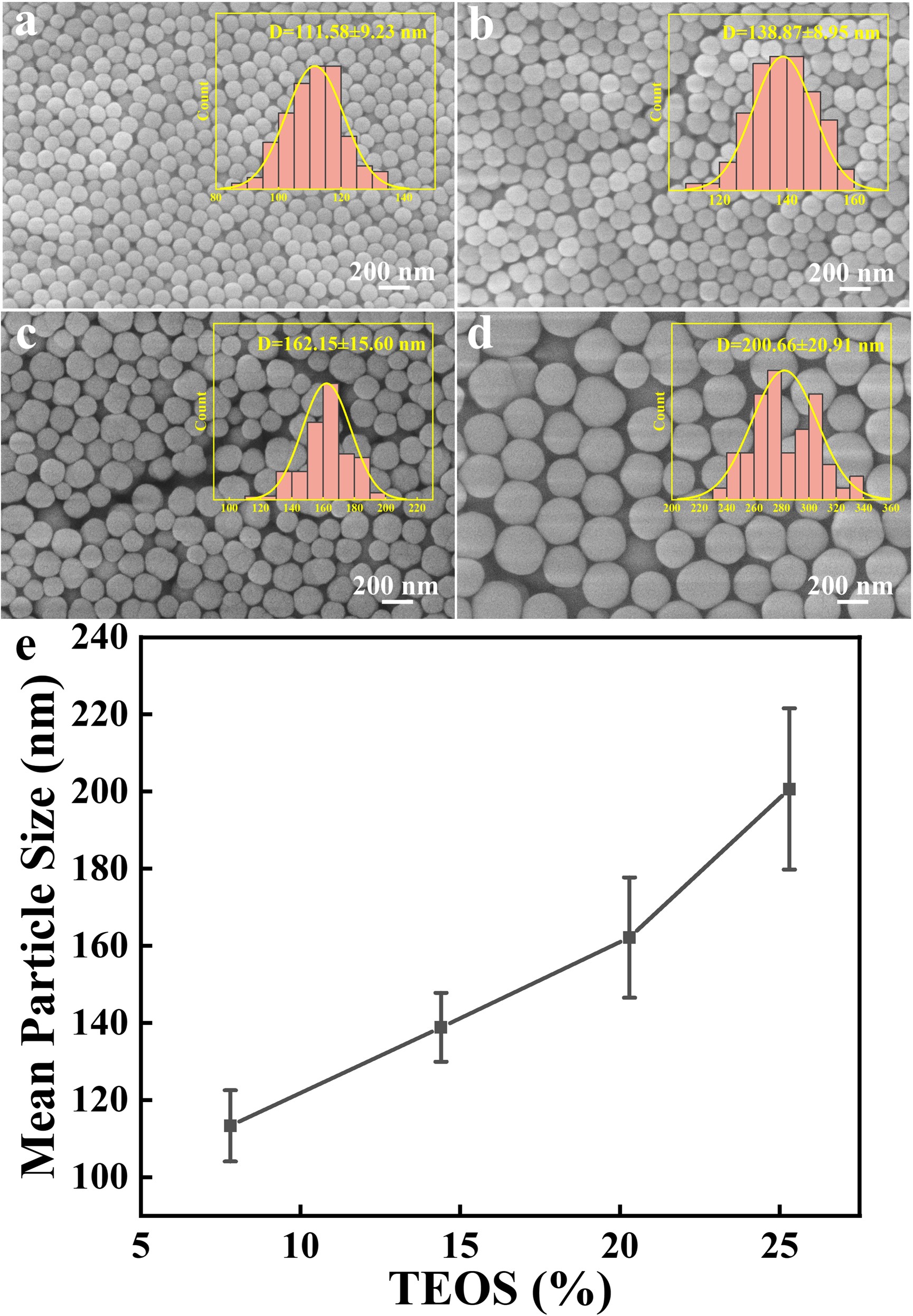


**Figure S4.** Control of SiO_2_ particle size by tuning TEOS content during synthesis. a) 7.8%; b) 14.4%; c) 20.3%; d) 25.3%; e) Line graph of SiO_2_ particle size versus TEOS concentration.


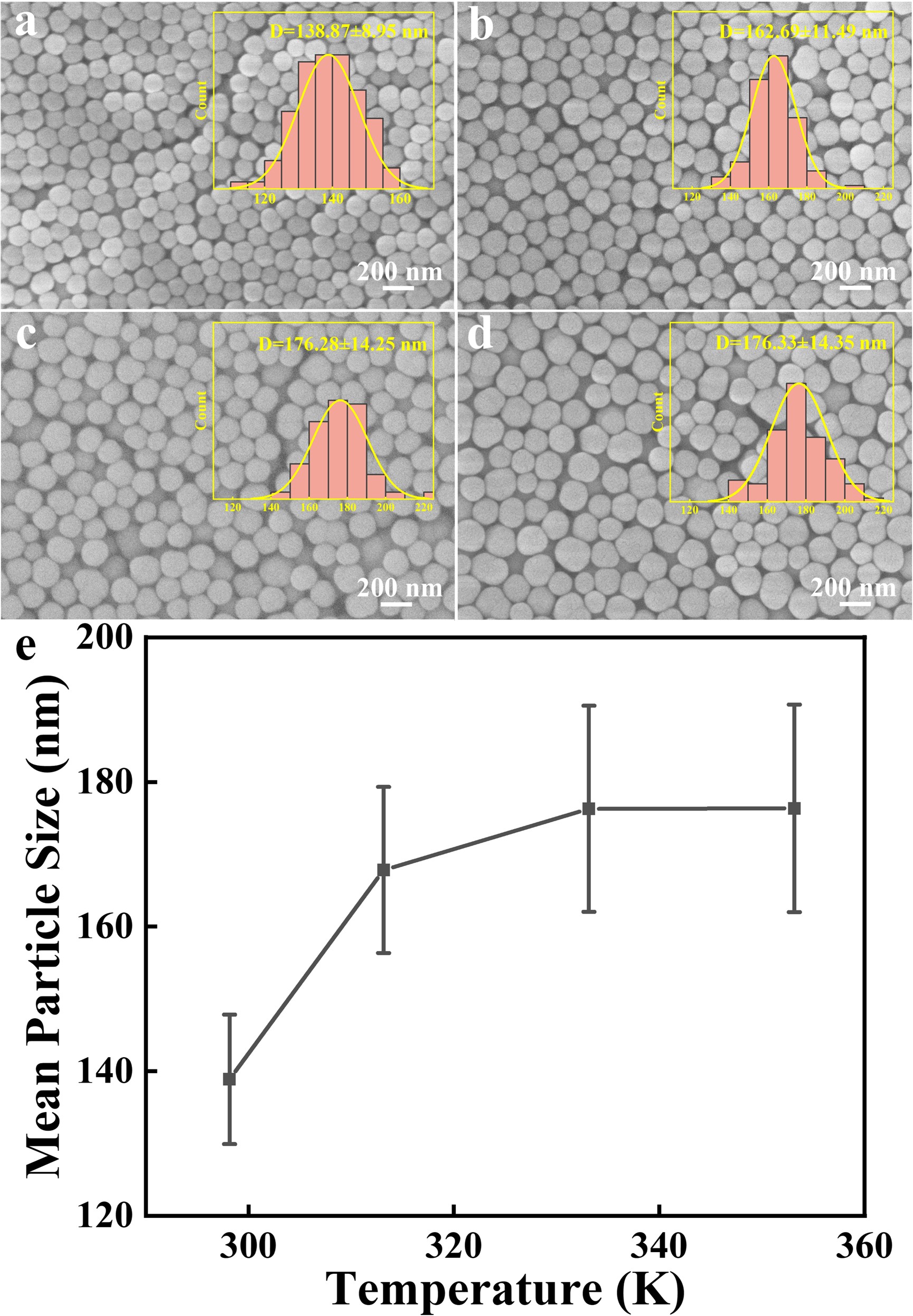


**Figure S5.** Control of SiO_2_ particle size by tuning the reaction temperature. a) 298 K; b) 313 K; c) 333 K; d) 353 K; e) Line graph of SiO_2_ particle size versus reaction temperature.


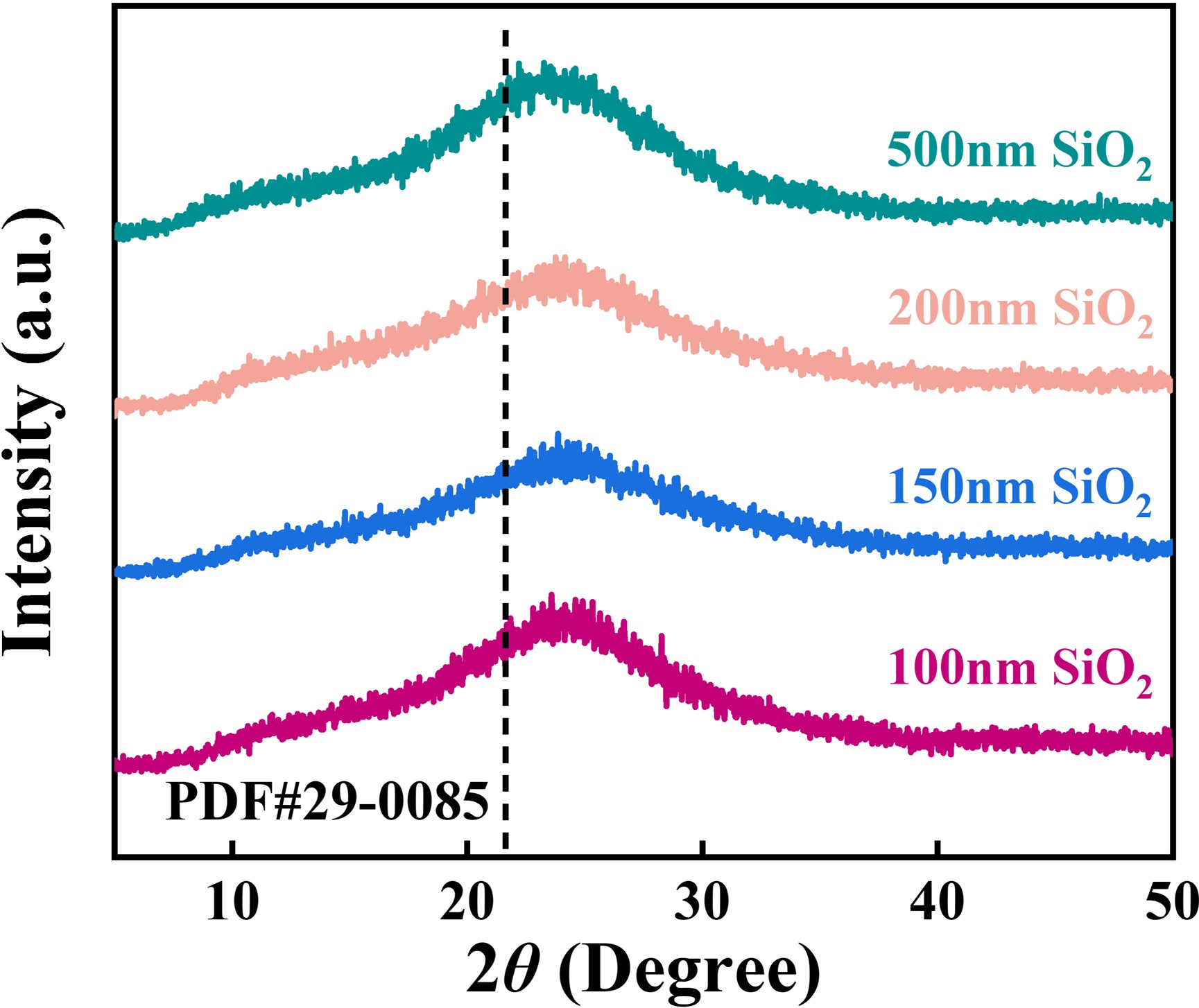


# **Figure S6.** XRD patterns of SiO_2_ nanospheres with different particle sizes.


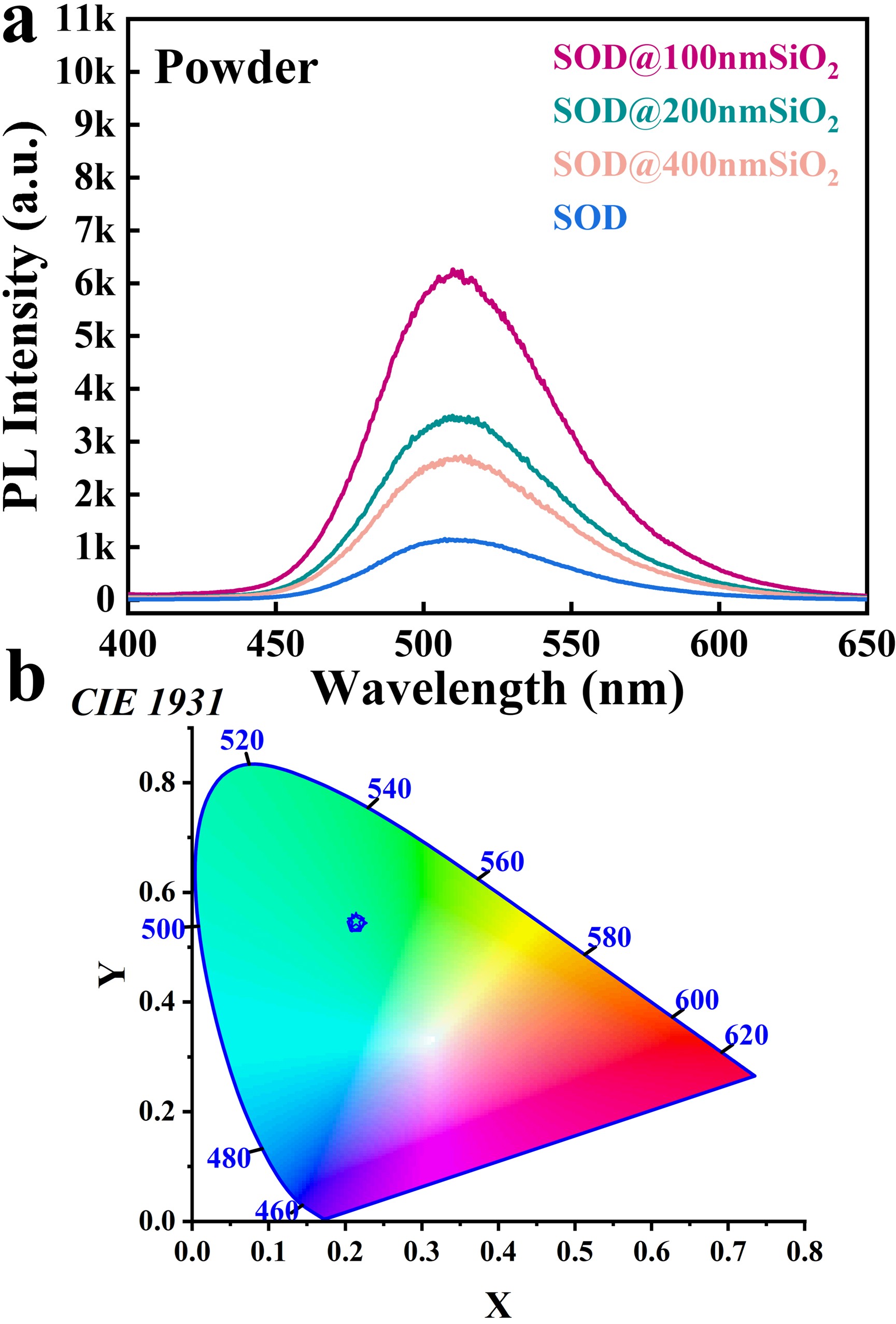


**Figure S7.** a) PL spectra and b) CIE coordinates of SOD@SiO_2_ powder prepared with different SiO_2_ particle sizes.

# **Table S1.** The table of PLQY parameters of SOD film and SOD@SiO_2_ film.

| **Samples** | **PLQY (%)** |
| --- | --- |
| SOD | 30.27 |
| SOD@SiO_2_ | 35.57 |
| SOD film | 41.73 |
| SOD@SiO_2_ film | 54.50 |


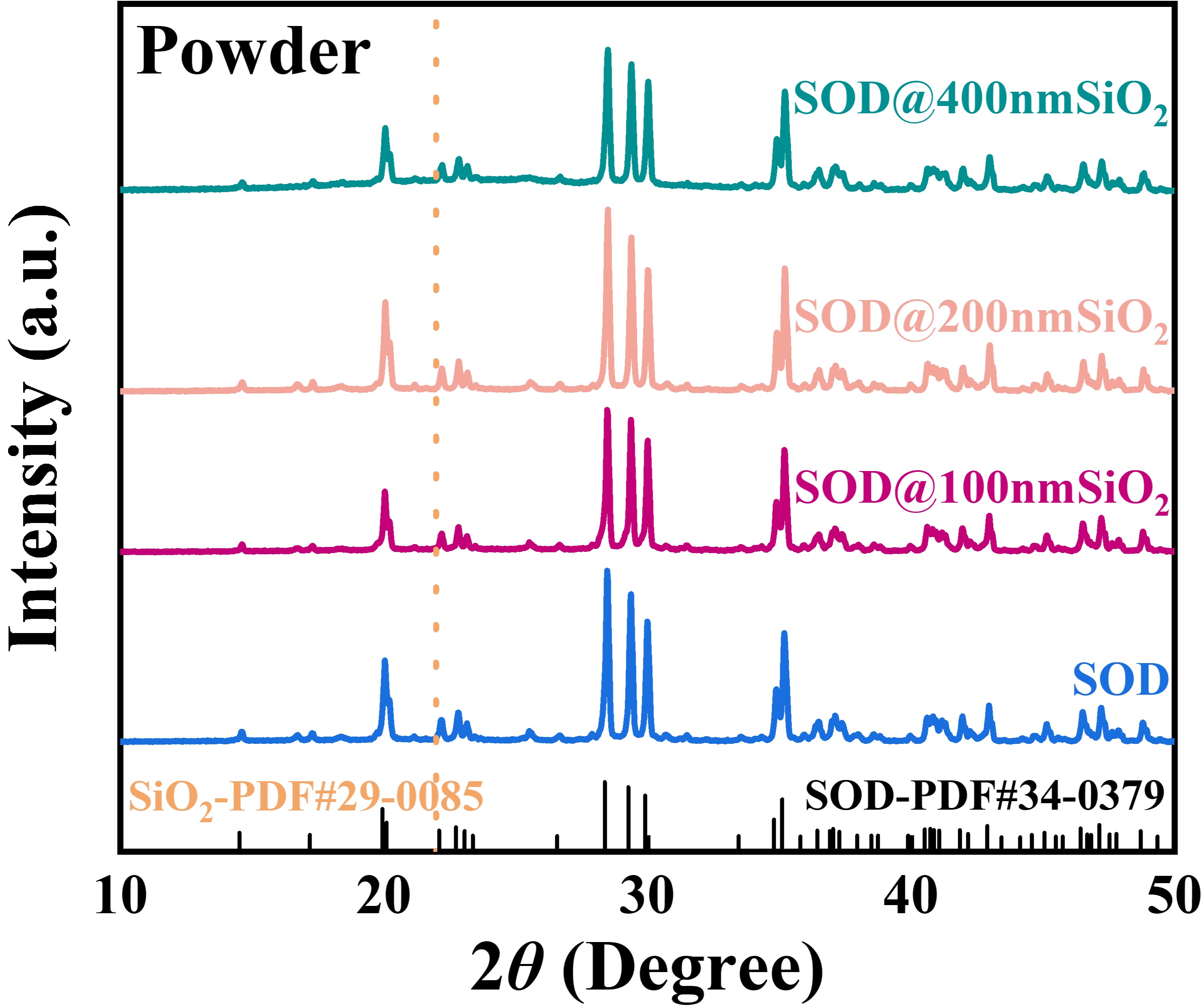


# **Figure S8.** XRD patterns of SOD and SOD@SiO_2_ powder coating different sizes SiO_2_ particle.


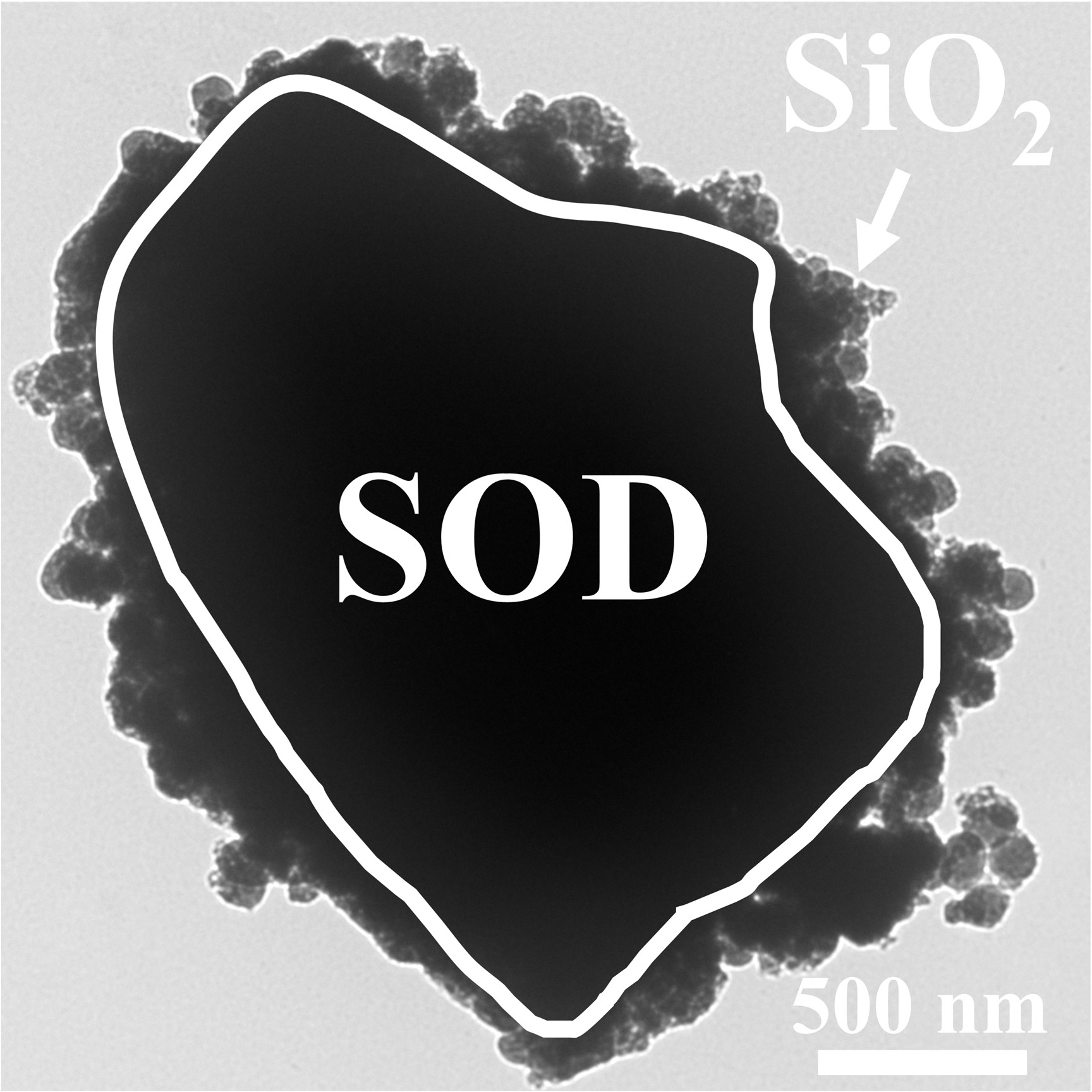


# **Figure S9.** The TEM image of SOD@SiO_2_ powder.


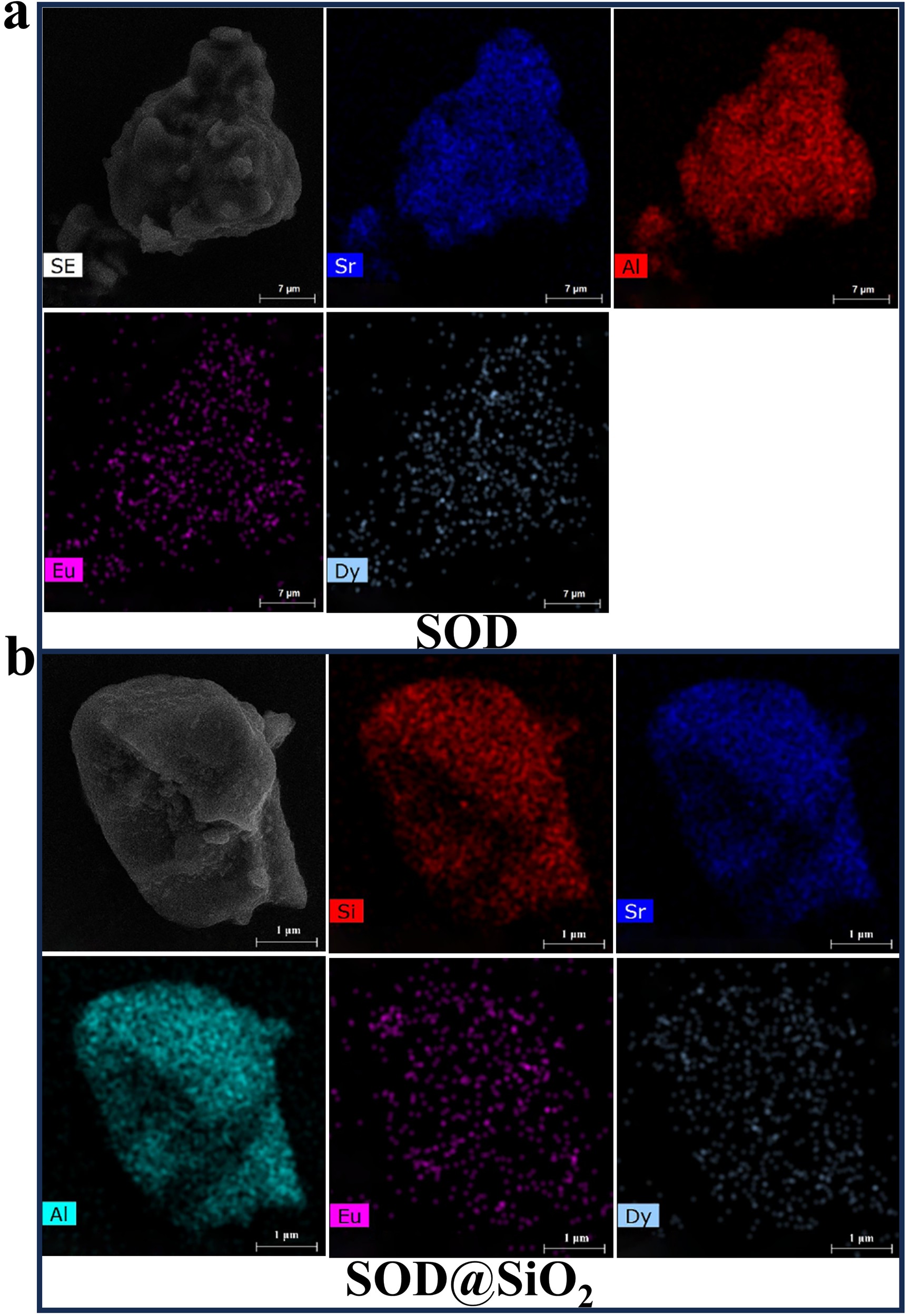


# **Figure S10.** The EDS mapping of a) SOD and b) SOD@SiO_2_ powder.


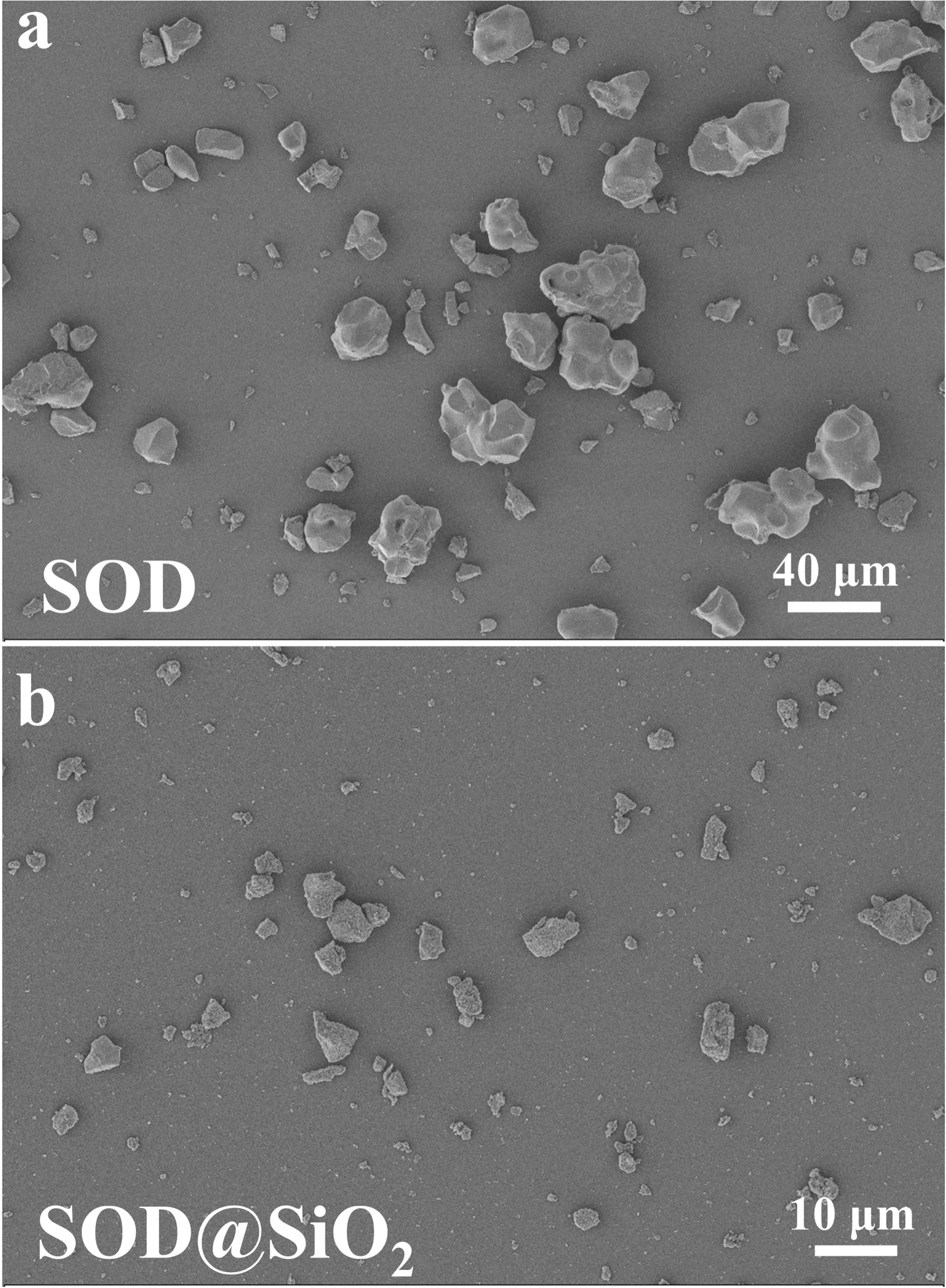


# **Figure S11.** The SEM images of a) SOD and b) SOD@SiO_2_ powder.


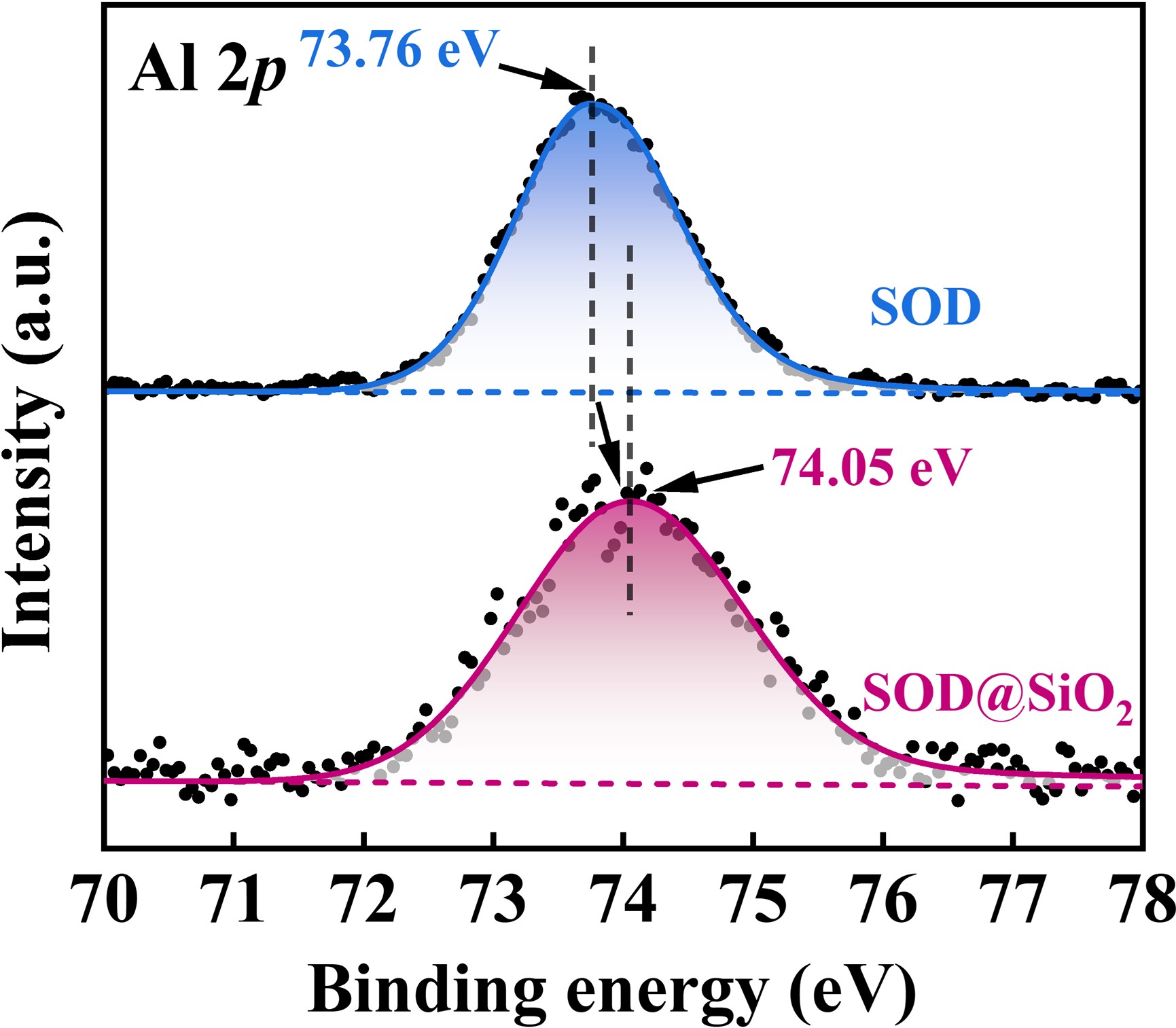


# **Figure S12.** The Al 2*p* XPS spectra of SOD with or without SiO_2_ coating.


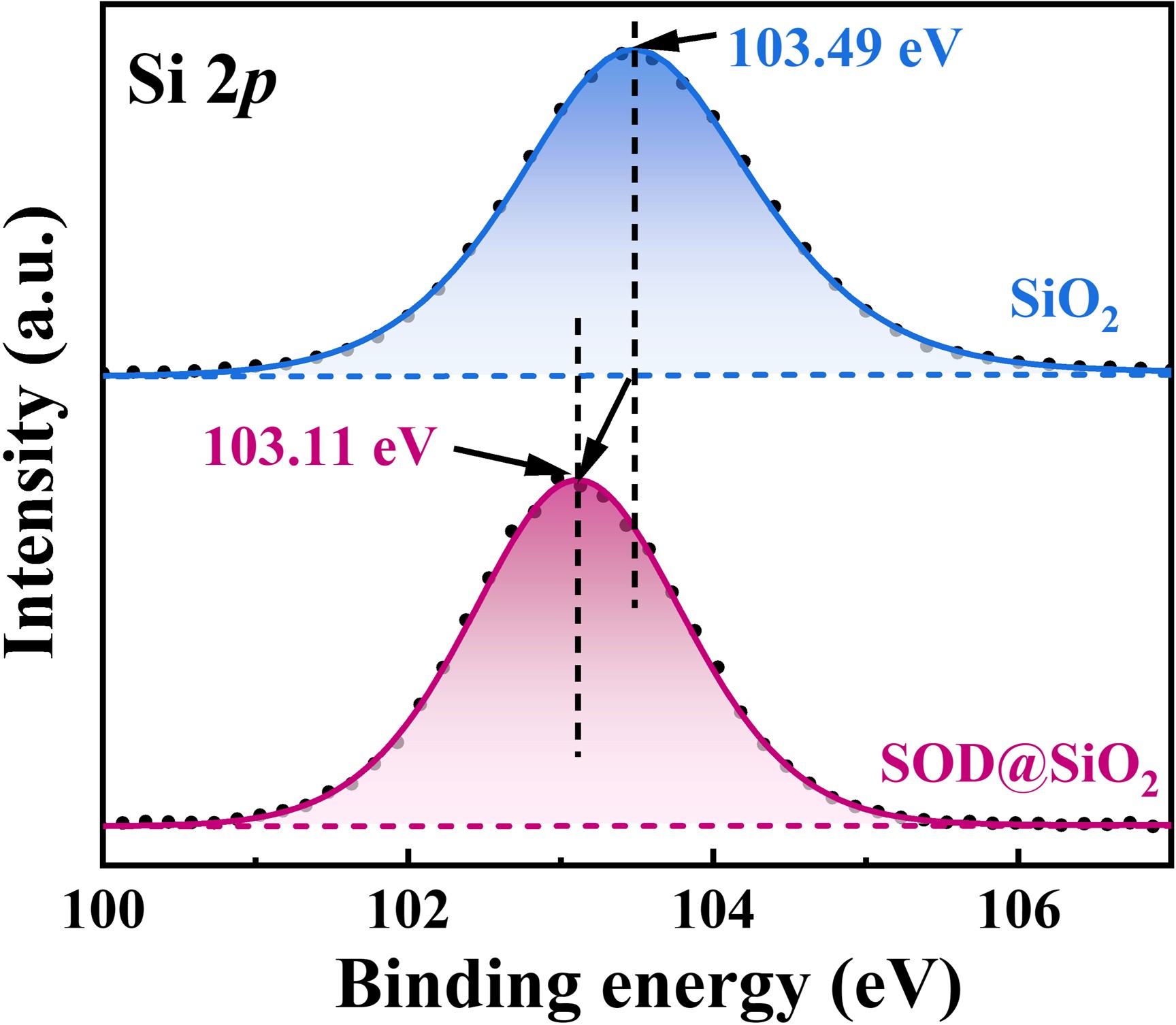


# **Figure S13.** The Si 2*p* XPS spectra of SOD with or without SiO_2_ coating.


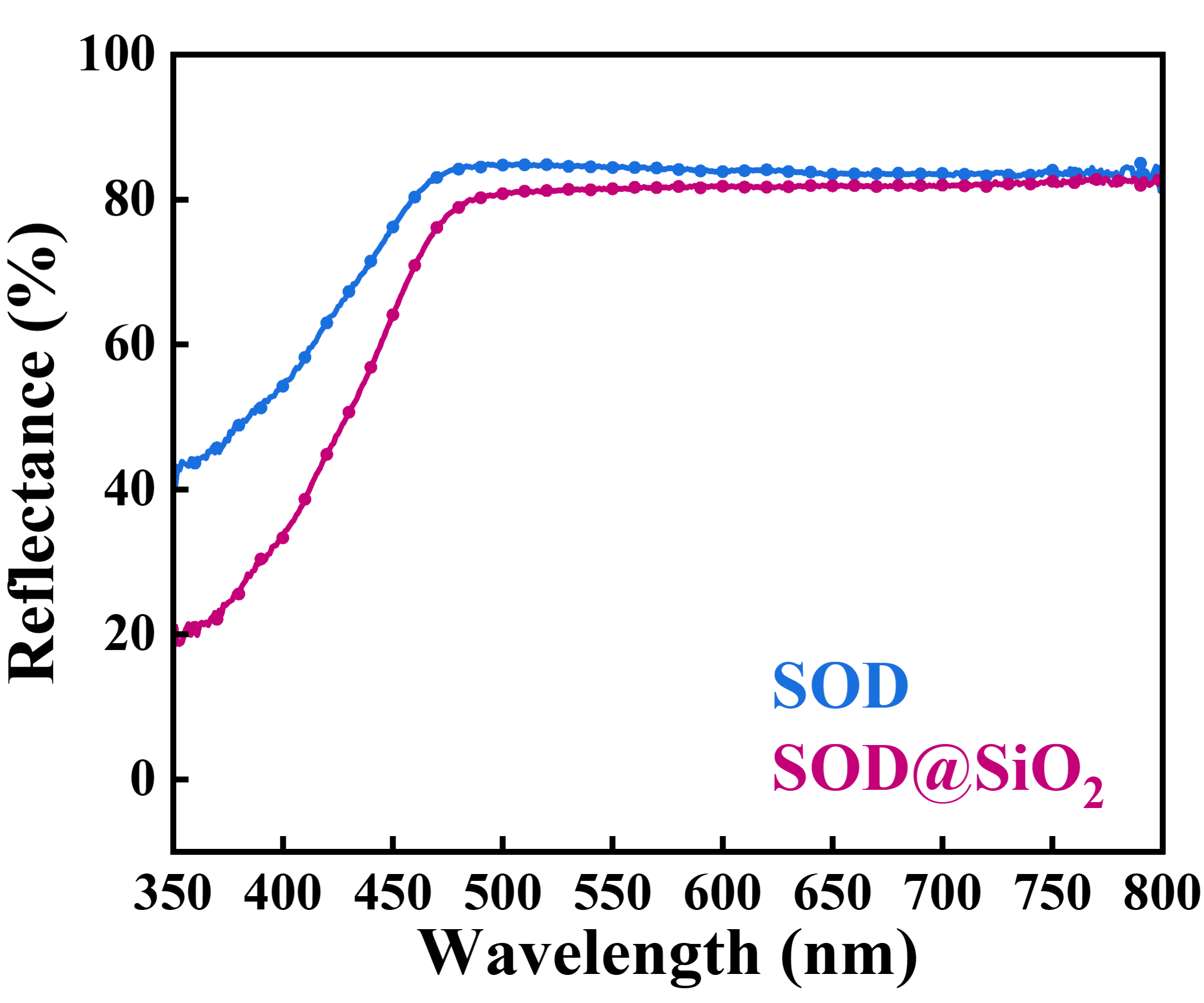


# **Figure S14.** Diffuse reflectance spectra of SOD and SOD@SiO_2_ powder.

**
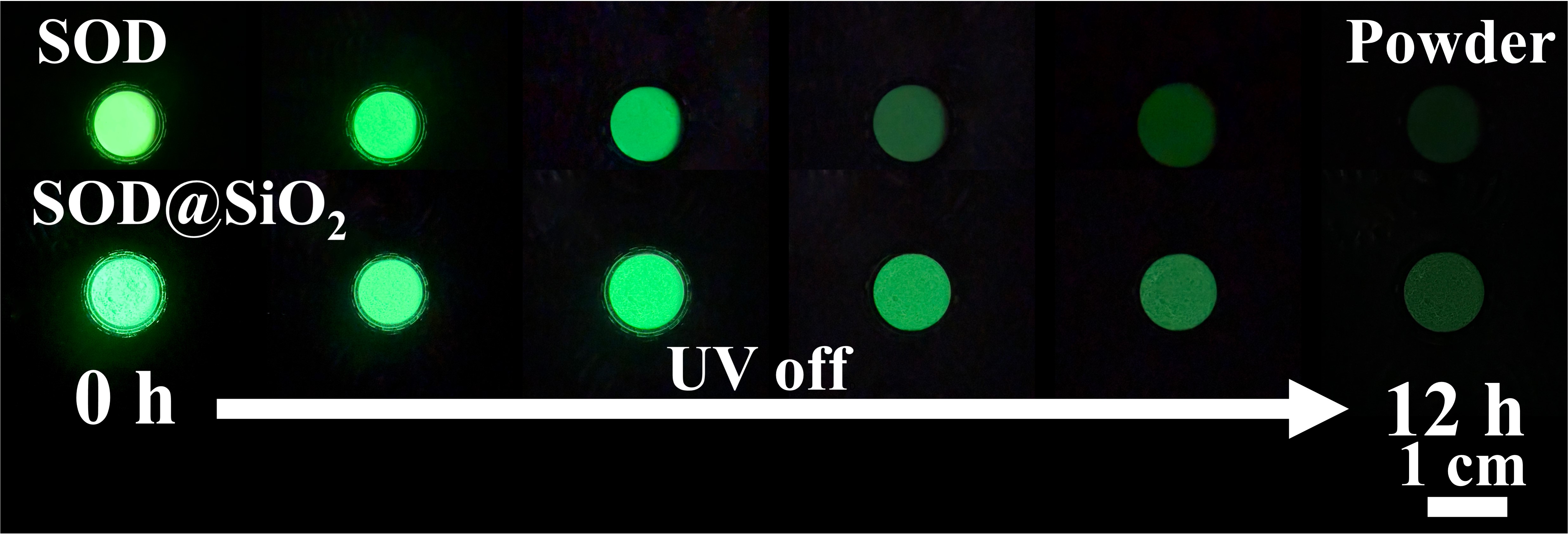
**

# **Figure S15.** The afterglow images of SOD and SOD@SiO_2_.


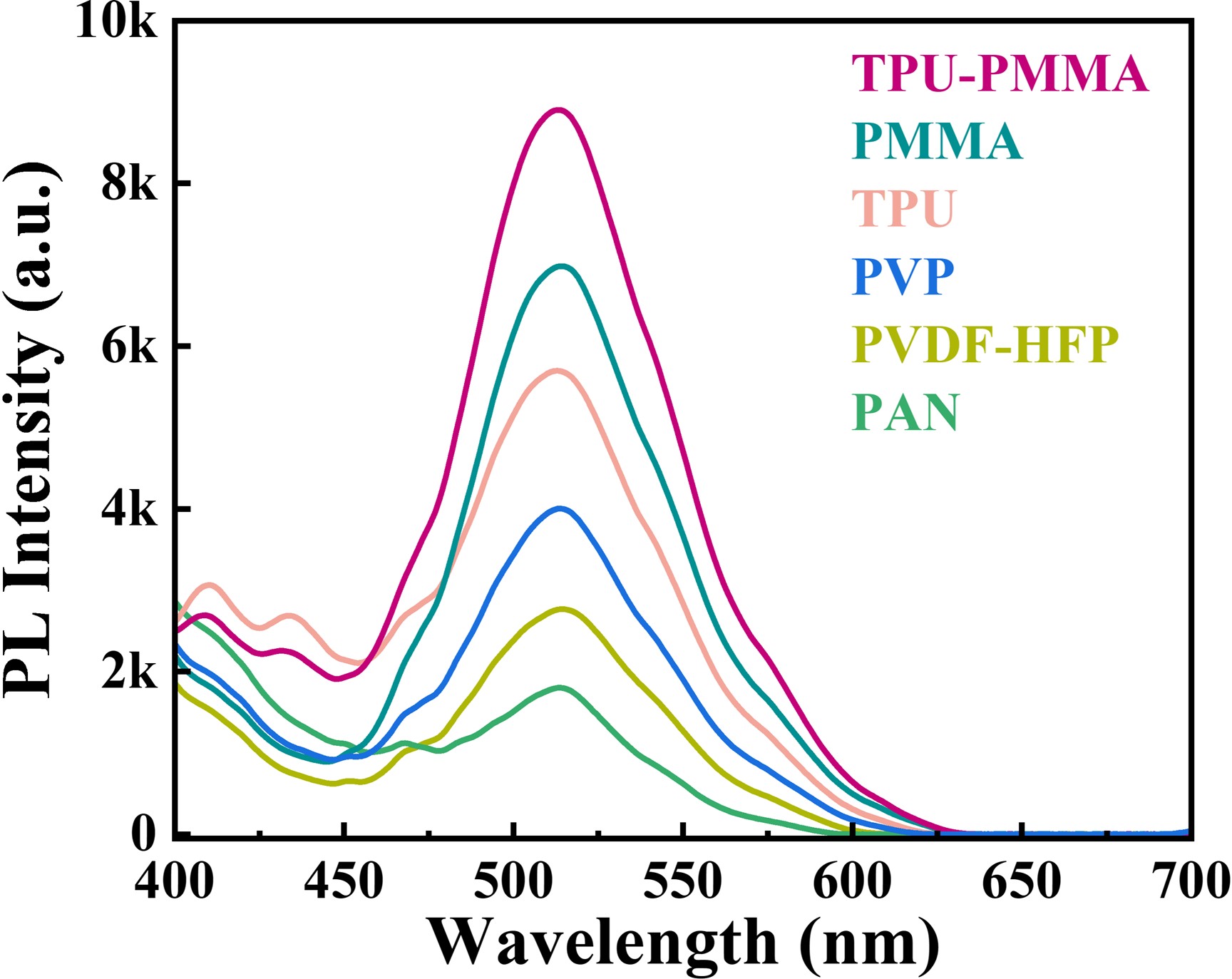


**Figure S16.** PL spectra of SOD@SiO_2_ in various matrices. The abbreviations are as follows: polymethylmethacrylate (PMMA), thermoplastic polyurethanes (TPU), polyvinylpyrrolidone (PVP), polyvinylidene fluoride-hexafluoropropylene (PVDF-HFP) and polyacrylonitrile (PAN). DMF and Acetone were used as the universal solvents.


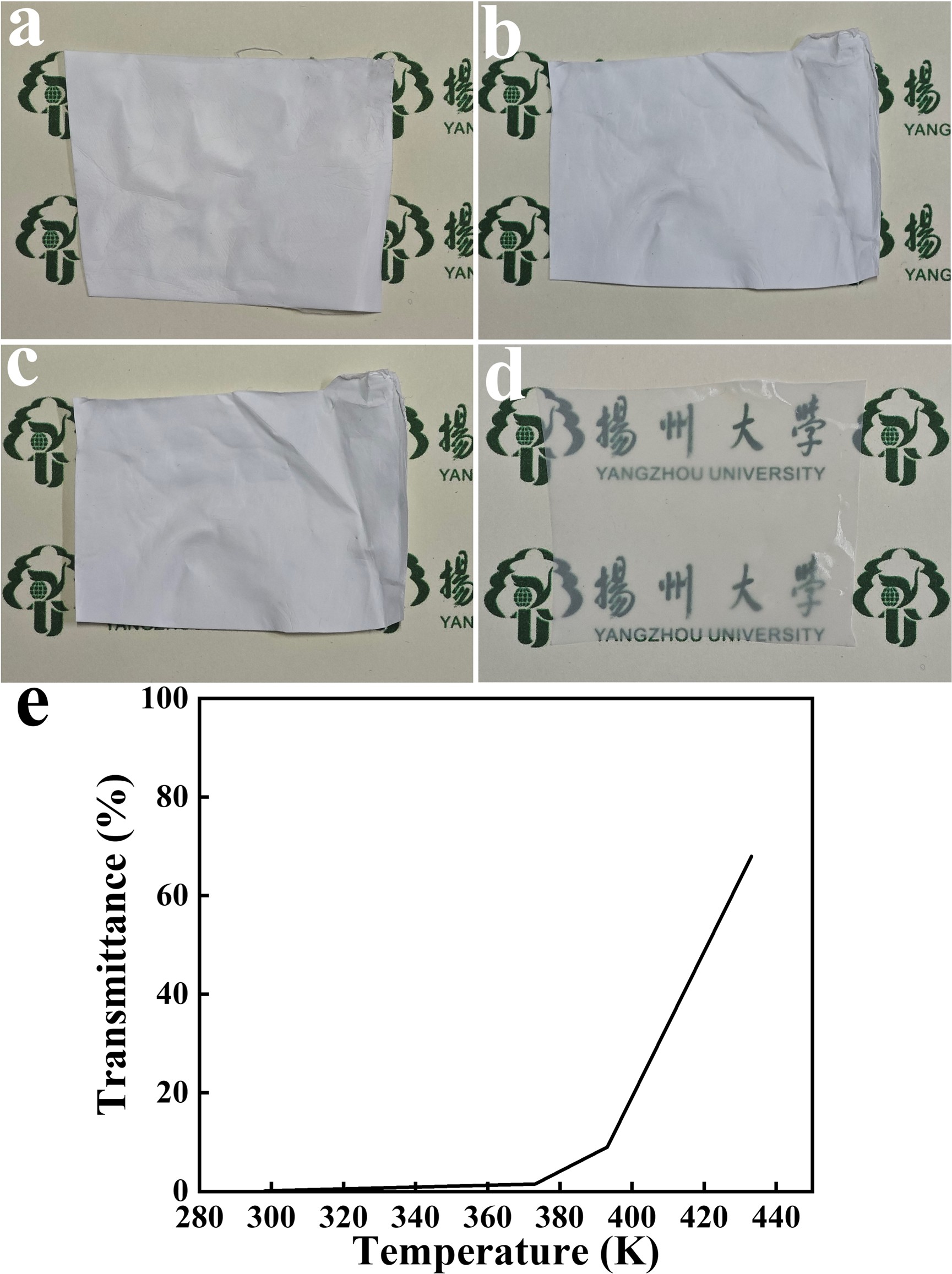


**Figure S17.** The temperature-dependent transmittance of films without crosslinking agent TTMAP. (a) 298 K, (b) 373 K, (c) 393 K, (d) 433 K. (e) Transmittance variation as a function of treatment temperature.


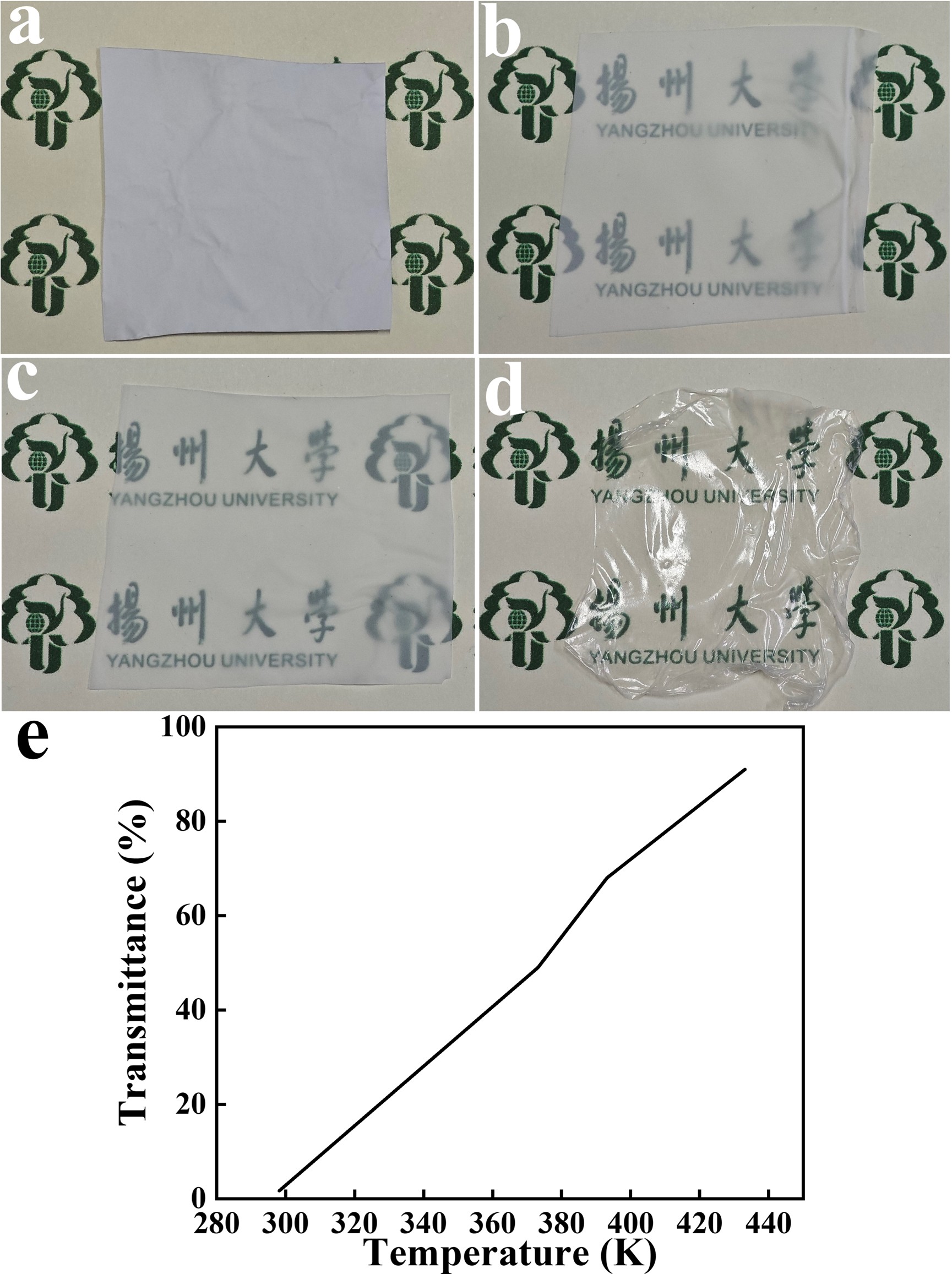


**Figure S18.** Temperature-dependent optical transmittance of TTMAP-crosslinked polymer films. (a) 298 K, (b) 373 K, (c) 393 K, (d) 433 K. (e) Transmittance vs. temperature profile demonstrating enhanced thermal stability up to 433 K.


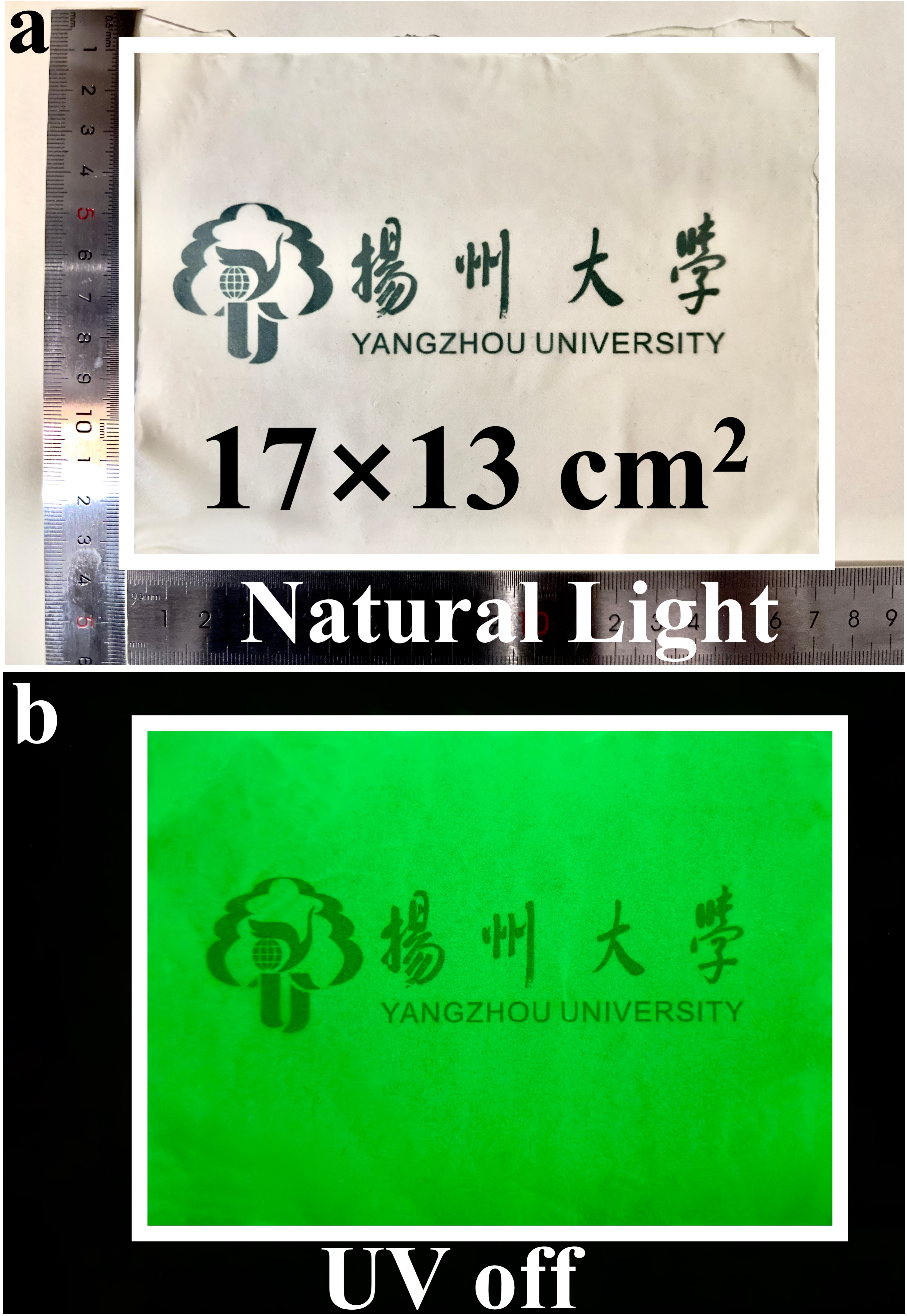


**Figure S19.** A large-area transparent SOD@SiO_2_ film. a) Under natural light; b) Afterglow image after UV light off.


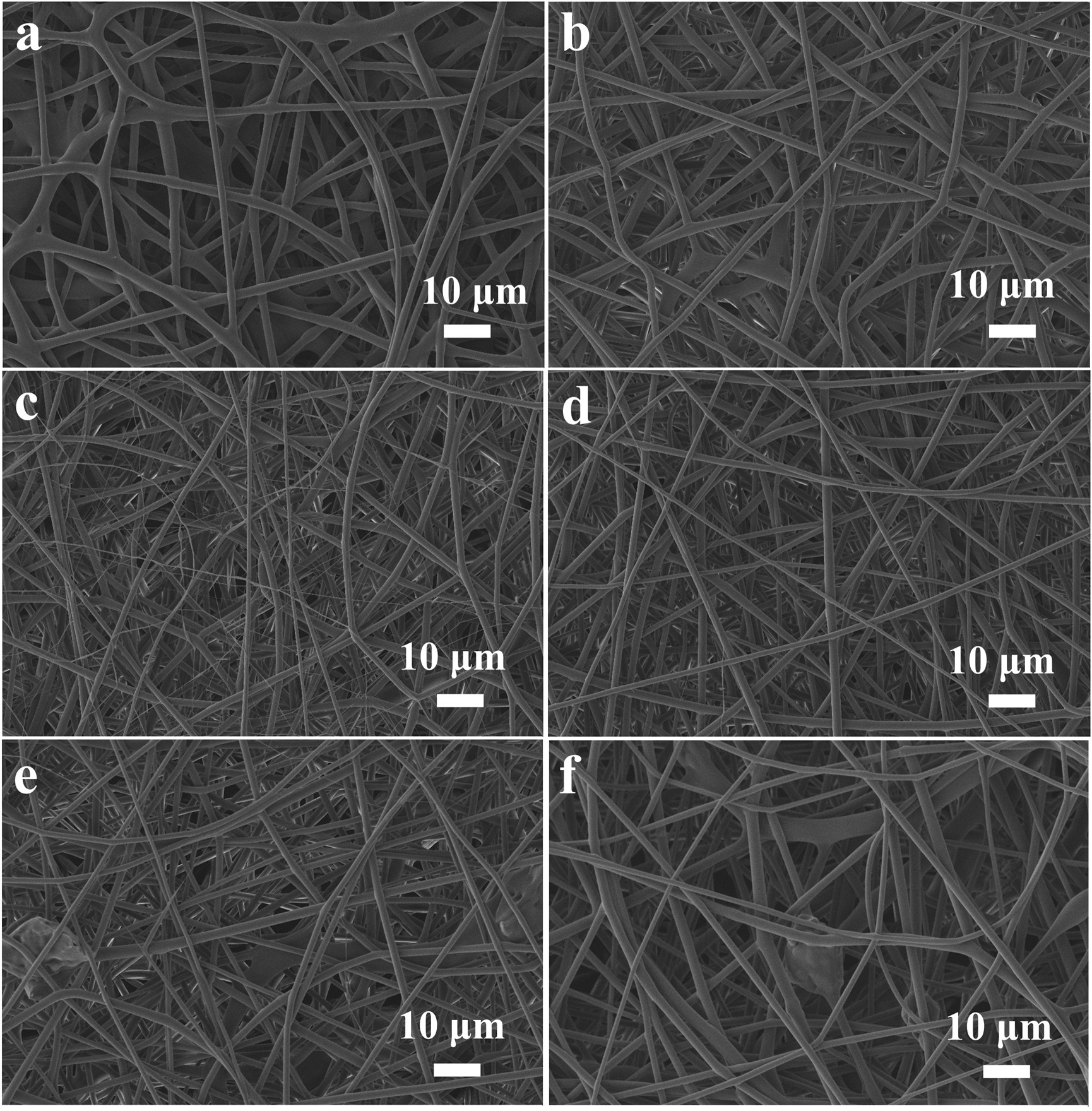


**Figure S20.** SEM images of films with different SOD@SiO_2_ mass contents. a) 3%; b) 5%; c) 10%; d) 15%; e) 20%; f) 25%.


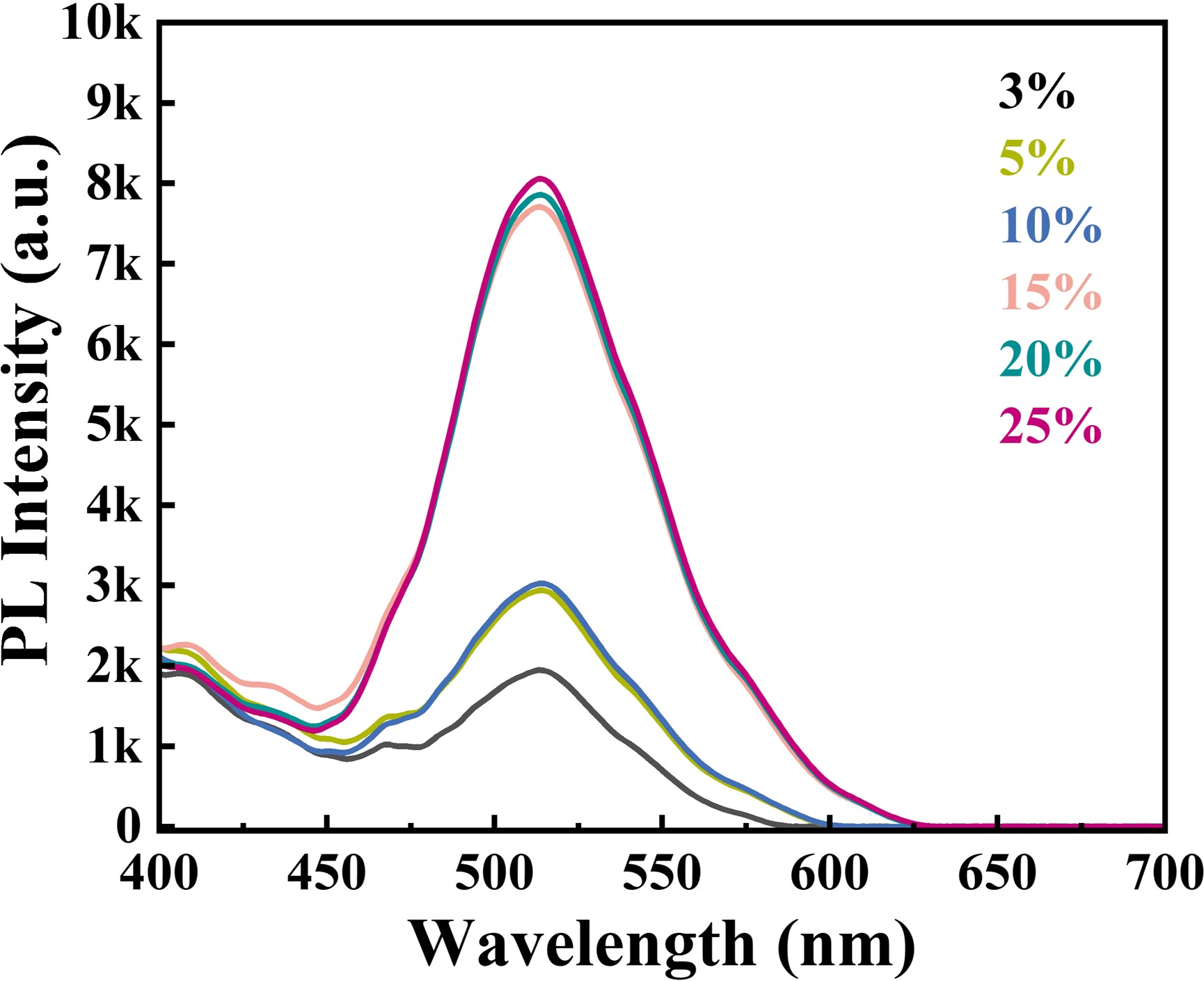


# **Figure S21.** PL spectra of films with different SOD@SiO_2_ additive amounts.

*λ_ex_ = 365 nm*


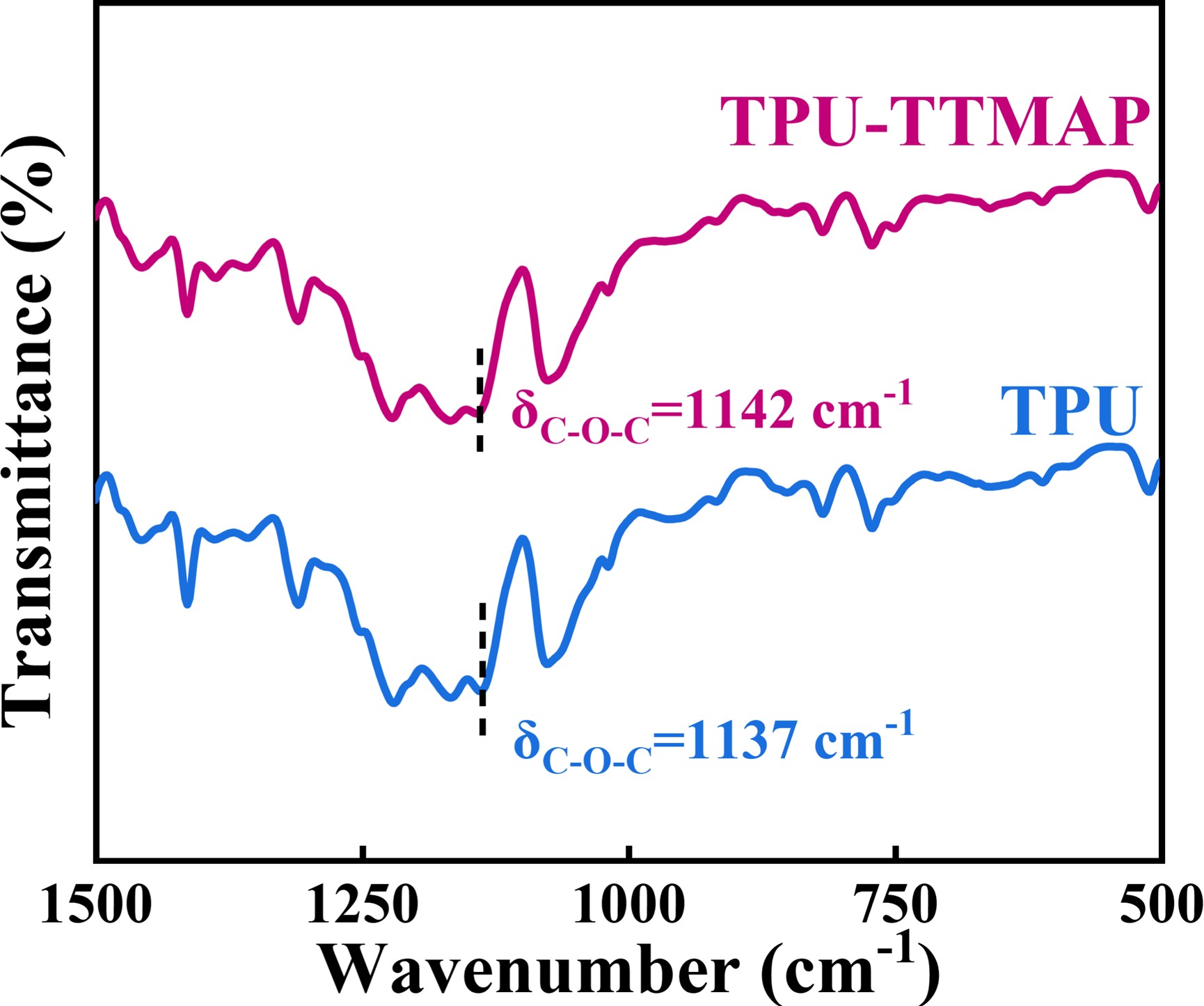


# **Figure S22.** The FTIR spectra of TPU-TTMAP films and TPU films.


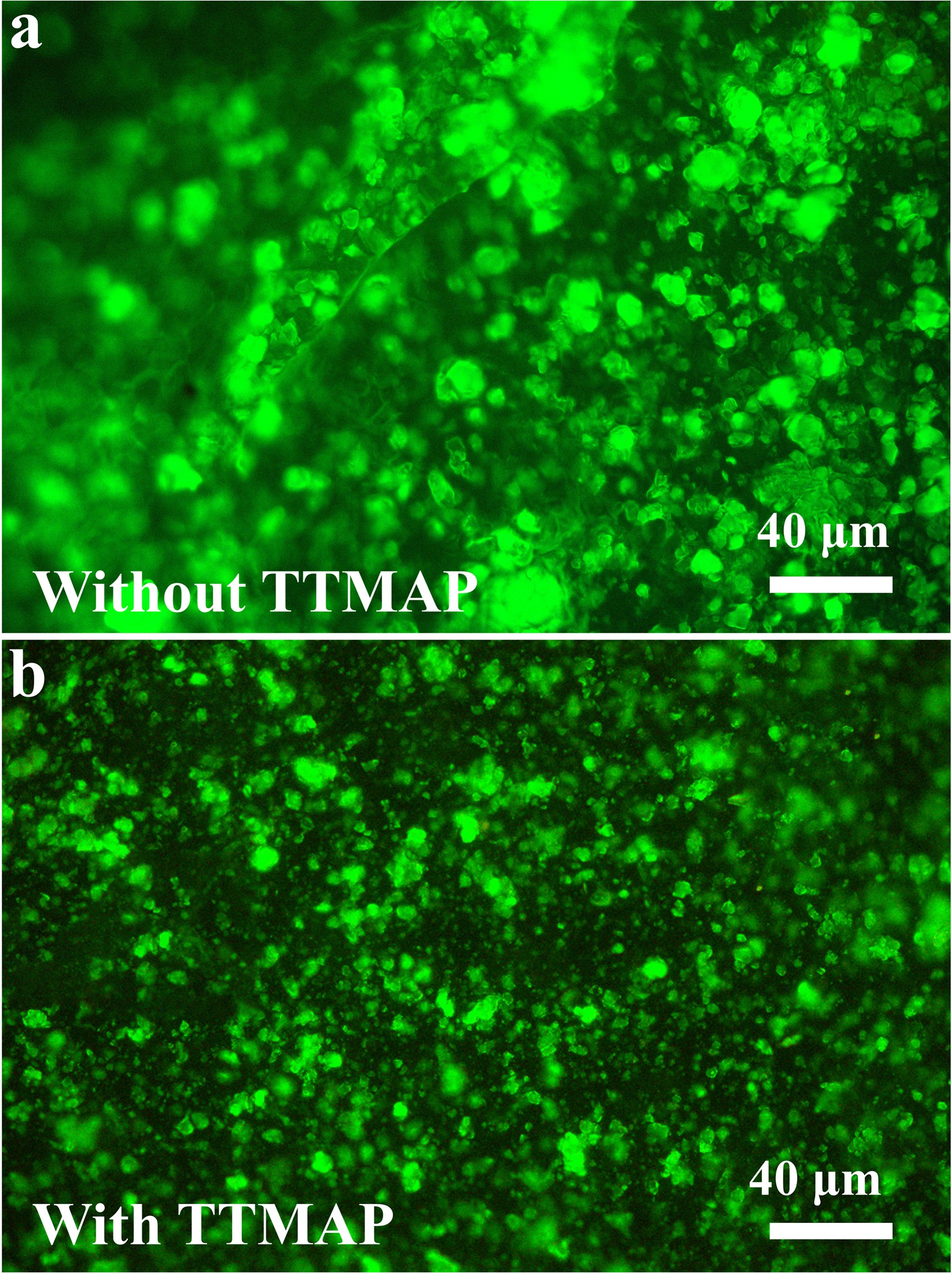


**Figure S23.** Fluorescence images of SOD@SiO_2_ films with and without TTMAP. a) Without TTMAP; b) With TTMAP.


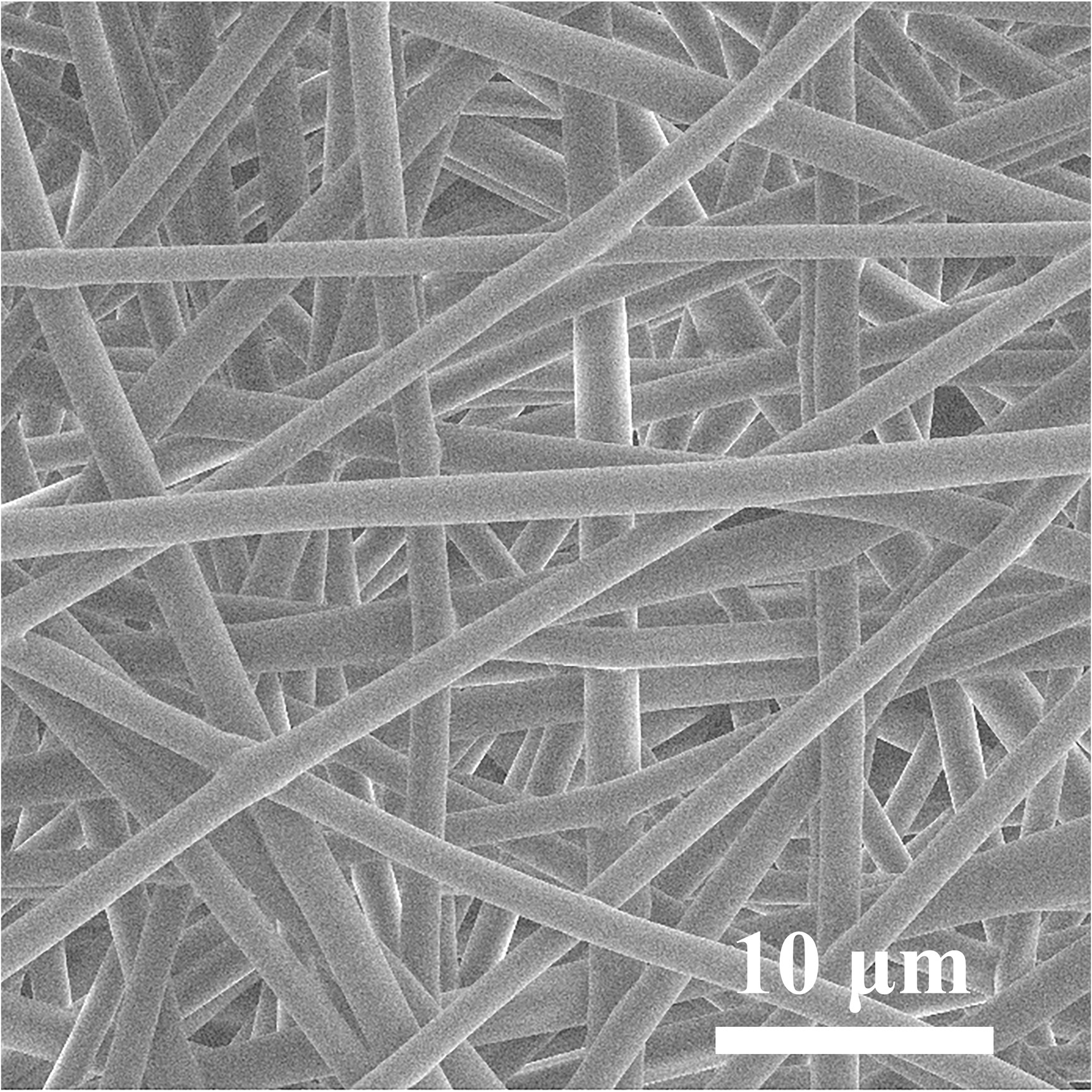


# **Figure S24.** SEM image of SOD@SiO_2_ film before heat treatment.


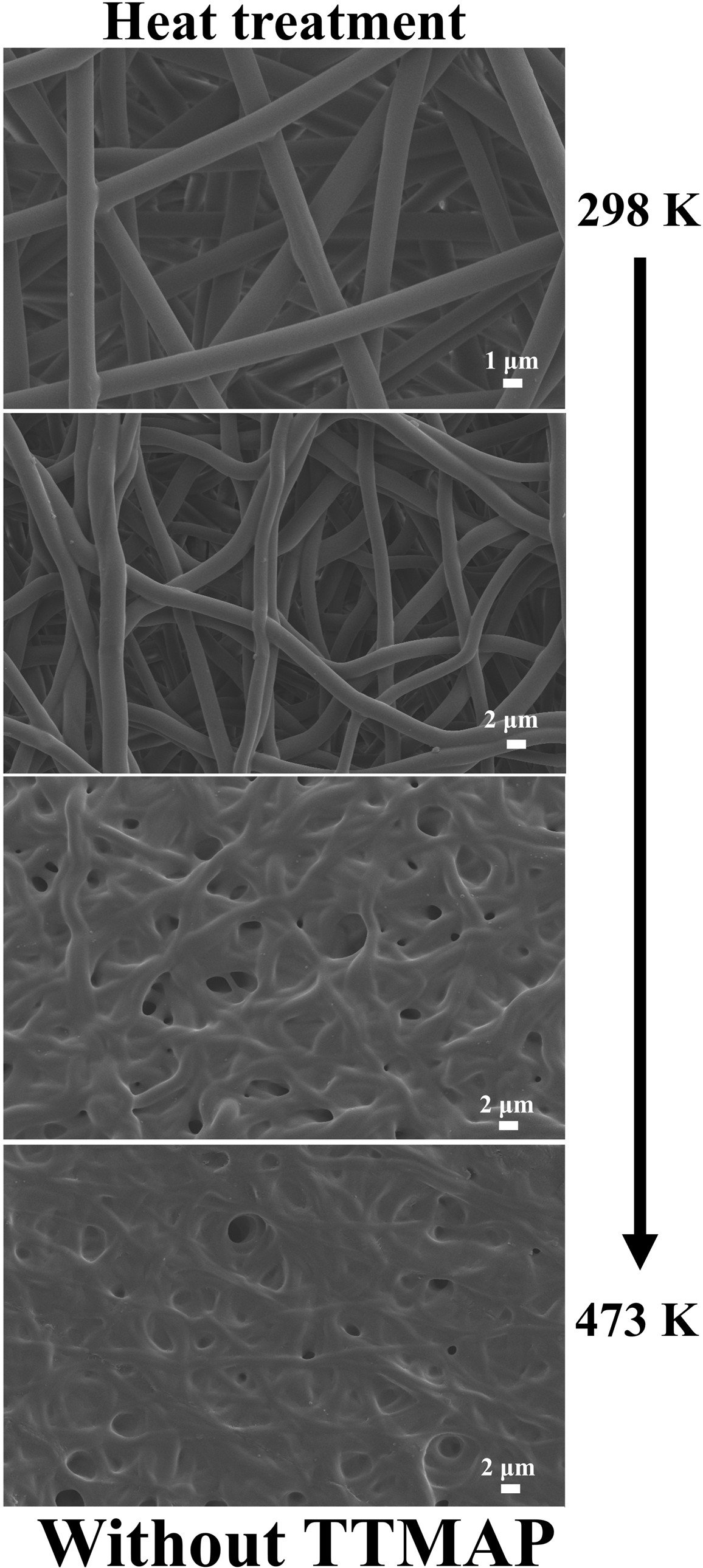


# **Figure S25.** SEM images of non-crosslinked films under thermal treatment (without TTMAP).


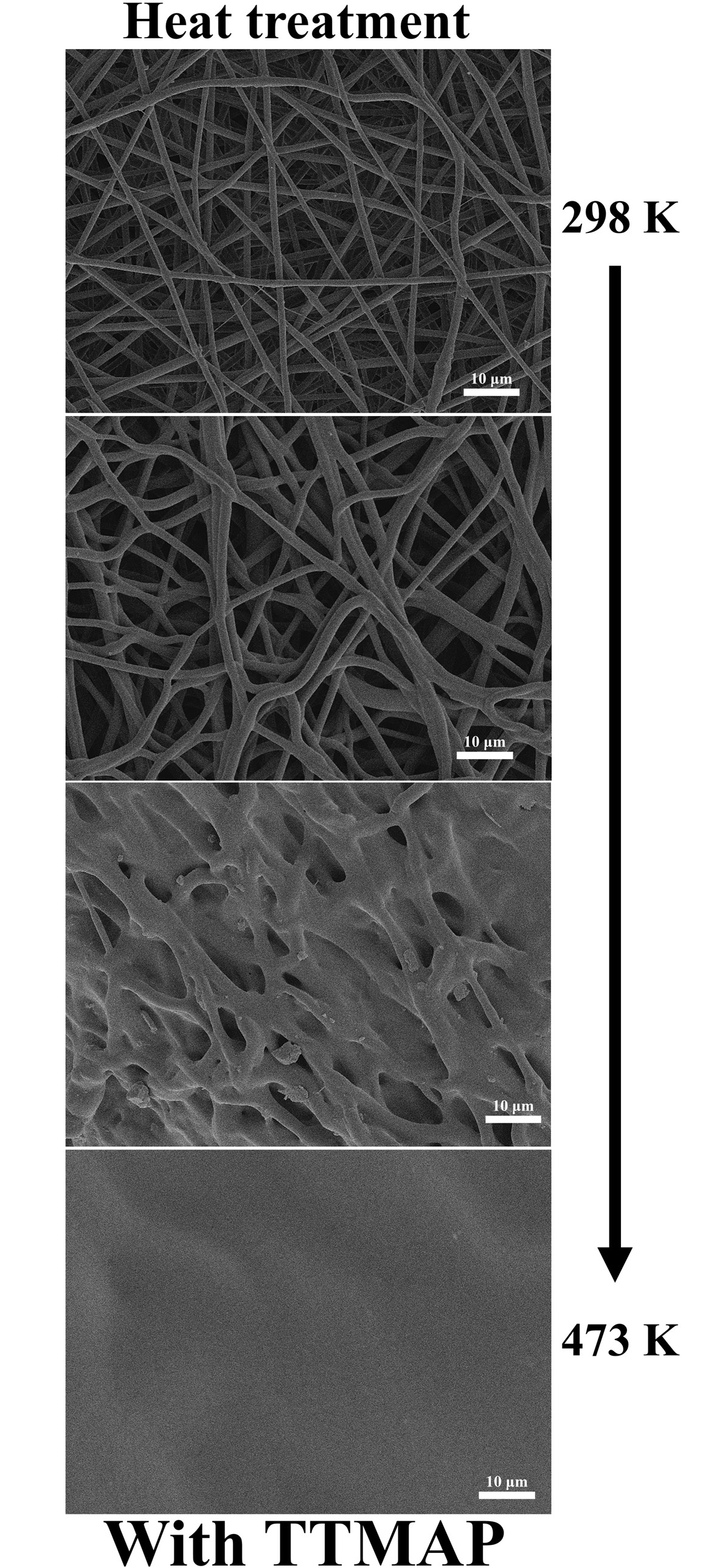


# **Figure S26.** SEM images of crosslinked films under thermal treatment (with TTMAP).


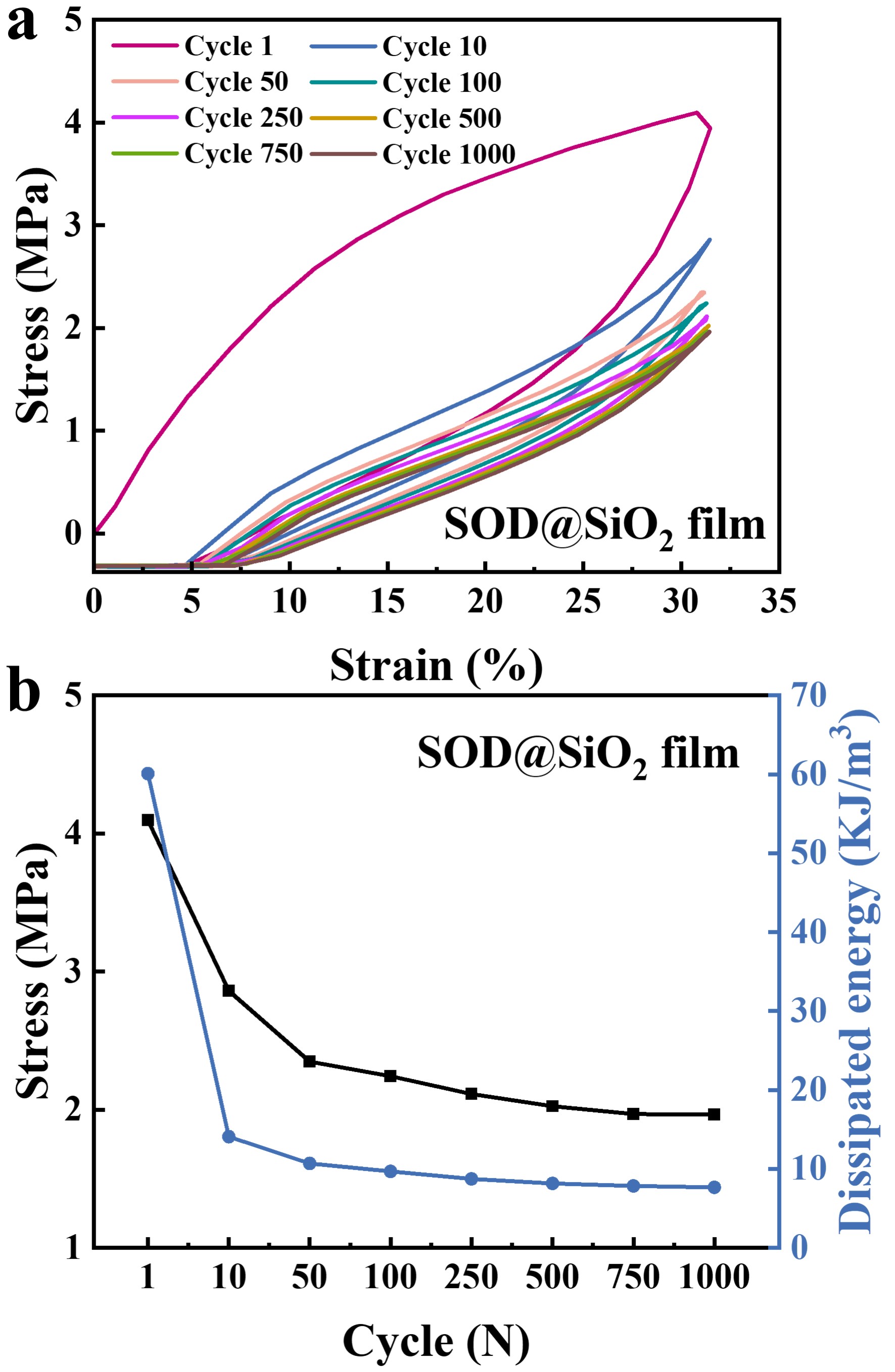


# **Figure S27**. Mechanical properties of the SOD@SiO_2_ film. a) Fatigue resistance of SOD@SiO_2_ film with 1000 successive loading–unloading cycles under a constant strain of 30%. b) Summarized maximum stress and dissipated energy of SOD@SiO_2_ film during the cyclic stretching–releasing process.


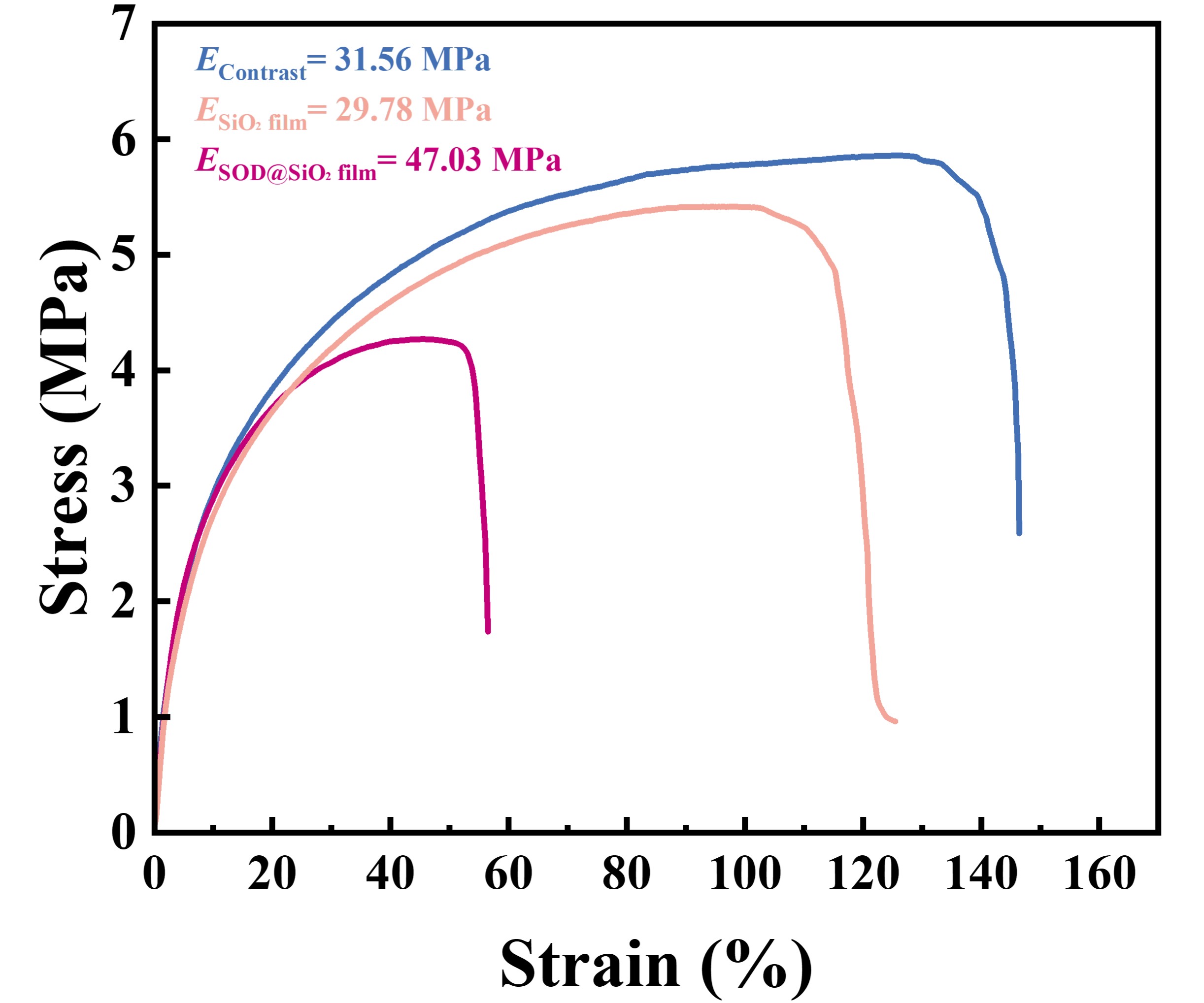


# **Figure S28.** The stress-strain curves of SiO_2_, SOD@SiO_2_ and blank films. (The blank film is composed exclusively of resin.)


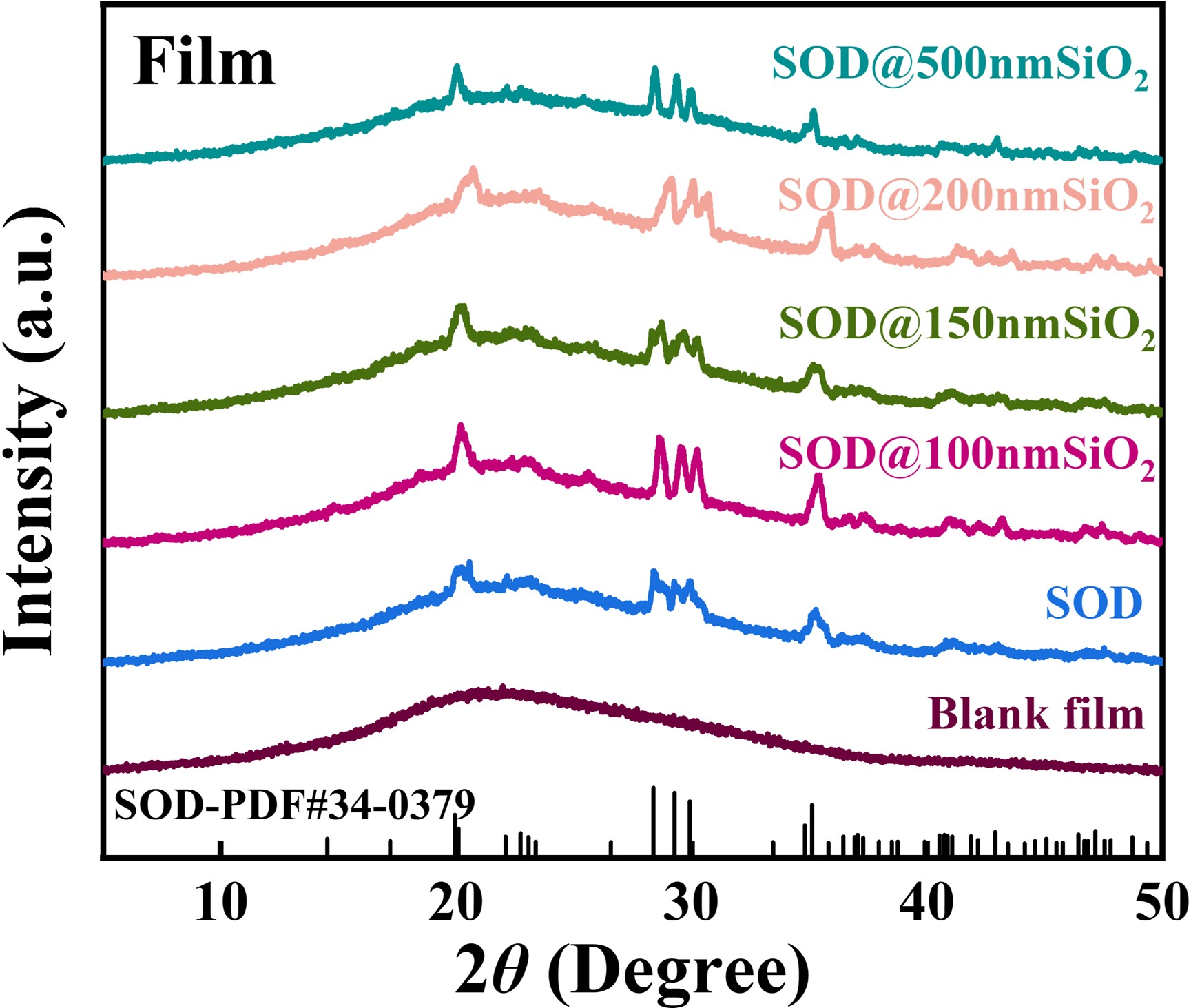


# **Figure S29.** XRD patterns of SOD and SOD@SiO_2_ films.

# **Table S2.** Cost evaluation of SOD@SiO_2_ film.

All material and processing costs are calculated in US dollars ($) based on laboratory-scale synthesis.

|  | Ingredients | Unit price | Usage amount | Price ($) |
| --- | --- | --- | --- | --- |
|  | **SOD** | 2.06$/g | 0.187 g | 0.385 |
|  | **PMMA** | 0.14$/g | 0.3 g | 0.042 |
|  | **TPU** | 0.4$/g | 0.66 g | 0.264 |
|  | **TTMAP** | 0.13$/g | 0.1 g | 0.013 |
|  | **DMF** | 0.0044$/mL | 2.11 mL | 0.0092 |
|  | **Acetone** | 0.0051$/mL | 1.32 mL | 0.0067 |
|  | **EtOH** | 0.0021$/mL | 0.0138 mL | 0.00003 |
|  | **NH_4_OH** | 0.0025$/mL | 0.0007 mL | 0.000002 |
|  | **Ethyl silicate** | 0.015$/mL | 0.00412 mL | 0.00006 |
|  | **H_2_O** | 0.00025$/mL | 0.00138 mL | 0.0000003 |
| Total cost of SOD@SiO_2_ film | **0.0033 $/ cm^2^**  **(yield 221 cm^2^)** | | | |


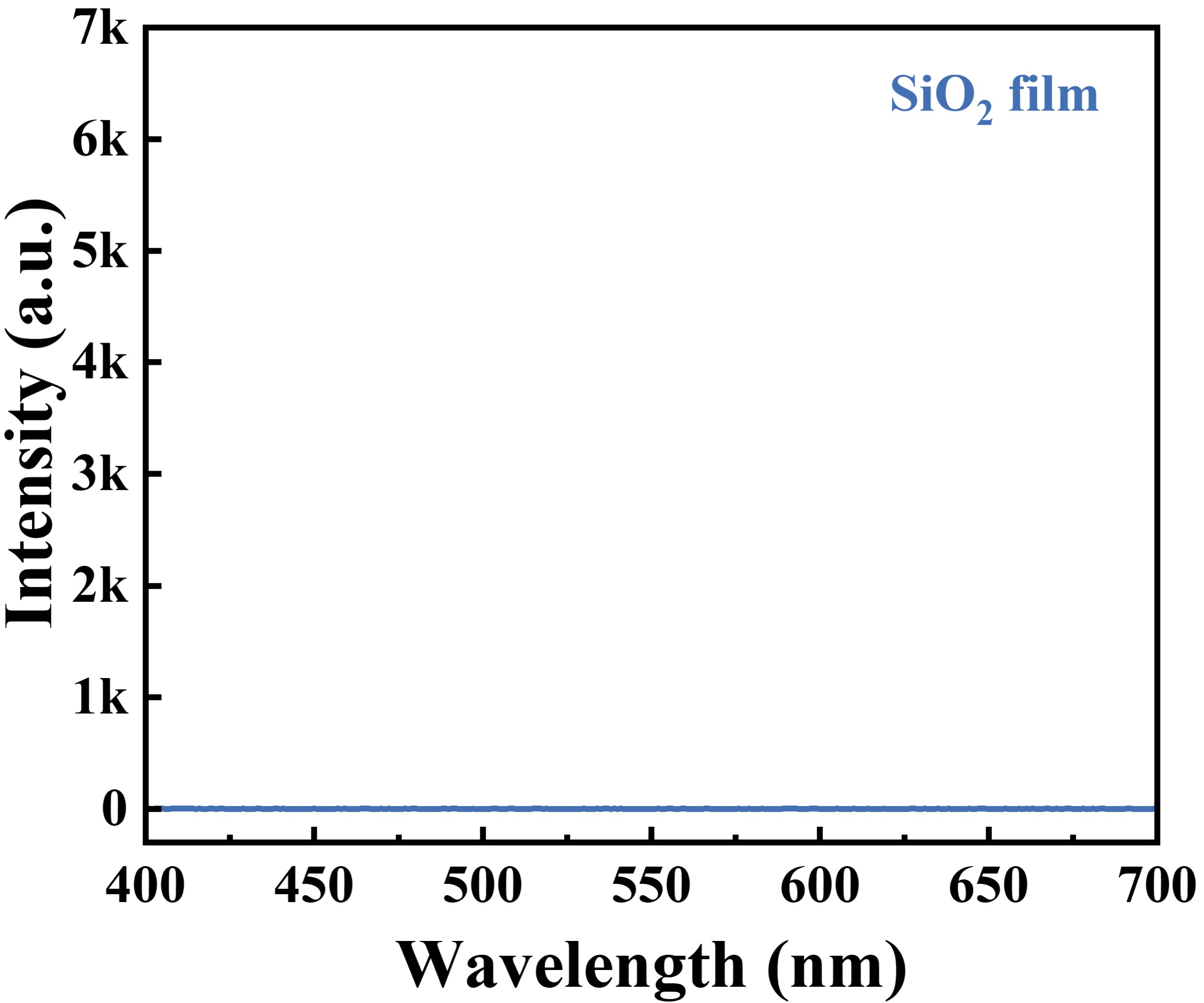


# **Figure S30.** The PL spectrum of SiO_2_ film. *λ_ex_ = 365 nm*


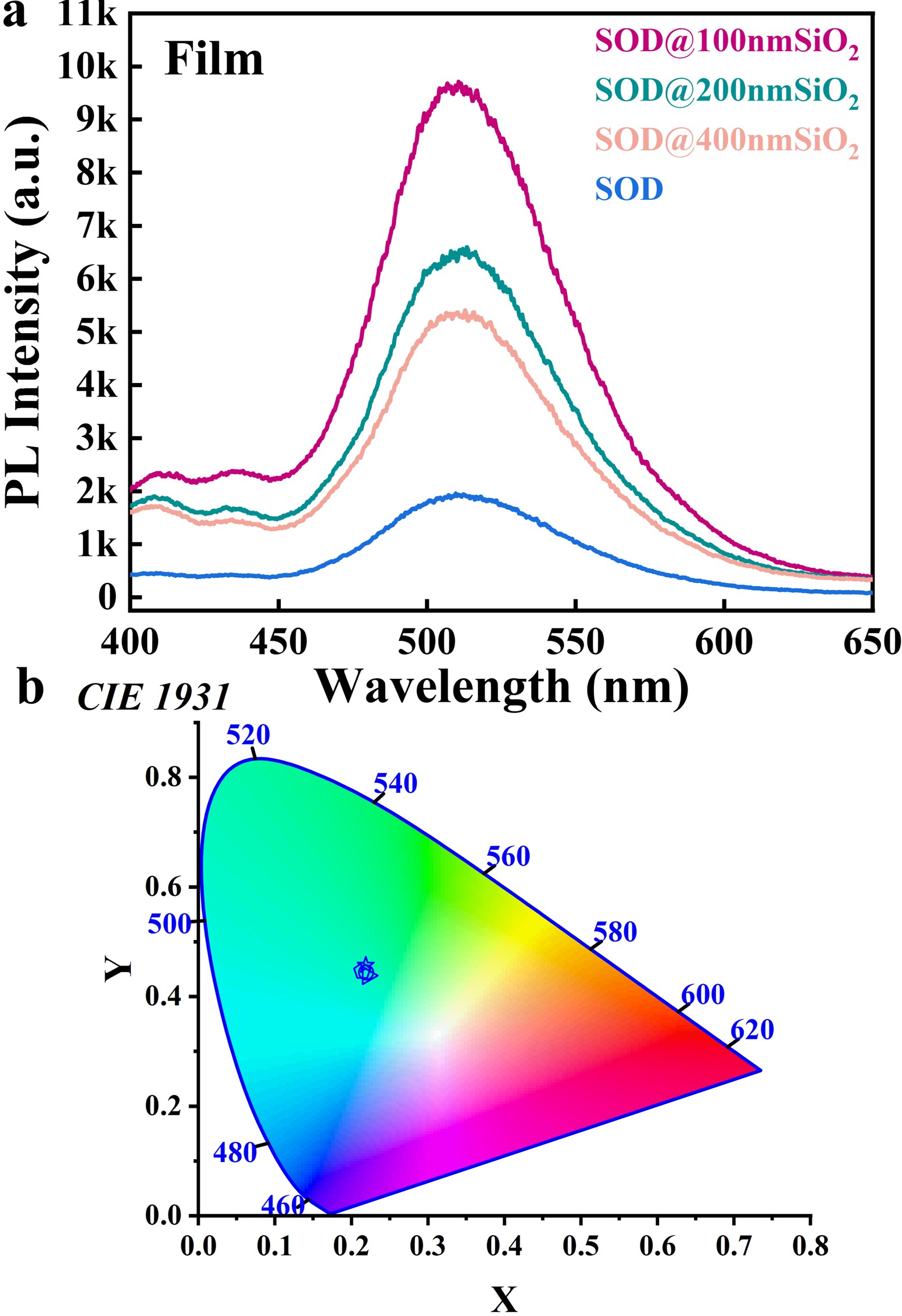


**Figure S31.** The PL spectra and CIE coordinates of SOD and SOD@SiO_2_ films. a) PL spectra and b) Corresponding CIE coordinates. *λ_ex_ = 365 nm*


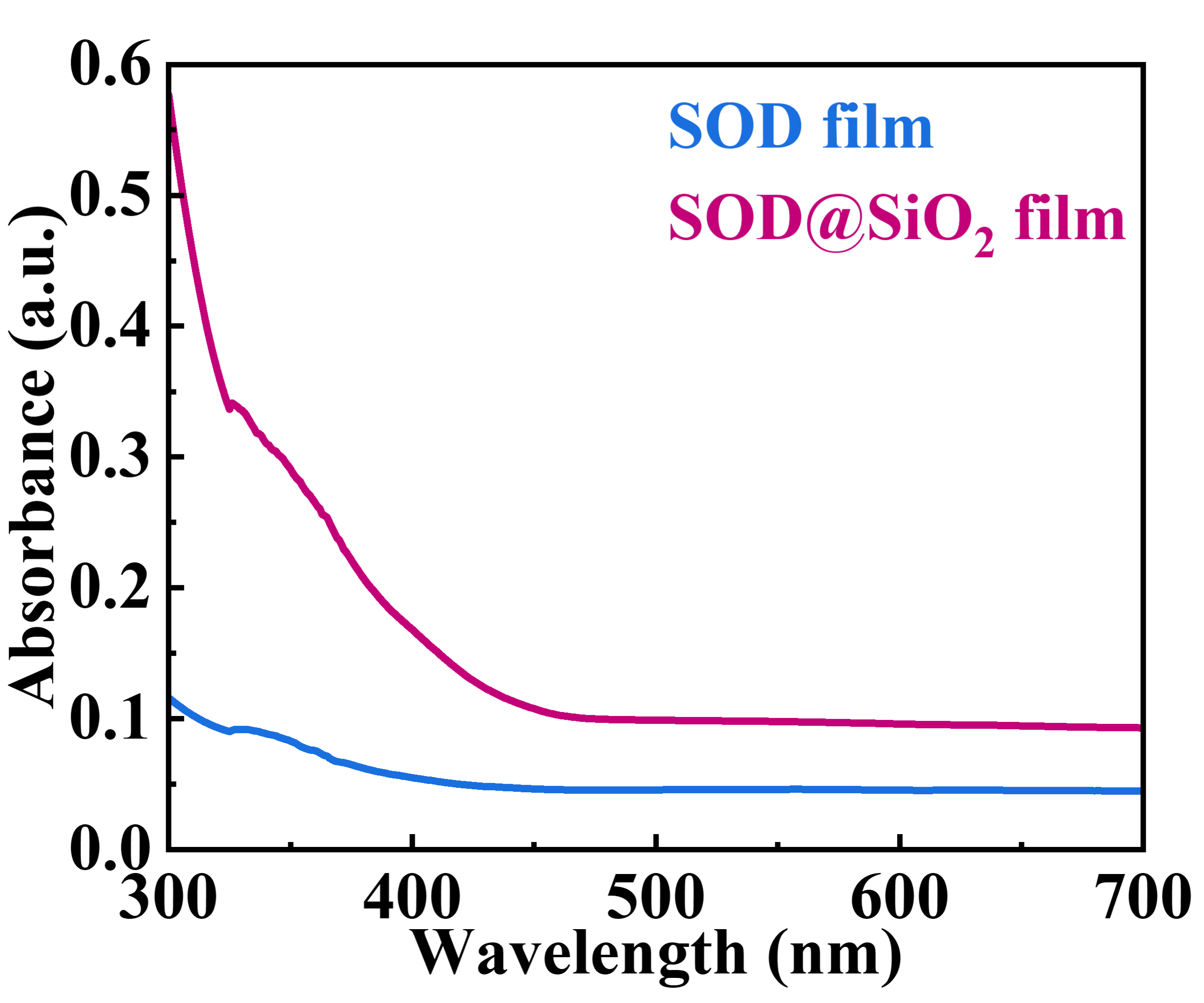


# **Figure S32.** The UV-Vis absorbance spectra of SOD and SOD@SiO_2_ films.


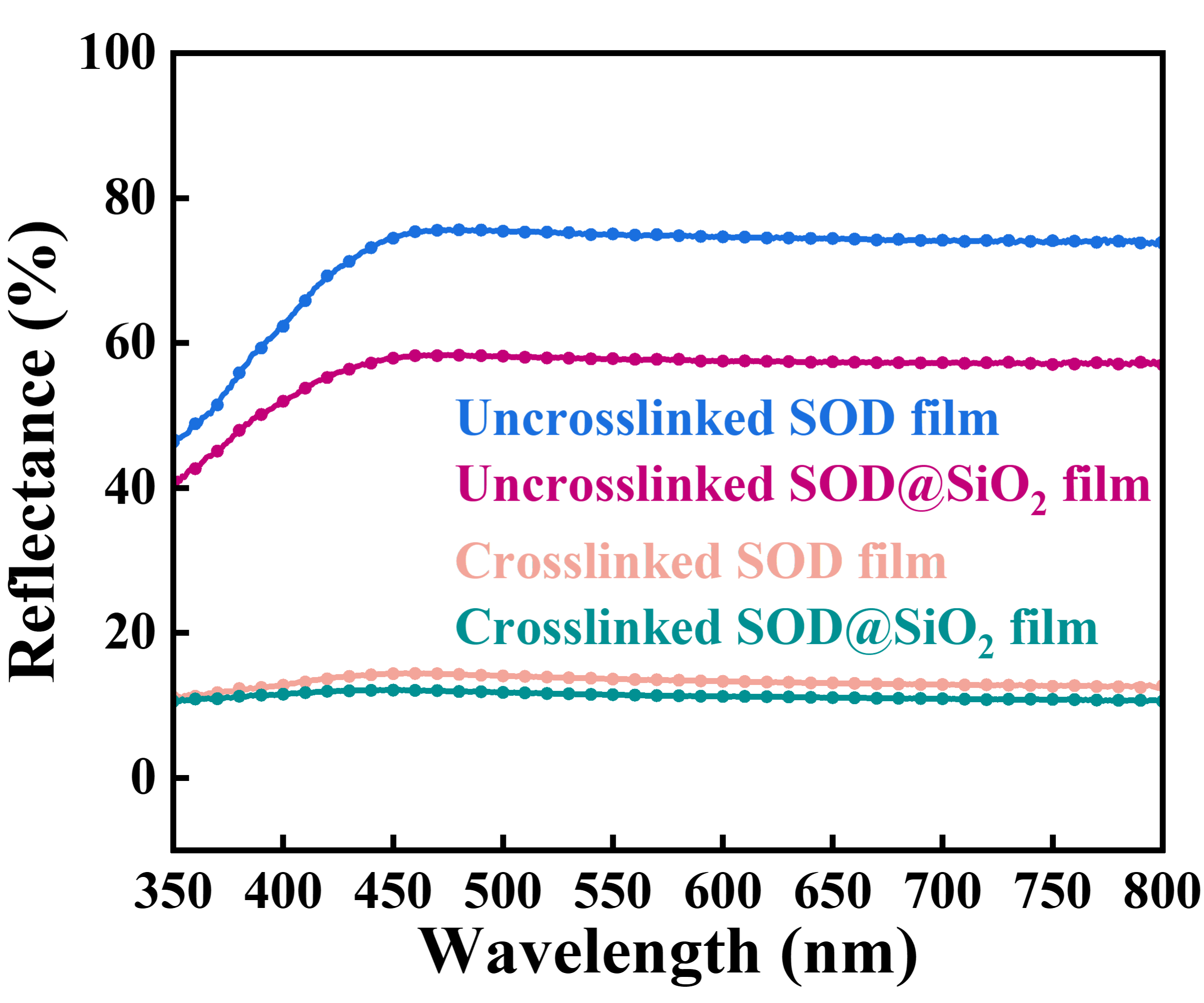


# **Figure S33.** Diffuse reflectance spectroscopy of the corresponding film.


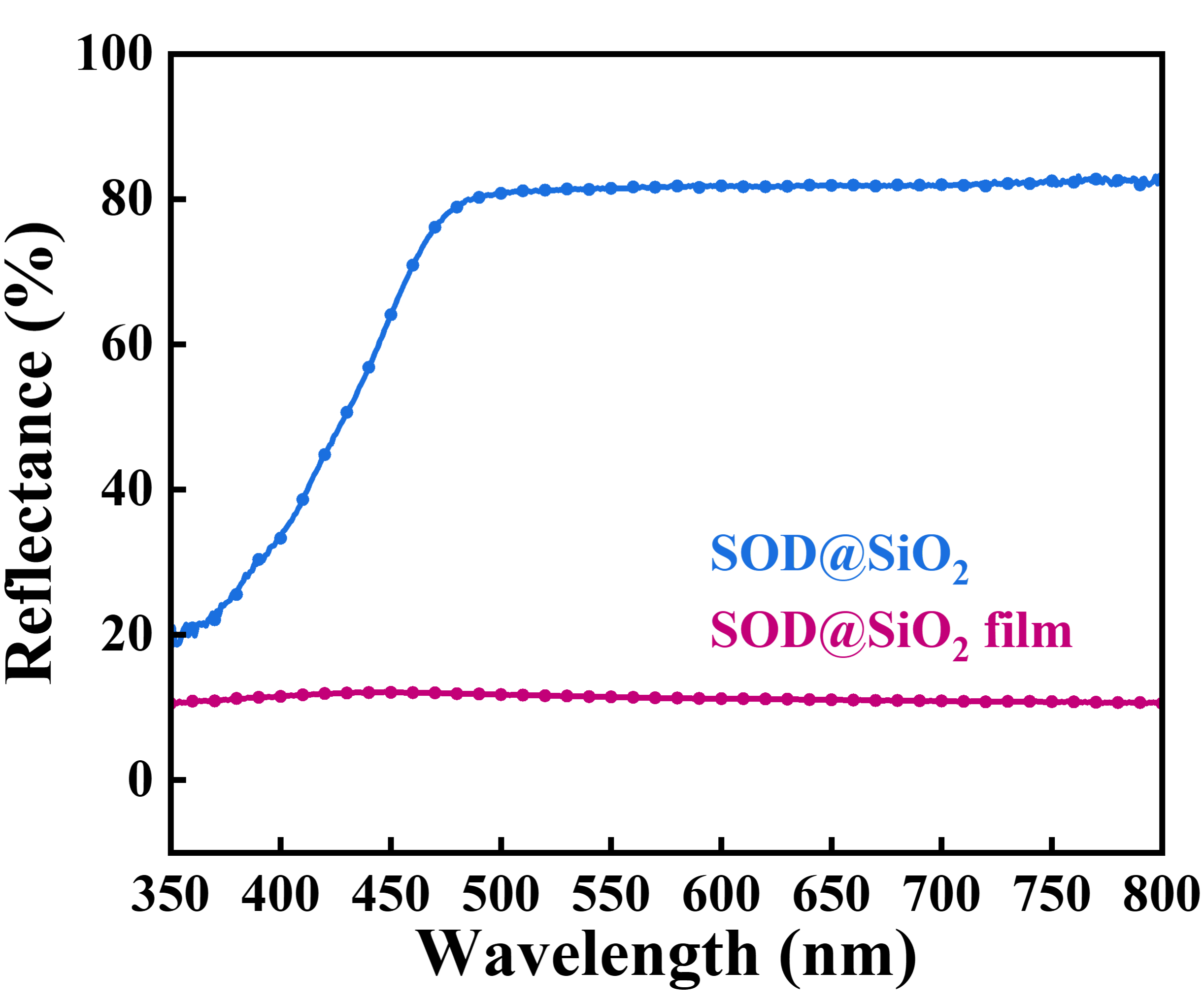


# **Figure S34.** Diffuse reflectance spectroscopy of SOD@SiO_2_ powders and SOD@SiO_2_ film.


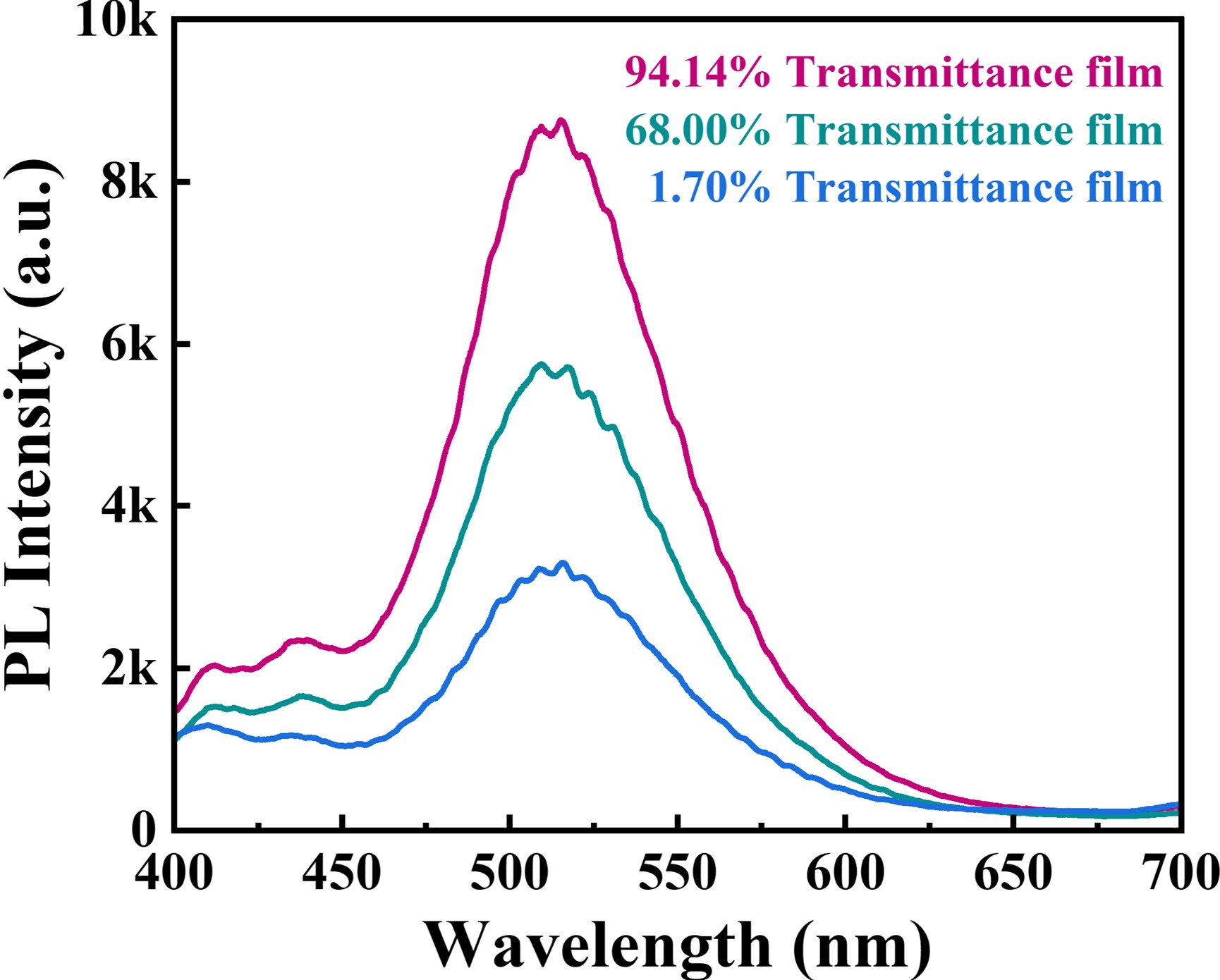


# **Figure S35.** PL spectra of SOD@SiO_2_ films with varying optical transmittance. *λ_ex_ = 365 nm*


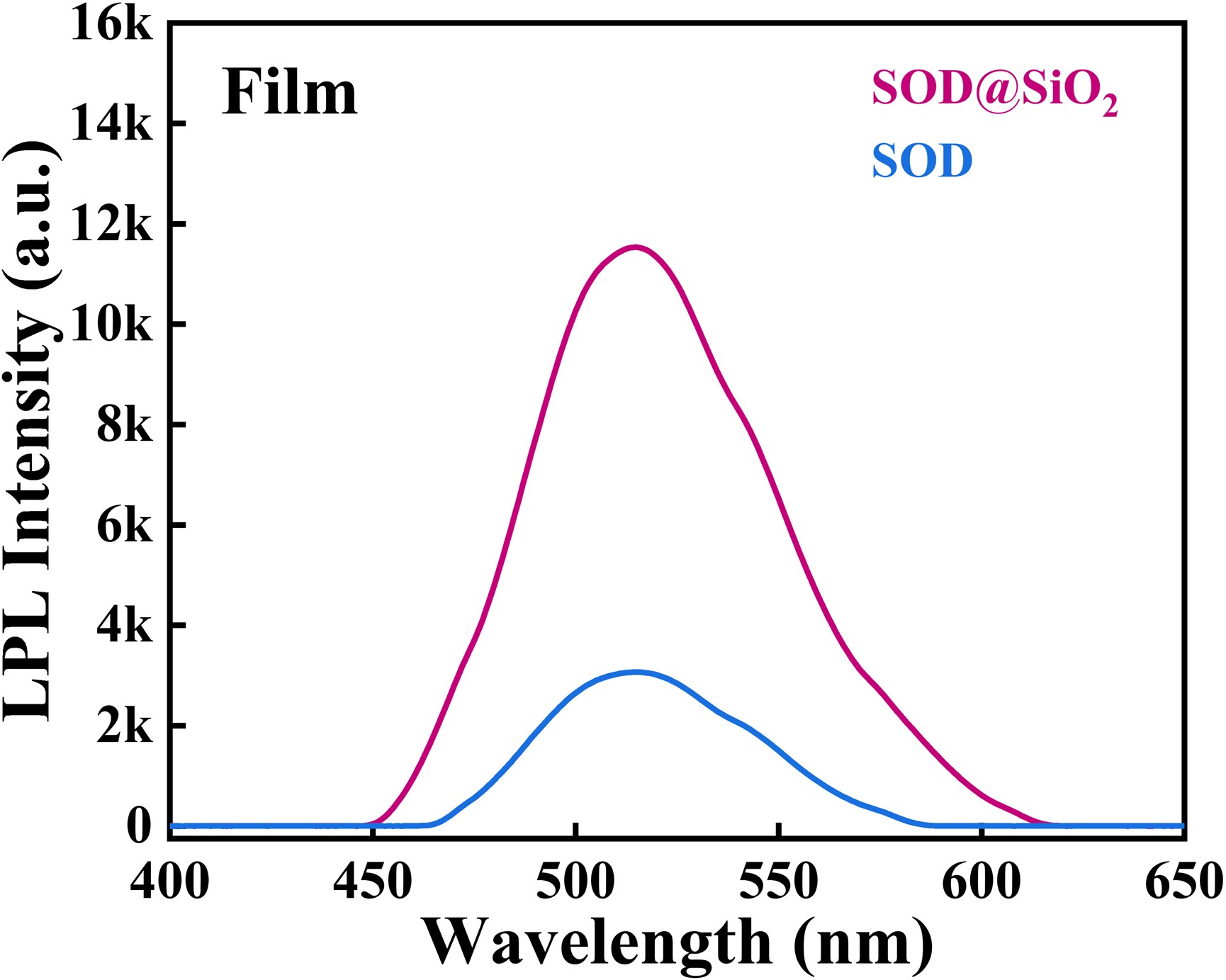


# **Figure S36.** The afterglow spectrum of the SOD and SOD@SiO_2_ film. *λ_ex_ = 365 nm*


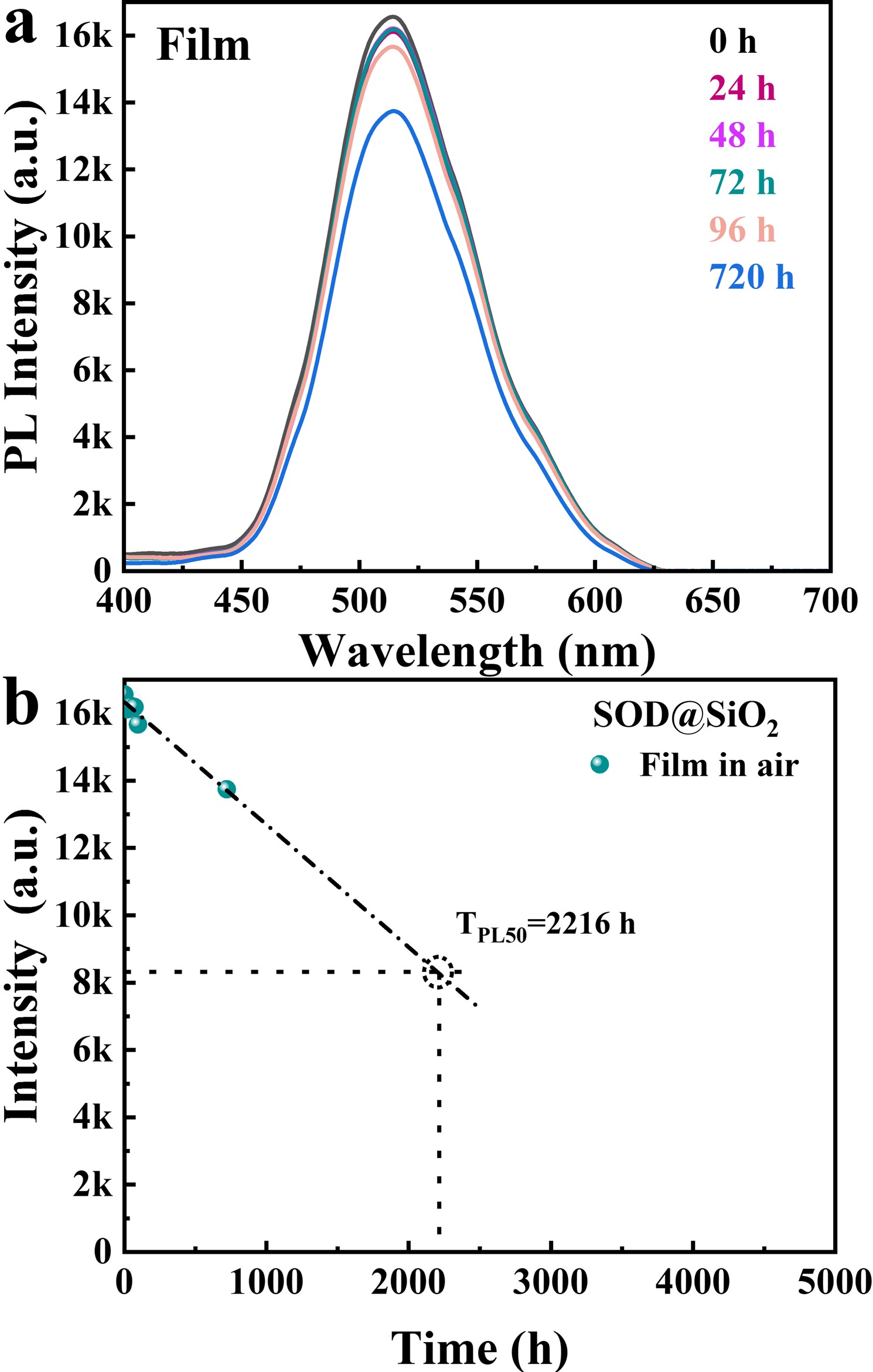


# **Figure S37.** PL intensity tracking of SOD@SiO_2_ films under ambient storage. a) The PL spectra of SOD@SiO_2_ films at different aging times b) *T*_PL50_ was calculated by fitting the SOD@SiO_2_ film stored at room temperature.


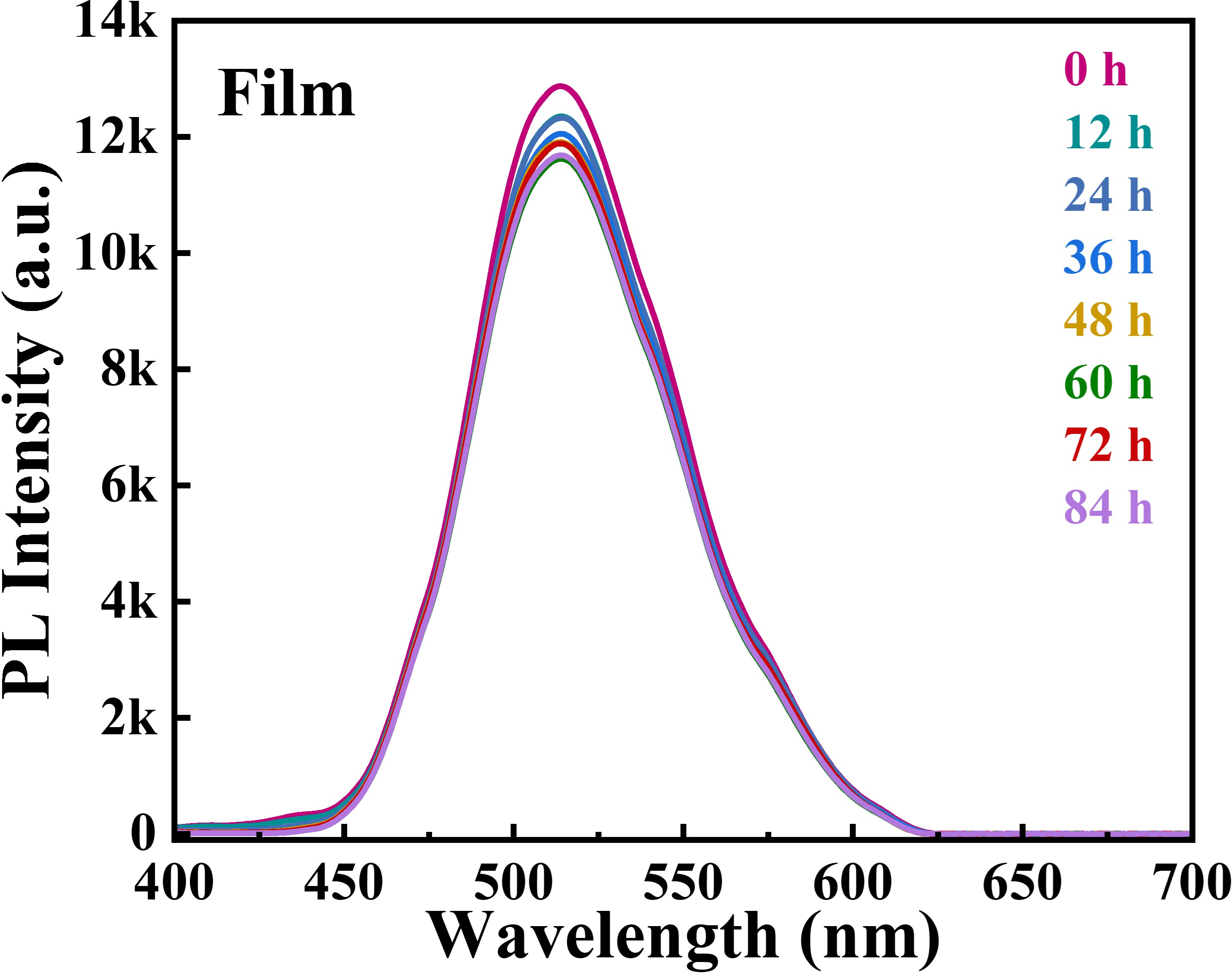


# **Figure S38.** Evaluation of the as-prepared SOD@SiO_2_ films under continuous 365nm irradiation.


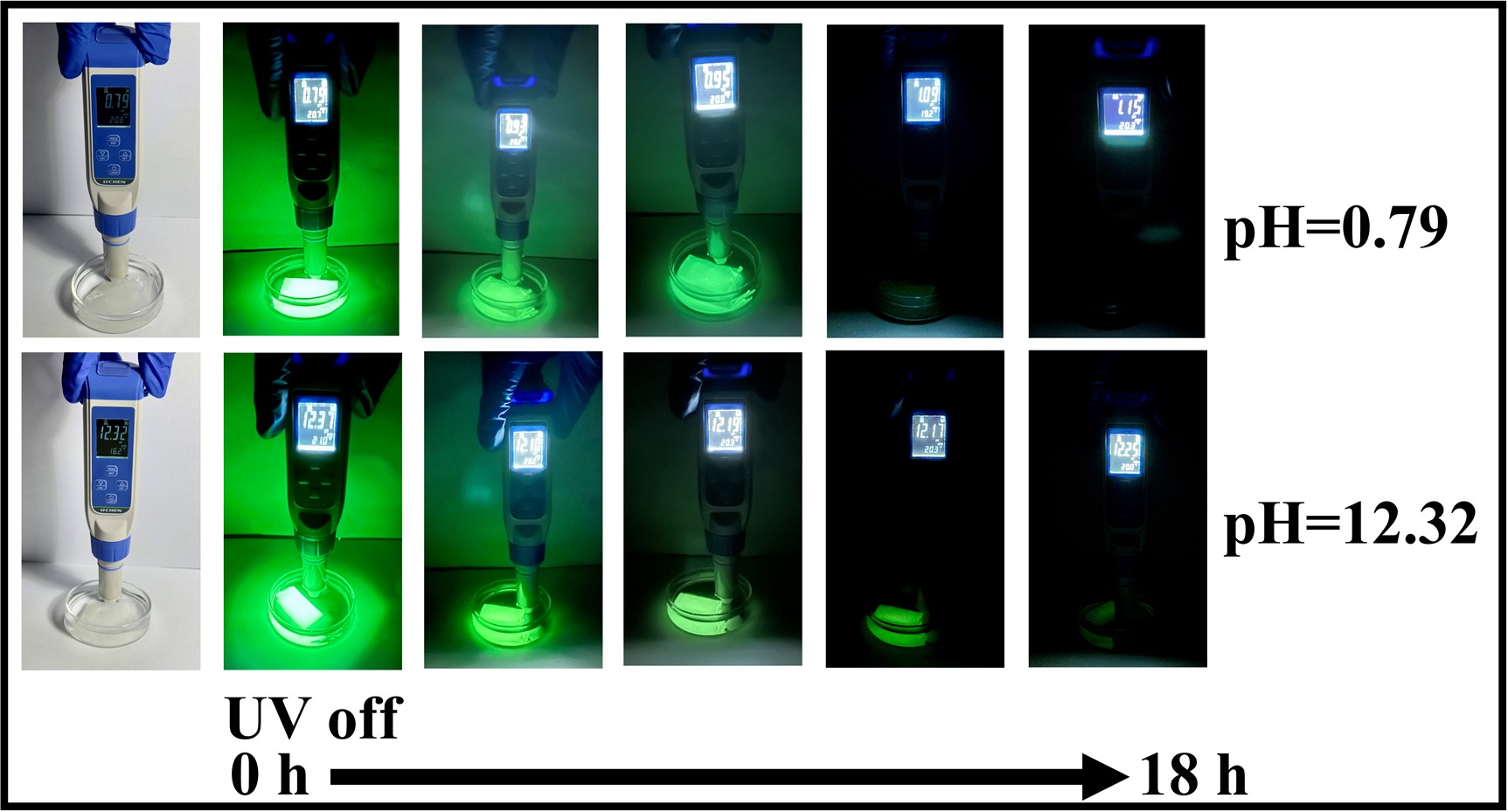


# **Figure S39.** The afterglow performance of SOD@SiO_2_ films in strong acidic solution (pH=0.79) and strong alkaline solution(pH=12.32).


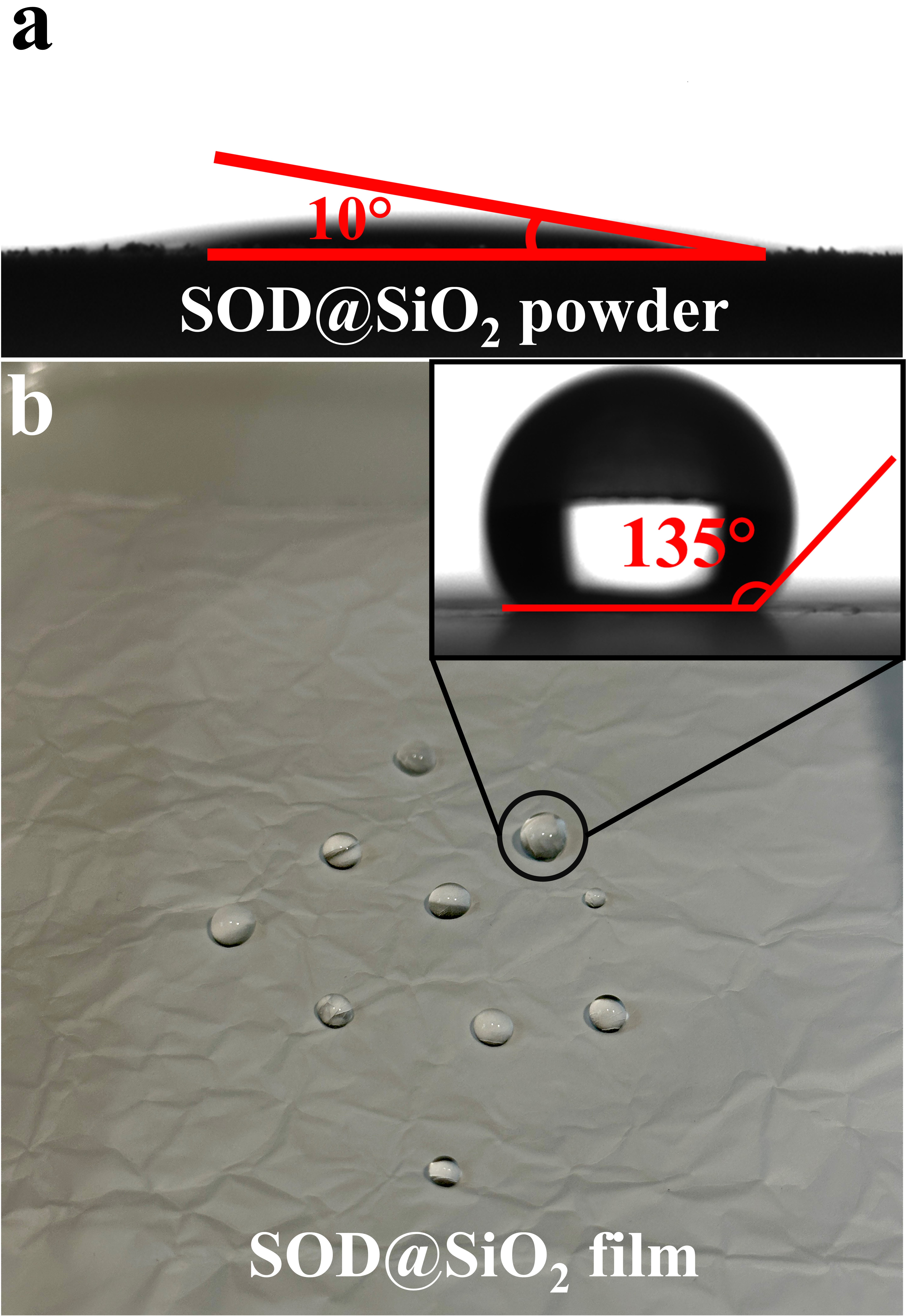


# **Figure S40.** Contact angle analysis. a) SOD@SiO_2_ powder and b) SOD@SiO_2_ film.


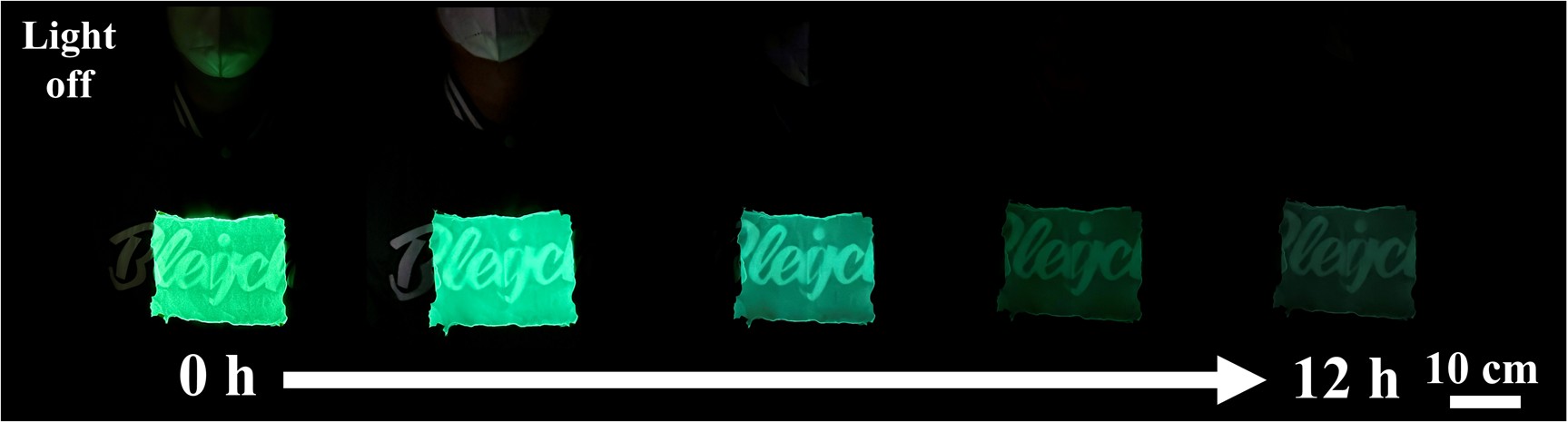


# **Figure S41.** Demonstration of transparent and large-area SOD@SiO_2_ film as a component of protective clothing with long afterglow.


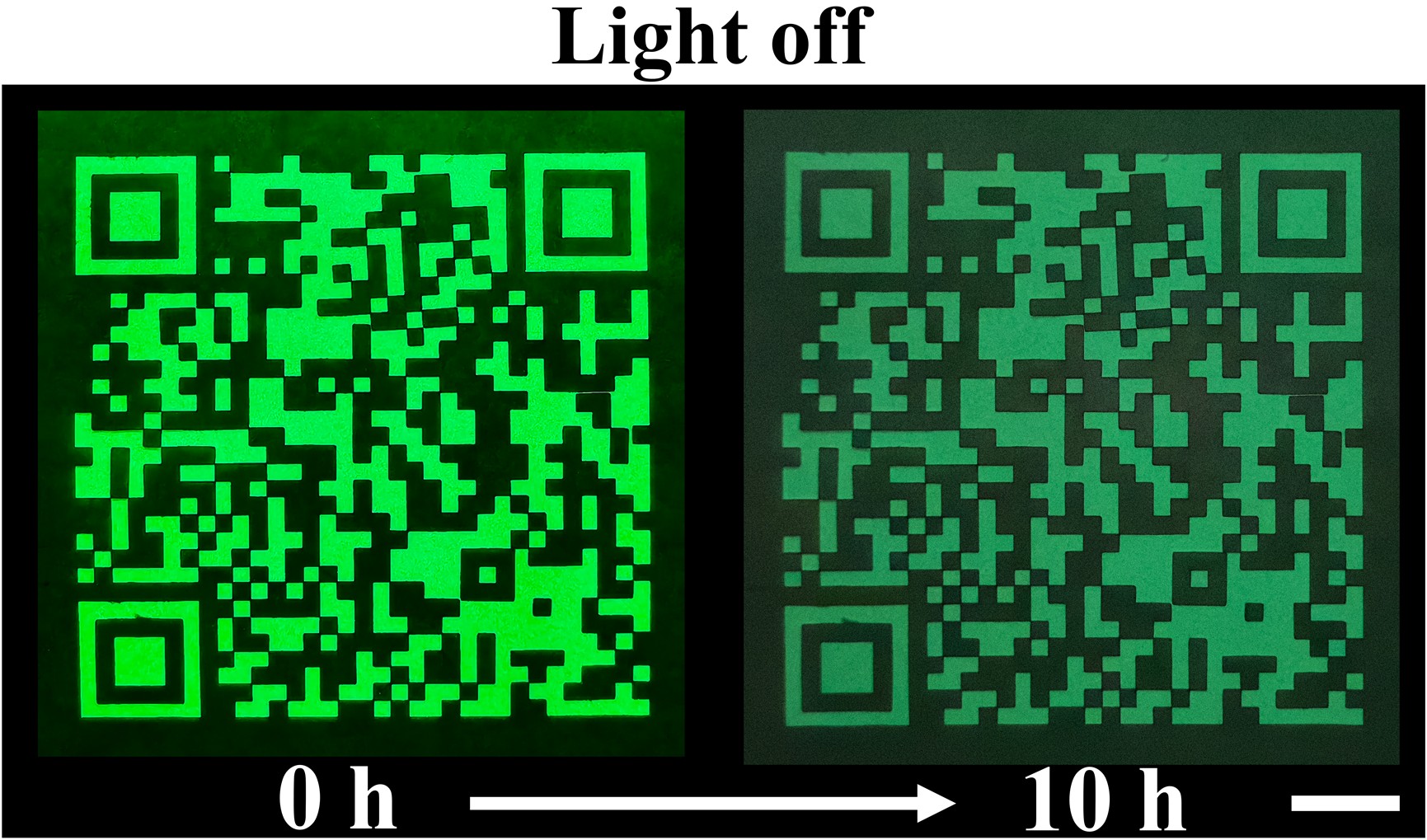


# **Figure S42.** Demonstration of SOD@SiO_2_ film application. A QR code printed on a SOD@SiO_2_ film for solar-energy storage and LPL releasing.


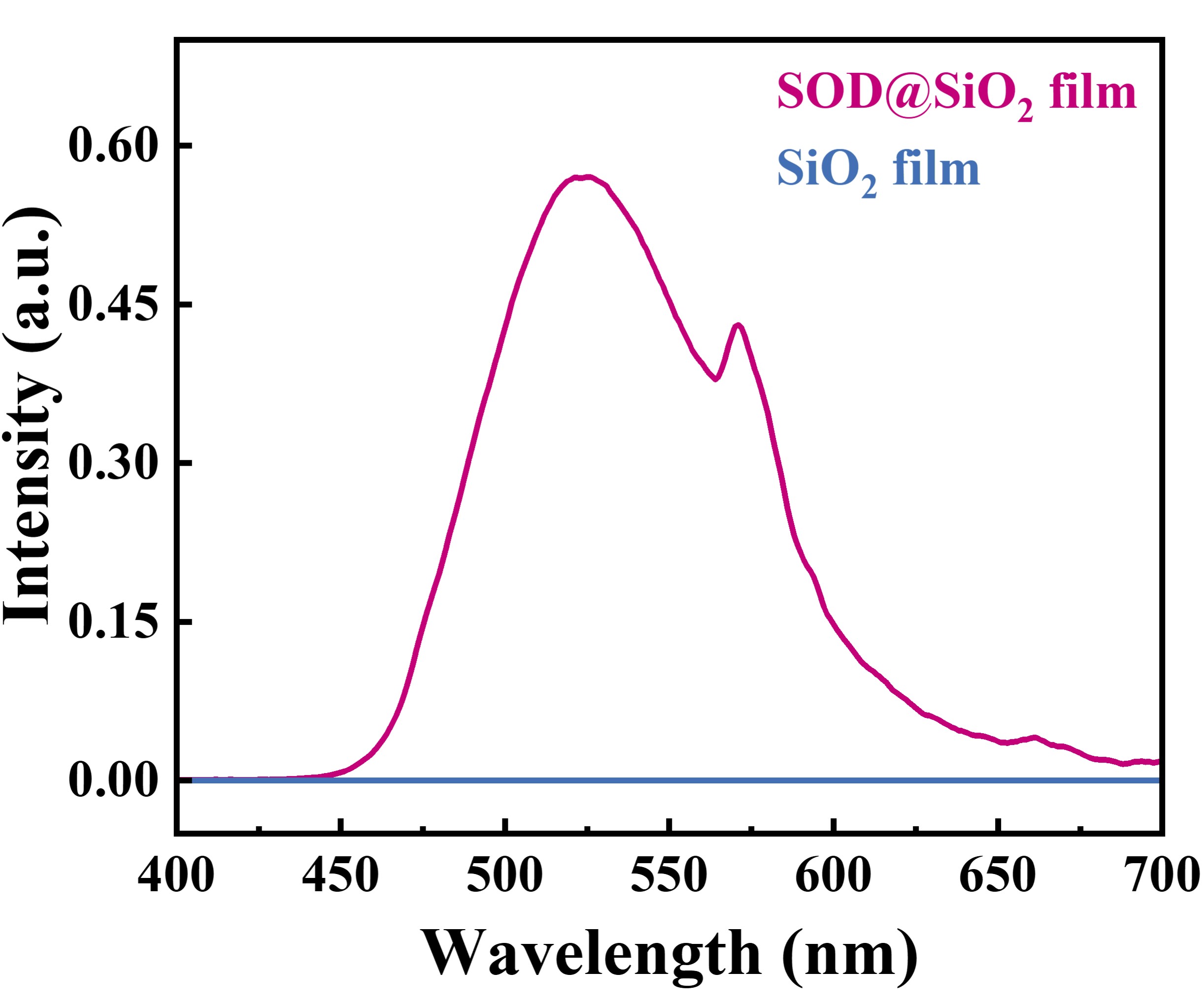


# **Figure S43.** The RL spectra of SiO_2_ film and SOD@SiO_2_ film.


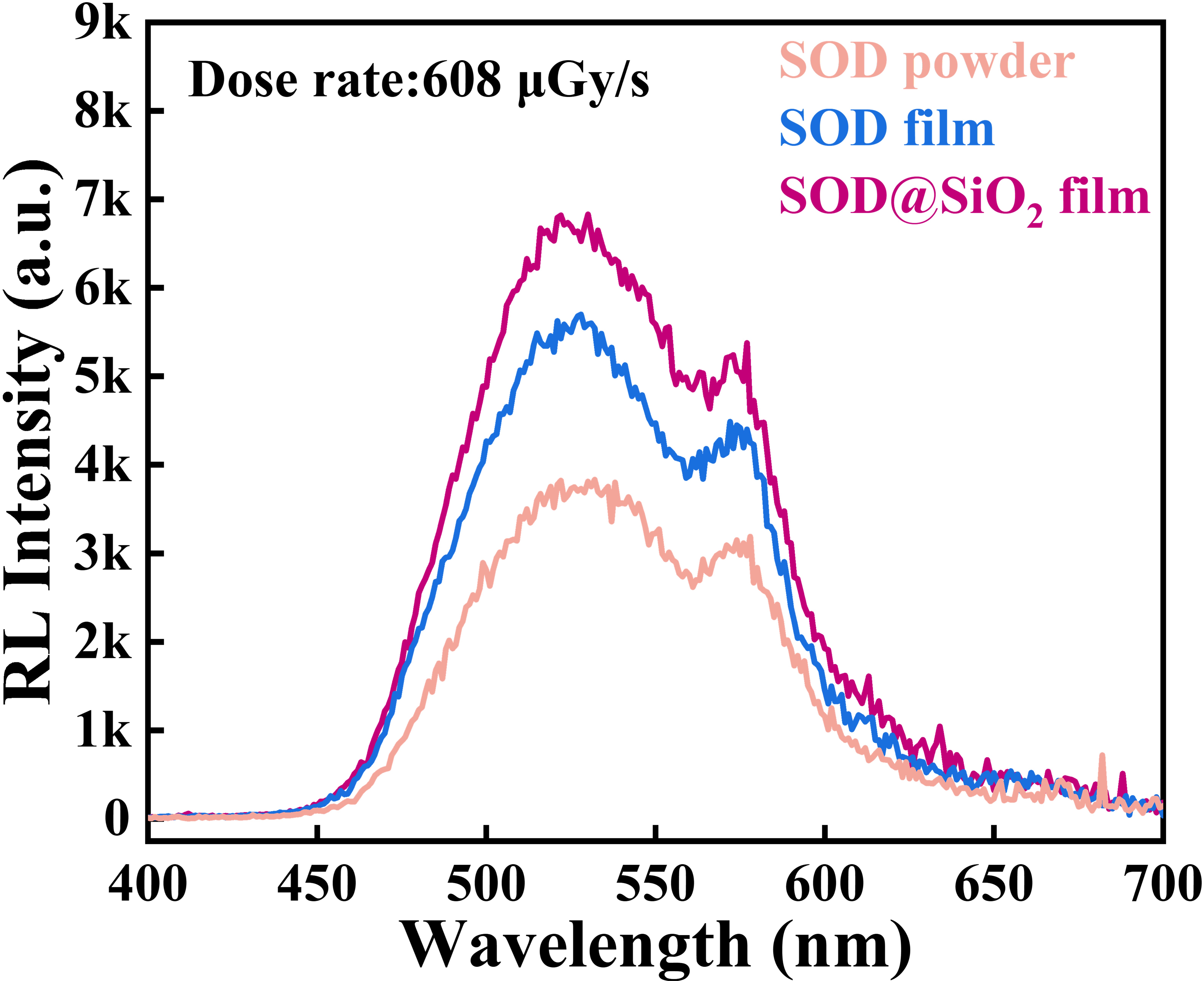


# **Figure S44.** RL spectra of SOD powder, SOD film and SOD@SiO_2_ film.

X-ray dose rate: 608 μGy/s.


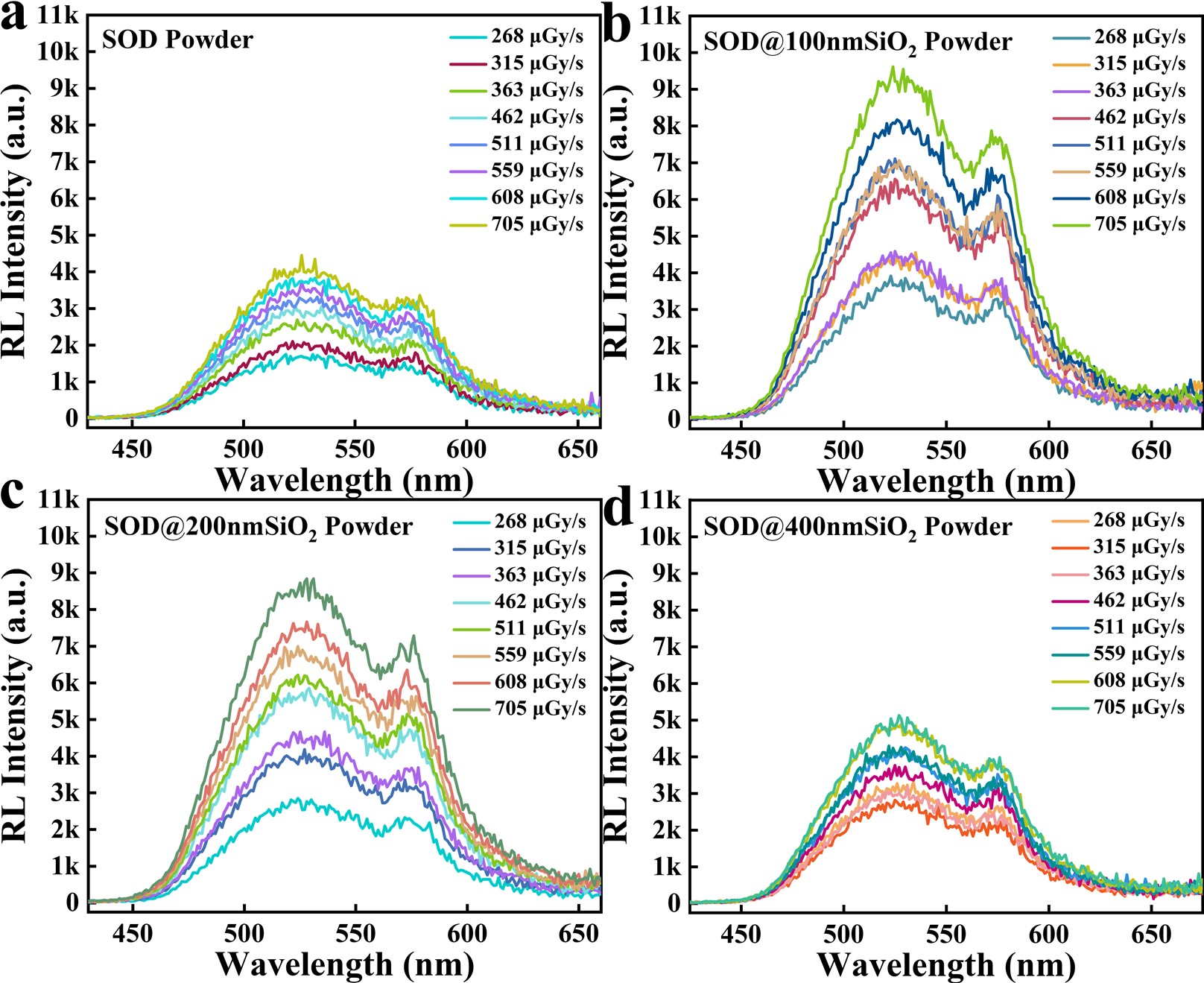


# **Figure S45.** RL spectra of SOD and SOD@SiO_2_ powder under various X-ray dose rate. a) SOD; b) SOD@100nmSiO_2_; c) SOD@200nmSiO_2_; d) SOD@400nmSiO_2_.

# **Table S3.** Summary of detection limits.

| **Samples** | **Emission**  **Peak (nm)** | **Background**  **S. D.** | **Slope** | **Detection limits**  **(μGy/s)** |
| --- | --- | --- | --- | --- |
| SOD | 525 | 1.81 | 6.82 | 0.79 |
| SOD@SiO_2_ | 525 | 1.81 | 14.94 | 0.36 |
| SOD film | 525 | 1.81 | 10.1 | 0.54 |
| SOD@SiO_2_ film | 525 | 1.81 | 12.5 | 0.43 |

**S. D. stands for standard deviation.**

**See Methods for the calculation of detection limits.**

**
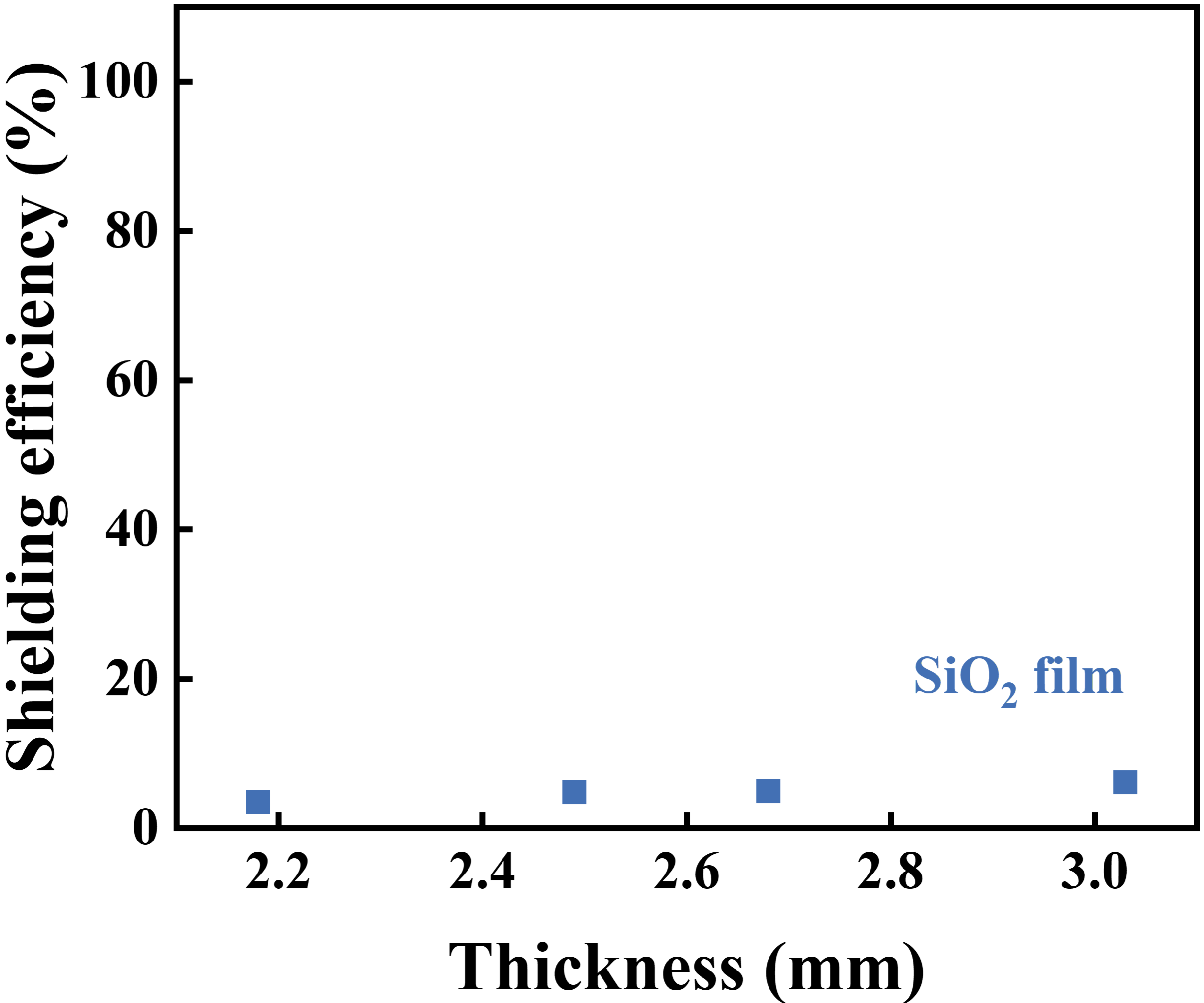
**

# **Figure S46.** X-ray shielding efficiency of SiO_2_ film.


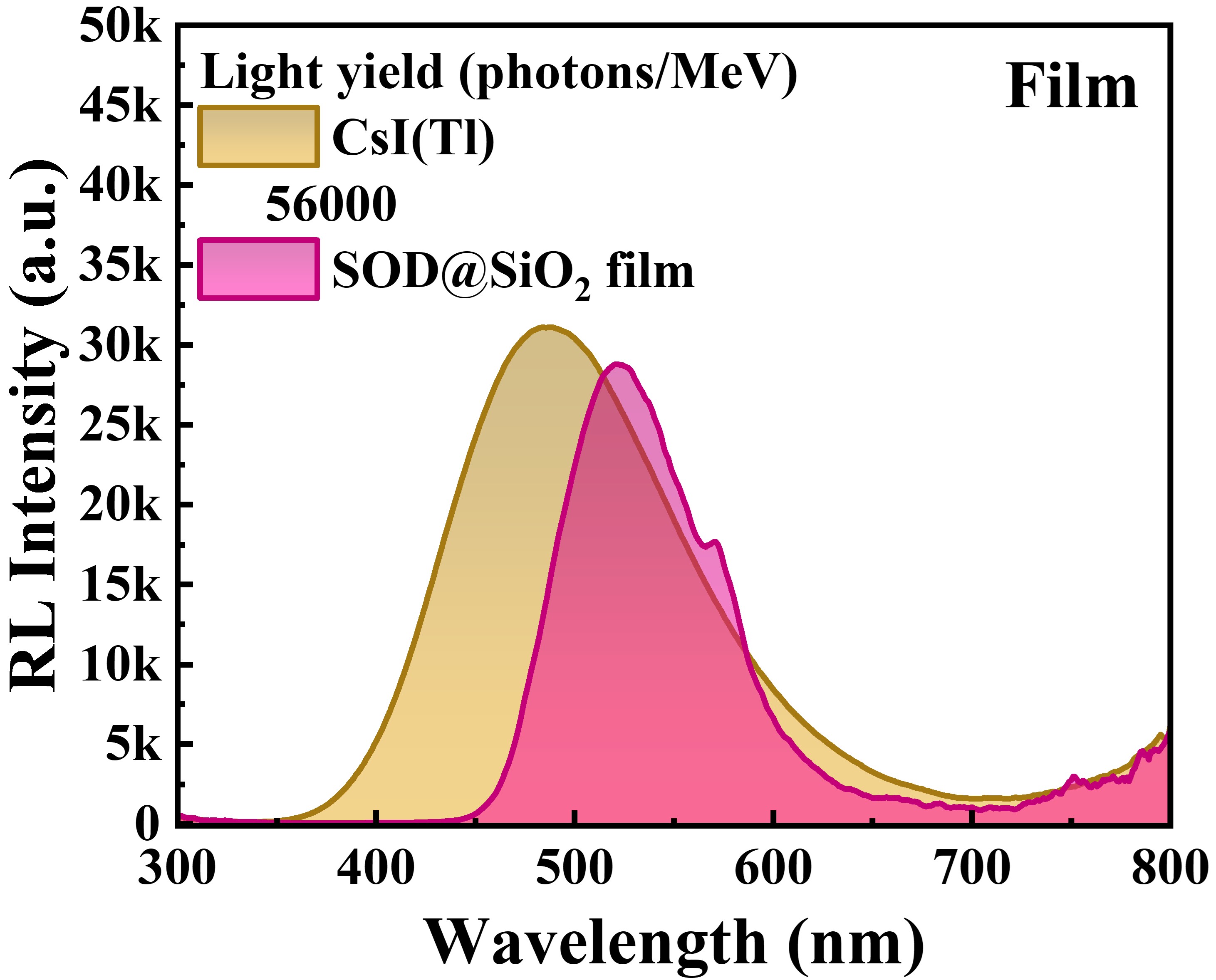


# **Figure S47.** RL spectra of SOD@SiO_2_ film. Sample thickness: 1 mm (same as CsI(Tl) reference).


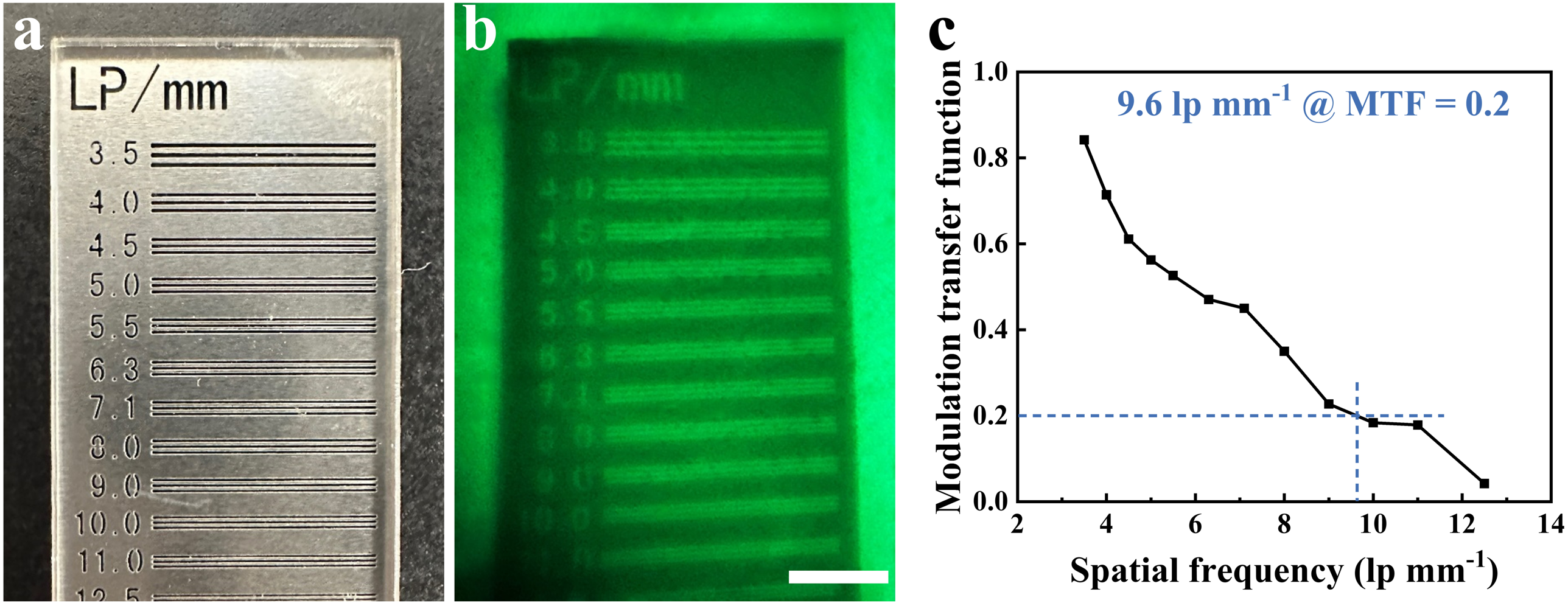


# **Figure S48.** Images of a resolution phantom under a) natural light and b) X-ray (50 kV, 200 μA, scale bar: 4 mm). c) Modulation transfer function (MTF) of the imaging panel as a function of spatial frequency.


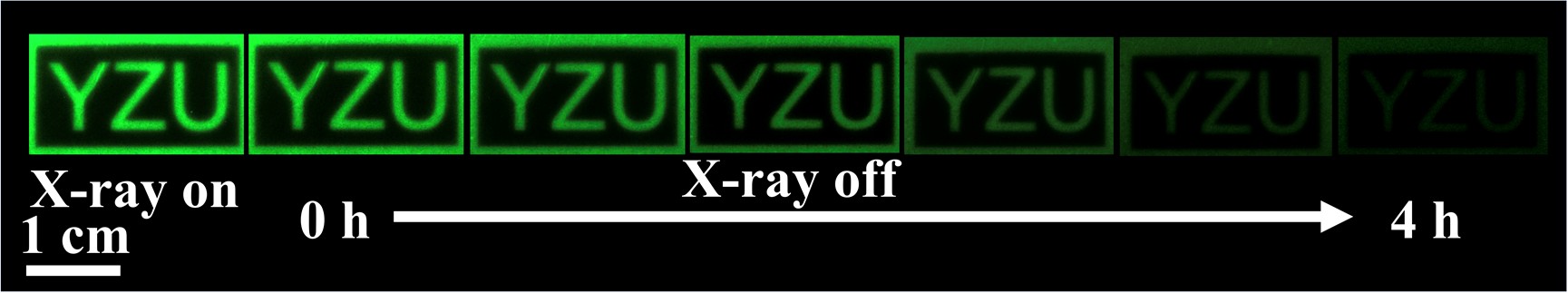


# **Figure S49.** Demonstrate the time-lapse imaging function of the transparent and large-area SOD@SiO_2_ film, excited by 30 W, 60 kV X-ray light.
